# A multifactorial model of pathology for age of onset heterogeneity in familial Alzheimer’s disease

**DOI:** 10.1007/s00401-020-02249-0

**Published:** 2020-12-14

**Authors:** Diego Sepulveda-Falla, Lucia Chavez-Gutierrez, Erik Portelius, Jorge I. Vélez, Simon Dujardin, Alvaro Barrera-Ocampo, Felix Dinkel, Christian Hagel, Berta Puig, Claudio Mastronardi, Francisco Lopera, Bradley T. Hyman, Kaj Blennow, Mauricio Arcos-Burgos, Bart de Strooper, Markus Glatzel

**Affiliations:** 1 Institute of Neuropathology, University Medical Center Hamburg-Eppendorf, Hamburg, Germany; 2 Neuroscience Group of Antioquia, Faculty of Medicine, University of Antioquia, Medellín, Colombia; 3 VIB Center for Brain and Disease Research, 3000 Leuven, Belgium; 4 Department of Neurology, KU Leuven, Leuven, Belgium; 5 Institute of Neuroscience and Physiology, Dept. of Psychiatry and Neurochemistry, The Sahlgrenska Academy At the University of Gothenburg, Mölndal, Sweden; 6 Clinical Neurochemistry Laboratory, Sahlgrenska University Hospital, Mölndal, 431 80 Mölndal, Sweden; 7 Department of Genome Sciences, John Curtin School of Medical Research, Australian National University, Canberra, ACT Australia; 8 Universidad del Norte, Barranquilla, Colombia; 9 Department of Neurology, Massachusetts General Hospital, Harvard Medical School, MassGeneral Institute for Neurodegenerative Disease, Charlestown, USA; 10 Facultad de Ciencias Naturales, Departamento de Ciencias Farmaceuticas, Universidad Icesi, Grupo Natura, Calle 18 No. 122 -135, Cali, Colombia; 11 GIPSI Group, Department of Psychiatry, Medical Research Institute, University of Antioquia, Medellín, Colombia; 12 UK Dementia Research Institute, University College London, Queen Square, London, WC1N 3BG UK

## Abstract

Presenilin-1 (PSEN1) mutations cause familial Alzheimer’s disease (FAD) characterized by early age of onset (AoO). Examination of a large kindred harboring the PSEN1-E280A mutation reveals a range of AoO spanning 30 years. The pathophysiological drivers and clinical impact of AoO variation in this population are unknown. We examined brains of 23 patients focusing on generation and deposition of beta-amyloid (Aβ) and Tau pathology profile. In 14 patients distributed at the extremes of AoO, we performed whole-exome capture to identify genotype–phenotype correlations. We also studied kinome activity, proteasome activity, and protein polyubiquitination in brain tissue, associating it with Tau phosphorylation profiles. PSEN1-E280A patients showed a bimodal distribution for AoO. Besides AoO, there were no clinical differences between analyzed groups. Despite the effect of mutant PSEN1 on production of Aβ, there were no relevant differences between groups in generation and deposition of Aβ. However, differences were found in hyperphosphorylated Tau (pTau) pathology, where early onset patients showed severe pathology with diffuse aggregation pattern associated with increased activation of stress kinases. In contrast, late-onset patients showed lesser pTau pathology and a distinctive kinase activity. Furthermore, we identified new protective genetic variants affecting ubiquitin–proteasome function in early onset patients, resulting in higher ubiquitin-dependent degradation of differentially phosphorylated Tau. In PSEN1-E280A carriers, altered γ-secretase activity and resulting Aβ accumulation are prerequisites for early AoO. However, Tau hyperphosphorylation pattern, and its degradation by the proteasome, drastically influences disease onset in individuals with otherwise similar Aβ pathology, hinting toward a multifactorial model of disease for FAD. In sporadic AD (SAD), a wide range of heterogeneity, also influenced by Tau pathology, has been identified. Thus, Tau-induced heterogeneity is a common feature in both AD variants, suggesting that a multi-target therapeutic approach should be used to treat AD.

**Supplementary Information:**

The online version contains supplementary material available at 10.1007/s00401-020-02249-0.

## Introduction

Age-associated dementias, such as Alzheimer’s disease (AD), constitute a growing burden for modern society. AD presents with a wide spectrum of clinical features, and we have only recently begun to understand its pathophysiological basis. According to the amyloid hypothesis for AD, generation and aggregation of beta-amyloid peptide (Aβ) represent the initiator and main factor in a cascade of events which include oxidative stress, synaptic dysfunction, mitochondrial dysfunction, and hyperphosphorylation of Tau protein (pTau) leading to neurodegeneration [25, 39, 45]. All familial AD (FAD) disease-causing mutations in Presenilin 1 (PSEN1), Presenilin2 (PSEN2), and Amyloid precursor protein (APP) relate to Aβ production or aggregation, which is one of the main arguments supporting the amyloid hypothesis [19, 25]. Mutations in both PSEN1 and PSEN2 favor the production of aggregation-prone Aβ peptides [10, 52]. The fact that length and ratios of Aβ peptides are altered in PSEN1 FAD brain tissue, cell culture, and in murine models [10, 25] reinforces the concept of their contribution to the pathophysiology of FAD. Thus, FAD serves as a model for sporadic AD (SAD) in clinical trials [45] and genetically modified organisms with FAD-causing mutations are often used in laboratory experiments [27].

Clinically, SAD and FAD differ, not only regarding AoO but also in symptoms and development of disease. For example, with most of the cases from ~ 200 different PSEN1 mutations, disease starts before 60 years of age with prodromal stages detected as early as 30 years of age [30]. Generally, AoO in FAD is early, yet considerable AoO variability exists in PSEN1 FAD [1, 30, 44]. AoO is widely variable and considered as a marker of disease severity in AD regardless of etiology [14]. So far, genetic modifiers such as APOε4 allele frequency, Aβ deposition patterns, and in vitro analysis of Aβ1-42 production have failed to correlate with disease onset in PSEN1 E280A FAD, suggesting the presence of other modifiers [30, 46]. Recent studies in SAD demonstrate that different patterns of tau phosphorylation are associated with both age of onset and rate of clinical progression of disease [15], and that tau pathology is the driver of disease severity instead of Aβ pathology burden [36], suggesting that a similar phenomenon may help to explain the heterogeneity of AoO in this PSEN1 cohort. Lately, we have also identified several genetic variants linked to AoO variation, including APOε2 allele frequency, but only some of them relate to known mechanisms of disease [28, 29, 57–59]. Most affected families with PSEN1 mutations comprise a limited number of carriers. The Colombian PSEN1 E280A kindred is exceptionally large, with more than 5000 individuals and more than 1000 living carriers permitting long-term clinical follow-up studies [1, 46] and presymptomatic clinical trials [45]. Nevertheless, even in this genetically uniform population [4], AoO ranges from 39 to 70 years of age, and clinical symptoms are highly variable with language impairment, epileptic seizures, and cerebellar dysfunction as main symptoms besides memory impairment and AD type dementia [46]. Thus, this cohort is ideally suited to investigate the pathophysiological basis underlying differences in AoO in AD. Here, we identified Tau hyperphosphorylation and its degradation by the proteasome as AoO modifiers in PSEN1 E280A patients by linking genetic, morphological, and biochemical findings with clinical profiles.

## Methods

### Study design

The PSEN1 E280A families’ follow-up makes part of an ongoing retrospective observational cohort study. Data collection is unrestricted, and it is limited only to availability of information about each patient. Demographic and clinical data are confirmed by multiple sources, and when no source is available, or information is not reliable, it is consigned as “NA”, not available. The objectives of the study were to identify pathological phenotypes associated with AoO variability in the PSEN1 E280A population. Experimental analyses were conducted blind when possible and technical replicates were performed for all experiments limited to sample availability. All data obtained were used in the analyses including outliers.

### Patients and clinical data collection

FAD patients belong to the PSEN1 E280A Antioquia cohort study. All participants or their guardians provided written informed consent for participation in the study. The study was approved by the medical ethics board of the University of Antioquia, Colombia. Follow-up, cognitive assessment, cognitive domains analysis, and genotyping were performed as previously described [1]. For neuropathological studies, AD brain samples were collected from their brain bank. All procedures were performed following ethical board approval from the university and informed consent was acquired for postmortem brain donations. Familial cases were selected according to sample quality, availability, and short postmortem time. Sporadic AD cases were used as pathological positive controls in the experiments aimed to evaluate pathological phenotypes. Healthy controls were used to establish a non-pathological baseline in biochemical studies. Sporadic cases were selected based on clinical diagnosis of probable AD, lack of family history of dementia, and tested as non-carriers for PSEN1 mutations. Control cases were selected based on lack of brain trauma, and cognitive or neurological symptoms before death (for a detailed description, see Supplementary Materials, online resource).

### Morphological methods

All morphological analyses were performed on 3-μm-thick de-paraffinized sections from cortices of 10 SAD and 23 FAD cases (Supplementary Table 3, online resource). Immunohistochemical staining and quantifications were performed as published using listed antibodies (Supplementary Tables 16 and 17, online resource). Ultrastructural analysis was performed using glutaraldehyde-fixed cerebellar tissue from 3 SAD and 3 FAD patients as previously described [47]. Temporal cortex samples were excised from paraformaldehyde fixed tissue after localizing specific areas of extracellular pTau deposits or an equivalent area from LOFAD cases. Samples were fixed with glutaraldehyde and chrome-osmium, dehydrated in ethanol, and embedded in Epon 812 (Serva Electrophoresis GmbH). After polymerization, 1-μm-thick sections were cut, stained with toluidine blue, and checked for presence of amyloid plaques. To further process them for electron microscopy, relevant specimens were cut into 60–80-nm-thick sections, which were contrasted with uranyl acetate and lead solution. Sections were viewed under an LEO EM 912AB electron microscope (Zeiss). Formalin-fixed 500-µm-thick temporal cortex samples from 5 EOFAD, 5 AOFAD, and 5 LOFAD cases were clarified using a CLARITY protocol as previously described [35] and stained with a Synaptophysin primary antibody (Supplementary Table. 16, online resource). A Z-stack of a minimum thickness of 100 µm was acquired with a Leica TCS SP5 confocal microscope (Leica microsystems, Wetzlar, Germany). 3D synaptophysin-positive particle counting was performed using the 3D object counting plug-in on ImageJ 1.52p (NIH, USA).

### Biochemical methods

Total protein homogenates from temporal cortex from 10 SAD and 23 FAD patients were prepared following standard protocols. Soluble and insoluble fractions from temporal cortex from nine SAD and 23 FAD patients were isolated as described by Tremblay et al. [55]. SDS-PAGE, tricine gel electrophoresis, immunoprecipitation, and western blotting were performed according to standard methods (see Supplementary materials and methods, online resource). Primary and secondary antibodies are listed in Supplementary Tables 16 and 17, online resource). Mass spectrometry of Aβ peptides was performed by immunoprecipitating homogenized frontal cortex from six SAD and 23 FAD patients (Supplementary Table 3 and extended methods, online resource). De novo generation of Aβ peptides was assessed in detergent-resistant membranes isolated from frontal cortex of five controls and 23 FAD brains using a standardized published protocol [53]. Kinase activity profiles were determined using the PamChip ® 96 serine/threonine (STK) and protein tyrosine (PTK) peptide microarray system from PamGene International B.V. (‘s-Hertogenbosch, The Netherlands) according to the instructions of the manufacturer, and as published previously [43]. Chymotrypsin 20S proteasome activity was tested in temporal cortex from four controls and 14 FAD cases using the 20S proteasome activity assay kit APT280 (Millipore-Merck, Darmstadt, Germany), following the manufacturer instructions. Finally, frozen temporal cortices were homogenized and centrifuged at 10,000×*g* for 10 min at 4 °C. The supernatant was collected, and protein concentration was assessed. Briefly, The Tau RD P301S FRET Biosensor (ATCC® CRL-3275™) cells stably expressing the repeat domain of Tau with the P301S mutation conjugated to either the cyan fluorescent protein (CFP) or the yellow fluorescent protein (YFP) (TauRD-P301S-CFP/YFP) were cultured and plated on 96-well plates at a density of 40,000 cells per well. 1 µg of total protein from brain extracts were then incubated with Lipofectamine 2000 in Opti-MEM (Thermo Fisher Scientific, Waltham, MA, USA) for 10 min at room temperature before being added to the cells. After 24 h, cells were trypsinized, fixed, and run on the MACSQuant VYB (Miltenyi) flow cytometer. CFP and Forster resonance energy transfer (FRET) were both measured by exciting the cells using the 405 nm laser and reading fluorescence emission at the 405/50 nm and 525/50 nm filters, respectively. FRET signal was quantified, and 40,000 events per well were analyzed. Data were analyzed using the MACSQuantify software (Miltenyi, Auburn, CA, USA).

### Genetic methods

Fourteen patients with PSEN1-E280A FAD placed at the extremes of AoO distribution were included for whole-exome capture (WEC) and in-depth genetic association statistical analysis, performed as described previously [58]. DNA libraries were constructed from 1 μg of genomic DNA using an Illumina TruSeq genomic DNA library kit (Illumina Inc., San Diego, CA, USA). Exons were enriched from the pooled 3 μg of library DNA using an Illumina TruSeq Exome enrichment kit (Illumina Inc.). Each exome-enriched pool was run on a 100-base-pair paired-end run on an IlluminaHiSeq 2000 sequencer (Illumina Inc.). All regions were sampled at ~ 50Xcoverage. Sequencing image data were processed in real time using Illumina Real-Time Analysis (RTA) software (Illumina Inc., San Diego, CA, USA), and converted to suitable formats using the CASAVA pipeline from Illumina. The resulting FASTQ files were further processed for variant analysis using Golden Helix®’s SNP variation suite (SVS) 8.3.0 (Golden Helix, Inc. Bozeman, MT, USA). Genotype files were processed in SVS 8.3.0. De novo SNVs were defined according to the DNA-seq Analysis module in SVS 8.3.0. Potential relationships between AoO and SNVs were individually examined using one-way analysis of variance (ANOVA). P values were obtained based on the F-statistic and corrected for multiple testing using the false discovery rate (FDR) and a method based on extreme-value theory, as explained elsewhere [58]. Network analysis and pathway analysis were performed using NetworkAnalyst [64] webpage tools and Cytoscape software [48]. Protein–protein interaction was assessed InnateDB [9] webpage tools.

### General statistical analysis

Data was analyzed using IBM SPSS Statistics 22 software (IBM/SPSS Inc., Armonk NY, USA), GraphPad Prism 6 (GraphPad Software, Inc., La Jolla, CA, USA), and R statistical software (R Foundation for Statistical Computing, Vienna, Austria). Analyses included distribution analysis, Hartigan’s dip test, nonparametric tests, and *χ*^2^ squared test for categorical variables comparisons. ANOVA and logistic regression analysis were applied to AoO, cognitive variables and schooling time. One-way ANOVA and Kruskal–Wallis test were used for group comparison for demographic, neuropathological, and biochemical variables. The Mann–Whitney U (given as Z) nonparametric test was used for two group comparisons, when indicated. Correlation analysis was performed using Pearson correlation coefficient and Spearman’s ρ test. Statistical significance of all analyses was determined with *p ≤ 0.05, ** p ≤ 0.01, and ***p ≤ 0.001.

## Results

### AoO distribution in PSEN1 E280A FAD patients

**Fig. 1:**
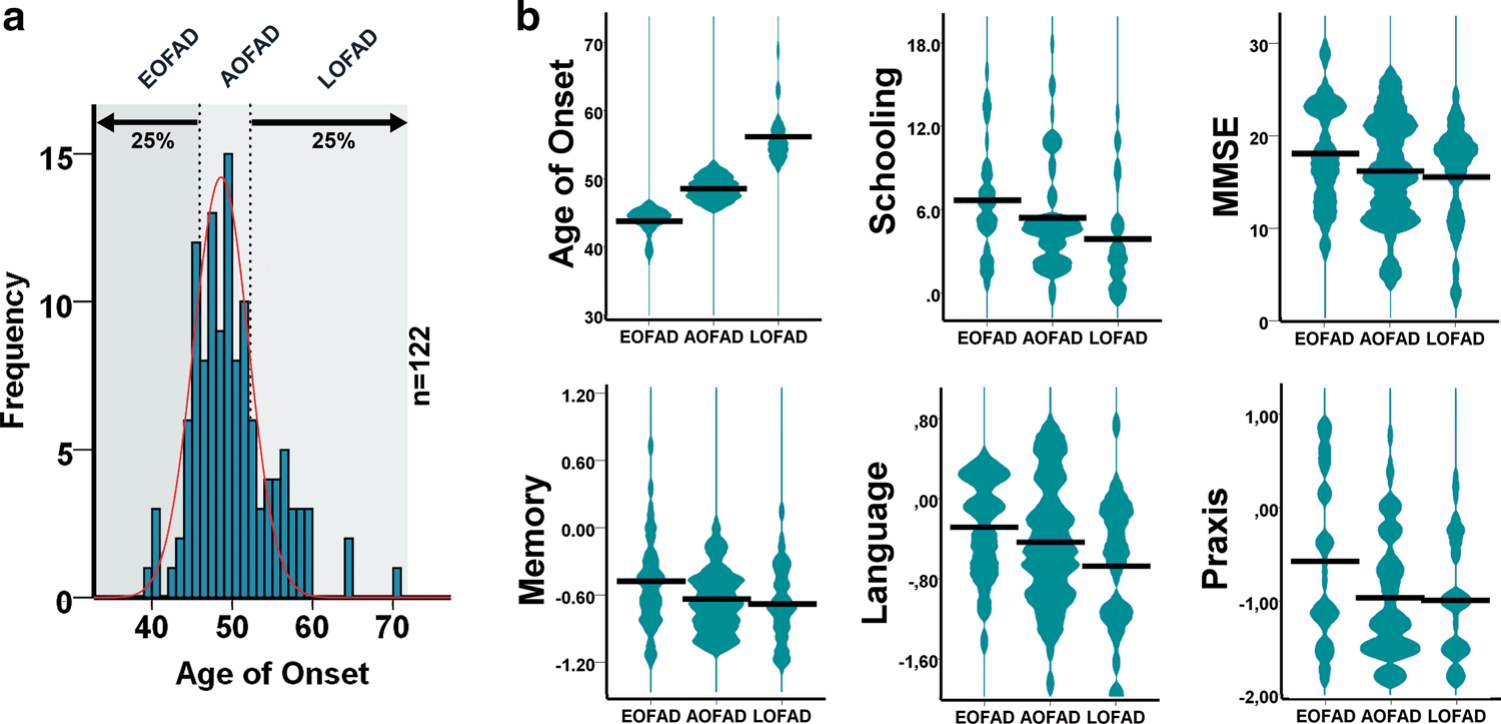
Distribution and characterization of ages of onset (AoO) of dementia on a sample of PSEN1 E280A FAD patients. A. Frequency histogram displaying the AoO distribution of a sample of PSEN1 E280A patients (*n* = 122). Plotted lines divide to the left the lower quartile (Early AoO = EOFAD, < 45 years old), two middle quartiles (Average AoO = AOFAD, 46–52 years old), and the upper quartile (Late AoO = LOFAD, > 53 years old). B. Violin plots for AoO, Schooling, MMSE scores and normalized Z scores for memory, language, and praxis cognitive domains. As expected, AoO showed significant differences between EOFAD, AOFAD, and LOFAD groups. From the other variables, schooling was significantly lower in LOFAD cases when compared with EOFAD (*p* = 0.018). Statistical differences in language and praxis domains are associated with schooling time (Supplementary Table 2, online resource)

The PSEN1 E280A population shows wide AoO variation ranging over 30 years [1]. Distribution of AoO in this population hints toward a bimodal distribution (Supplementary Fig. S1, online resource) [58]. Consequently, we grouped 122 demented carriers in AoO-quartiles. The early onset (EOFAD) quartile was between 39 and 46 years old (*n* = 33), and the two middle quartiles [average onset (AOFAD)] were concentrated between 47 and 52 years old (*n* = 61) and the late-onset (LOFAD) quartile stretched out between 53 and 70 years old (*n* = 28) (Fig. 1a, Table 1). Cognitive performance of these patients was evaluated at the time of disease onset and cognitive domains assessed as previously described [1]. Schooling time and Mini-Mental State Examination (MMSE) data were analyzed as baselines and LOFAD patients were significantly less educated than EOFAD patients. Memory, language and praxis domains were analyzed given their relevance in their phenotypic profile [46]. Statistically significant differences between AoO groups found in language and praxis domains pointing to lower performance with later onset disappeared when controlled for schooling. Finally, ApoE2 haplotype was overrepresented in later AoO groups (Supplementary Table 1–2, online resource), as previously described [58]. To assess molecular and pathological mechanisms influencing AoO in PSEN1 E280A, we studied 23 brains from PSEN1 E280A patients, grouped according to AoO as defined by the larger clinical sample, as Early, Average, and Late AoO (Table 1; Supplementary Table 3–5, online resource). These groups presented no significant differences in disease duration, ApoE haplotype, or clinical presentation. Age of death was significantly different among AoO groups as a direct consequence of similar disease duration. Also, the EOFAD group contained more females, but this difference was not statistically significant (Supplementary Table 4, online resource) and did not modify the clinical presentation in this group. As previously described [46], most of PSEN1 E280A patients presented with memory and language impairment at dementia onset; followed in later stages of the disease by parkinsonism, epileptic seizures, and myoclonus and abnormal gait. Other common clinical features at onset were behavioral changes and depression. Less common symptoms were cerebellar signs, headaches, and sleep disorders. Once we confirmed the clinical similarity between AoO groups, we followed with deep neuropathological phenotyping of these patients, specifically assessing Aβ and Tau pathophysiology together with genomic profiles.

**Table 1:** Demographic and cognitive profile of 122 PSEN1 E280A patients according to age of onset quartiles

Group	Patients	Gender (F)	ApoE2	ApoE3	ApoE4
Total	122	60.7%	3.3%	63.1%	23.8%
Q1: Early	33	72.7%	–	66.7%	24.2%
Q2–Q3: Average	61	57.4%	3.3%	63.9%	23.0%
Q4:Late	28	53.6%	7.1%	57.1%	25.0%
X Squared *p* value		0.237	0.31	0.886	0.982
			0.622		

Age of onset	p value vs				
	Mean	SD	Early	Average	Late
Early	44.21	1.95	–	*0.000*	*0.000*
Average	49.18	1.64	*0.000*	–	*0.000*
Late	56.96	3.79	*0.000*	*0.000*	–
Total	49.62	5.11	*0.000*		
Schooling					
Early	6.76	3.99	–	0.400	*0.018*
Average	5.49	3.88	0.400	–	0.261
Late	3.96	3.74	*0.018*	0.261	–
Total	5.48	3.97		*0.022*	
MMSE					
Early	19.06	5.48	–	0.384	0.26
Average	17.23	5.57	0.384	–	1.000
Late	16.61	5.50	0.260	1.000	–
Total	17.58	5.56		0.180	
Memory					
Early	− 0.47	0.45	–	0.094	0.097
Average	− 0.64	0.30	0.094	–	1.000
Late	− 0.67	0.36	0.097	1.000	–
Total	− 0.6	0.37		0.051	
Language					
Early	− 0.36	0.48	–	0.737	*0.037*
Average	− 0.5	0.60	0.737	–	0.245
Late	− 0.74	0.64	*0.037*	0.245	–
Total	− 0.52	0.59		*0.042*	
Praxis					
Early	− 0.6	0.87	–	*0.036*	0.08
Average	− 0.99	0.64	*0.036*	–	1.000
Late	− 1.01	0.62	0.080	1.000	–
Total	− 0.89	0.73		*0.026*	

### Aβ pathology profile according to AoO in PSEN1 E280A

**Fig. 2:**
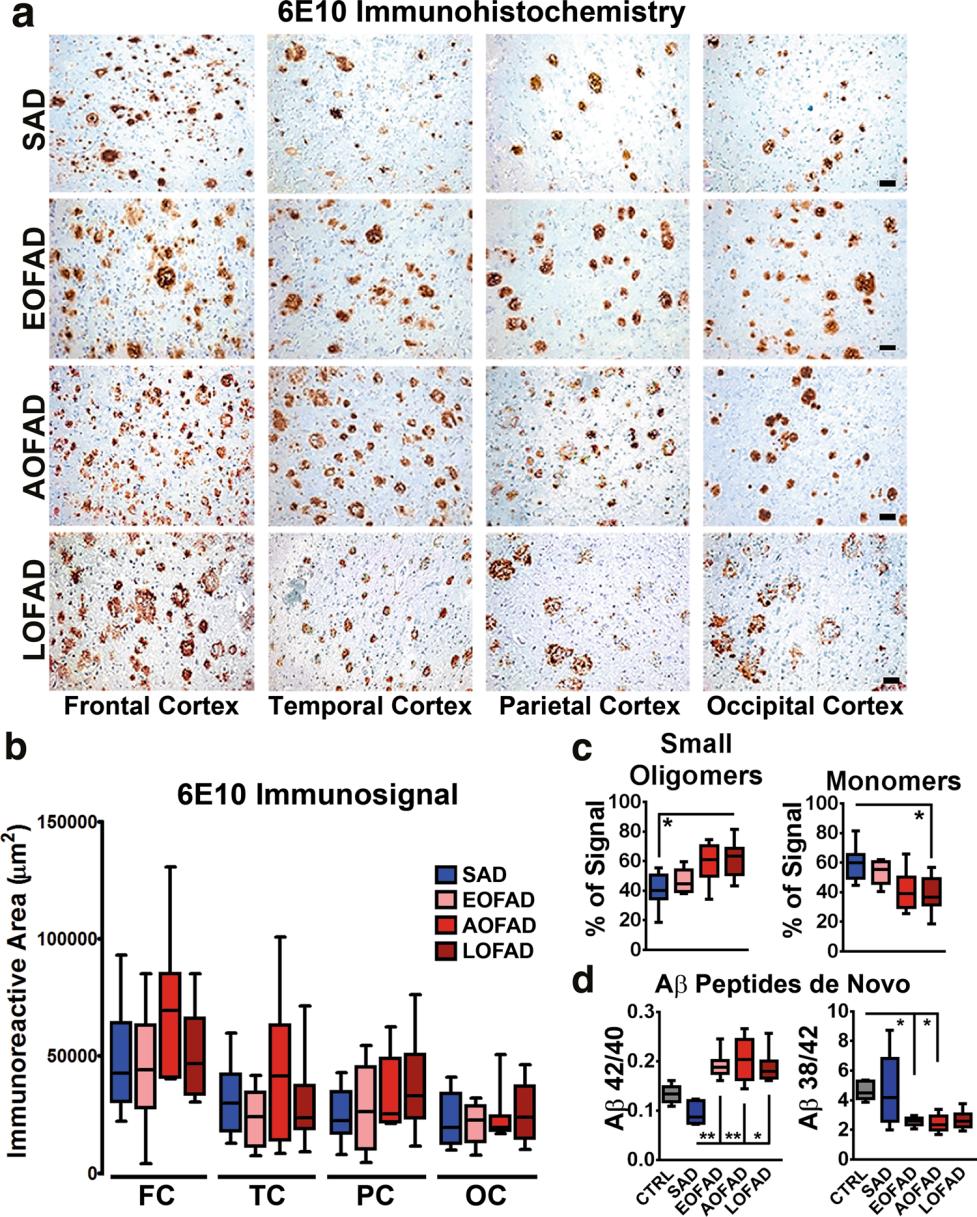
Distribution of Aβ pathology in brain regions of SAD and AoO groups of FAD PSEN1 E280A cases. **a** Immunohistochemical staining for Aβ using the 6E10 antibody in Frontal cortex, Temporal cortex, Parietal cortex, and Occipital cortex in SAD (*n* = 10), EOFAD (*n* = 8), AOFAD (*n* = 7), and LOFAD (*n* = 8). (Scale bar = 40 µm). **b** Quantification of Aβ plaque loads present in frontal cortex (FC), temporal cortex (TC), parietal cortex (PC), and occipital cortex (OC). There were not significant differences between groups. **c** Densitometric analysis boxplots for small Aβ oligomers and Aβ monomers. There were no significant differences between FAD groups. Only SAD cases presented significantly lower small oligomers and higher monomer levels when compared to LOFAD cases. **d** Boxplots of de novo generated Aβ peptide ratios in temporal cortex from Ctrl (*n* = 5), SAD (*n* = 5), EOFAD (*n* = 8), AOFAD (*n* = 7), and LOFAD (*n* = 8) cases. De novo generation of Aβ peptides was assessed by evaluating γ-secretase-dependent Aβ production in vitro using detergent-resistant membranes isolated from frontal cortex (see Supplementary Methods, online resource, for a detailed description). SAD Aβ1-42/1–40 ratios were significantly lower than EOFAD, AOFAD, and LOFAD; Ctrl Aβ1-38/1–42 ratios were significantly higher than EOFAD and AOFAD. (*= *p* ≤ 0.05, **= * p* ≤ 0.01)

PSEN1 E280A brains contain higher amounts of Aβ-42 plaques than SAD [33, 46, 49], and according to PET Florbetapir analysis, Aβ can be detected already at 25 years of age in asymptomatic PSEN1 E280A carriers [17]. Therefore, we assessed AoO-related changes in PSEN1 E280A patients (Fig. 2a). There were no differences between SAD and FAD patients, or within FAD groups concerning Aβ plaque-load or Aβ-42 plaque-load in the cerebral cortex (Fig. 2b; Supplementary Table 6, online resource). We also assessed Aβ-42 plaque-load in temporal cortex of SAD and FAD AoO groups. There were no differences within the FAD AoO groups nor between SAD and FAD (Supplementary Fig. 2a, b and Table 6, online resource). Although it has been suggested that Aβ oligomers rather than plaques are the main factor in Aβ-related pathogenicity, existing evidence regarding their possible role in AD is unclear [7]. However, there is an intrinsic difficulty in distinguishing between Aβ oligomers and APP fragments still containing the Aβ sequence [63]. For that, we analyzed TBS-soluble fractions from temporal cortex of SAD and FAD patients for differences in monomers and smaller sized Aβ oligomers (decamers and less) (Supplementary Fig. 2C , online resource). Oligomer formation and stability may be influenced by Aβ peptides composition [38]. Therefore, we also evaluated Aβ peptides profile in SAD and FAD groups using immunoprecipitation in combination with mass spectrometric analysis of frontal cortex (Supplementary Fig. 3A, online resource). There were no statistically significant differences between AoO FAD groups regarding these parameters. Only SAD showed significantly lower small Aβ oligomers levels when compared to LOFAD patients (Fig. 2c; Supplementary Fig. 2D and Table 6, online resource). Regarding Aβ peptides, SAD Aβ monomer levels and Aβ1-42 levels were significantly higher than LOFAD and AOFAD levels, respectively (Fig. 2c; Supplementary Fig. 3B, online resource). Studied peptides, plaques, and oligomers are the result of a long-term process which may mask differences in γ-secretase activity within different AoO FAD groups. We isolated detergent-resistant membranes containing functional γ-secretase complexes from frontal cortices of controls and FAD AoO groups to evaluate γ-secretase activity. As previously reported [23, 53], controls and SAD patients showed lower Aβ-42/1–40 ratio when compared to EOFAD and AOFAD cases, with SAD showing significantly lower ratios. Also, controls showed significantly higher Aβ-38/1–42 ratios when compared to EOFAD and AOFAD cases. There were no differences between AoO FAD groups (Fig. 2d; Supplementary Figs. 3C and Table 6, online resource). In summary, neither de novo generation of Aβ1-38, 1–40, and 1–42, Aβ peptide profiles nor Aβ aggregation as oligomers or plaques correlated with AoO in PSEN1 E280A FAD, suggesting a fully penetrant effect of PSEN1 E280A mutation in APP processing without influencing AoO.

**Fig. 3:**
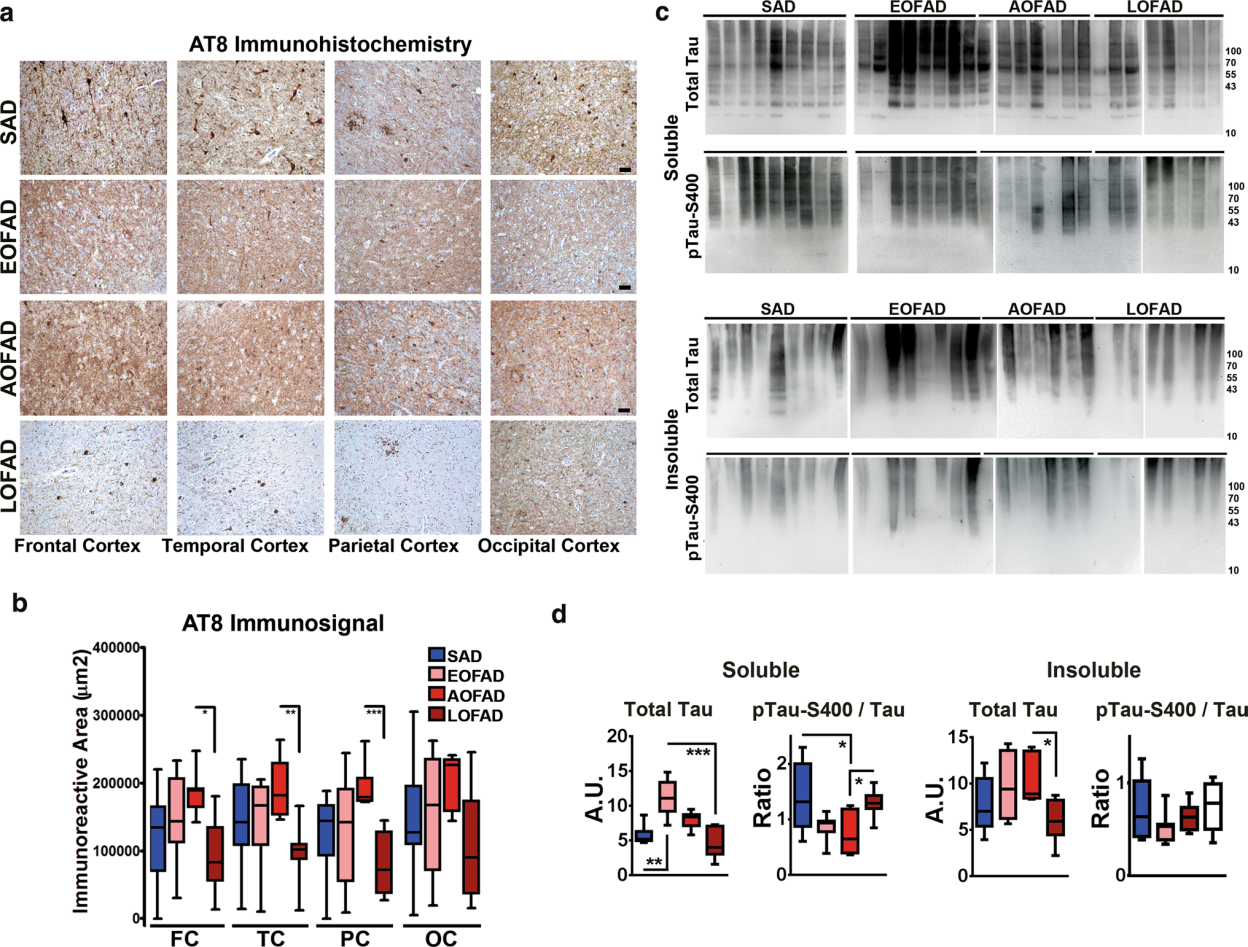
Distribution of pTau pathology in brain regions of SAD and AoO groups of FAD PSEN1 E280A cases. **a** Immunohistochemical staining for pTau using the AT8 antibody in frontal cortex, temporal cortex, parietal cortex, and occipital cortex from SAD (*n* = 10), EOFAD (*n* = 8), AOFAD (*n* = 7), and LOFAD (*n* = 8) cases. (Scale bar = 40 µm). **b** Quantification of pTau loads present in FC, TC, PC, OC, and CB. Only LOFAD pTau loads were significantly lower in FC, TC, and PC when compared to AOFAD. **c** Representative immunoblots of pTau aggregates patterns using total Tau and pTau-S400 antibodies in TBS-soluble (soluble fraction) and formic acid soluble (insoluble fraction) fractions from temporal cortex of SAD and AoO groups of PSEN1 E280A FAD cases. **d** Densitometric analysis boxplots for total Tau and pTau-S400 levels in soluble and insoluble fractions from temporal cortex from SAD and AoO groups of PSEN1 E280A FAD. In soluble fractions, EOFAD cases showed significantly higher total Tau levels when compared to SAD and LOFAD levels. Also, SAD and LOFAD showed significantly higher pTau-S400/Tau levels when compared with AOFAD. Regarding insoluble fractions, only AOFAD cases showed significantly higher total Tau levels than LOFAD cases (*= *p* ≤ 0.05, ** = * p* ≤ 0.01, *** = * p* ≤ 0.001)

### Decreased Tau pathology in late-onset PSEN1 E280A patients

AD features characteristic hyperphosphorylated Tau (pTau) deposits in dystrophic neurites surrounding Aβ-plaques and neuropil threads. Neurofibrillary tangles (NFTs), on the other hand, are also visible in other pathologies [22]. Regarding FAD, we previously showed that deposition of pTau in PSEN1 E280A FAD and SAD differs, suggesting a correlation between PSEN1 E280A FAD and specific hyperphosphorylated Tau aggregation patterns [47]. Thus, in the present study, we evaluated pTau pathology in cortices of SAD and PSEN1 E280A FAD patients (Fig. 3a). Surprisingly, we observed clear differences in pTau signal, with LOFAD patients showing lower signal in all cortices except occipital cortex, when compared to AOFAD patients (Fig. 3b; Supplementary Table 7, online resource). Moreover, LOFAD patients showed mostly NFTs with less neuropil threads and dystrophic neurites and absence of extracellular Tau fibrils at ultrastructural analysis. In contrast, EOFAD and AOFAD cases showed more neuropil threads and dystrophic neurites (Fig. 3a, Supplementary Figs. 4, 5, online resource). Histological differences between AoO FAD groups were present independently of disease duration, postmortem interval, or storage time of samples (Supplementary Fig. 4 , online resource). Furthermore, EOFAD patients showed significantly more total Tau in TBS-soluble fractions than SAD and LOFAD. Accordingly, LOFAD patients showed lower total Tau levels in formic acid soluble fractions than AOFAD patients (Fig. 3c; Supplementary Table 7, online resource). Interestingly, total Tau levels in TBS-soluble fractions correlate negatively with AoO in FAD (Supplementary Fig. 6 , online resource). Previously, we reported differential distribution of GSK3β and pTau pathology in PSEN1 E280A patients [47]. We evaluated pTau-S400 as an indirect marker of GSK3β activity in soluble and insoluble fractions from temporal cortex of SAD and FAD cases (Fig. 3c). pTau-S400/total Tau ratio showed that soluble fractions from AOFAD cases contained less pTau-S400 than those from SAD and LOFAD cases (Fig. 3d, Supplementary Table 7, online resource). It should be noted that as with Aβ, soluble pTau aggregates could be more deleterious than stable aggregates such as NFTs [31]. Tau-related pathology has been associated with synaptic loss in AD and it has been suggested that soluble non-aggregated pTau can be deleterious for the synapse [50]. We investigated synaptic density, represented by Synaptophysin positive particles, in clarified temporal cortices of FAD cases according to AoO. 99% of detected particles ranged between 0,139 and 10,926 µm^3^, indicating single and grouped puncta (Supplementary Figs. 7 and 8A, online resource). EOFAD cases showed significantly lower particle density (Supplementary Fig. 8B, online resource), while there were no differences in particle size between AoO FAD groups (Supplementary Fig. 8C, online resource). Synaptophysin-positive particle density correlated negatively with soluble pTau-S400 (Supplementary Fig. 8D, online resource) and disease duration (Supplementary Fig. 8E, online resource), suggesting an association between synaptic loss and disease duration that might be independent on AoO. To sum up, morphological and biochemical analysis show an increase in diffuse pTau in EOFAD and AOFAD patients, pointing to differences in Tau phosphorylation patterns and possible impact in synaptic loss. Also, morphological and biochemical analyses show an increase in neuropil threads and dystrophic neurites in EOFAD and AOFAD patients, pointing to differences in Tau phosphorylation patterns. Given that EOFAD and AOFAD groups did not show significant differences between them in our previous analysis, and considering that both groups together represent the lower half of AoO distribution in our sample (37–50 years old), we grouped them as a single group (E-AOFAD, Supplementary Table 8, online resource) for the following experiments (Table 2).

**Table 2:** Demographic and clinical profile of PSEN1 E280A patients used for deep phenotyping grouped according to age of onset

Group/Onset	Age of Onset	Age of Death	Disease Duration	Postmortem Index	Gender (F)	ApoE Haplotype (ApoE4)	Age of Onset, Affected Parent	Memory Impairment	Language Impairment	Parkinsonism	Seizures / Myoclonus	Abnormal gait	Behavioral changes	Depression	Cerebellar Signs	Headache	Sleep Disorder
EOFAD	37	47	10	138	F	NA	NA	+	+	+	+	+	+	-	+	+	+
	39	59	20	222	F	3/3	40	+	+	+	-	+			-		+
	40	59	19	360	F	3/3	51	+	+	+	+	+	+	+	-	+	-
	40	42	2	330	F	3/3	NA	+	+	+	+	-	-	+	+	-	-
	42	50	8	450	F	3/4	NA	+	+	+	-	-	+	+	+	+	-
	43	57	14	240	F	3/4	NA	+	+	+	+	+	+	+	-	-	-
	44	52	8	288	M	3/3	NA	+	+	+	+	+	-	-	+	-	-
	46	66	20	240	F	3/3	43	+	+	-	+	+			-		-
Mean (SD)	41.38 (2.93)	54.00 (7.71)	12.63 (6.67)	283.50 (95.99)	(87.5%)	(25%)		100%	100%	87.5%	75%	75%	50%	50%	50%	37.5%	25%
AOFAD	47*	54	7	330	F	3/3	57	+	+	+	+	+	-	+	+	+	-
	47*	56	9	198	M	3/3	57	+	+	+	+	+	+	+	+	-	+
	47	58	11	210	M	3/3	NA	+	+	+	+	+	+	+	-	+	-
	48	64	16	180	F	3/3	NA	+	+	+	+	+	-	-	+	-	-
	49	62	13	240	F	4/4	NA	+	+	+	+	-	+	+	+	-	+
	49**	55	6	168	M	3/3	NA	+	+	+	+	-	+	-	-	-	-
	50**	60	10	168	F	3/3	NA	+	+	+	+	+	+	+	-	-	-
Mean (SD)	48.14 (1.22)	58.43 (3.75)	10.29 (3.45)	213.43 (57.43)	(57.1%)	(14.3%)		100%	100%	100%	100%	71.4%	71.4%	71.4%	57.1%	28.6%	28.6%
LOFAD	52	68	16	384	F	3/3	NA	+	+	+	+	-	+	-	+	+	+
	53	60	7	240	M	3/4	NA	+	+	+	+	+	+	-	+	-	-
	54	63	9	330	M	3/3	NA	+	+	+	-	+	+	+	-	-	+
	55	64	9	180	F	NA	NA	+	+	+	+	+	-	+	-	+	+
	56	63	7	228	M	NA	NA	+	+	+	+	+	+	+	+	-	+
	58	71	13	300	F	3/3	NA	+	+	+	+	+	-	+	-	+	-
	58	70	12	192	F	3/3	NA	+	+	+	+	+	+	+	-	-	-
	62	74	12	330	F	3/3	NA	+	+	+	+	+	-	-	-	-	-
Mean (SD)	56.00 (3.25)	66.63 (4.84)	10.63 (3.16)	273.00 (73.55)	(62.5%)	(12.5%)		100%	100%	100%	87.5%	87.5%	62.5%	62.5%	37.5%	37.5%	50%
Total FAD	48.52 (6.74)	59.39 (8.15)	10.83 (4.85)	258.52 (80.64)	(56.5%)	(17.4%)		100%	100%	95.7%	87.0%	78.3%	60.9%	60.9%	47.8%	34.8%	34.8%

F = Female, M = Male, SD = Standard deviation, *=siblings, **=siblings

### Specific kinases profile associated with AoO in PSEN1 E280A cases

**Fig. 4:**
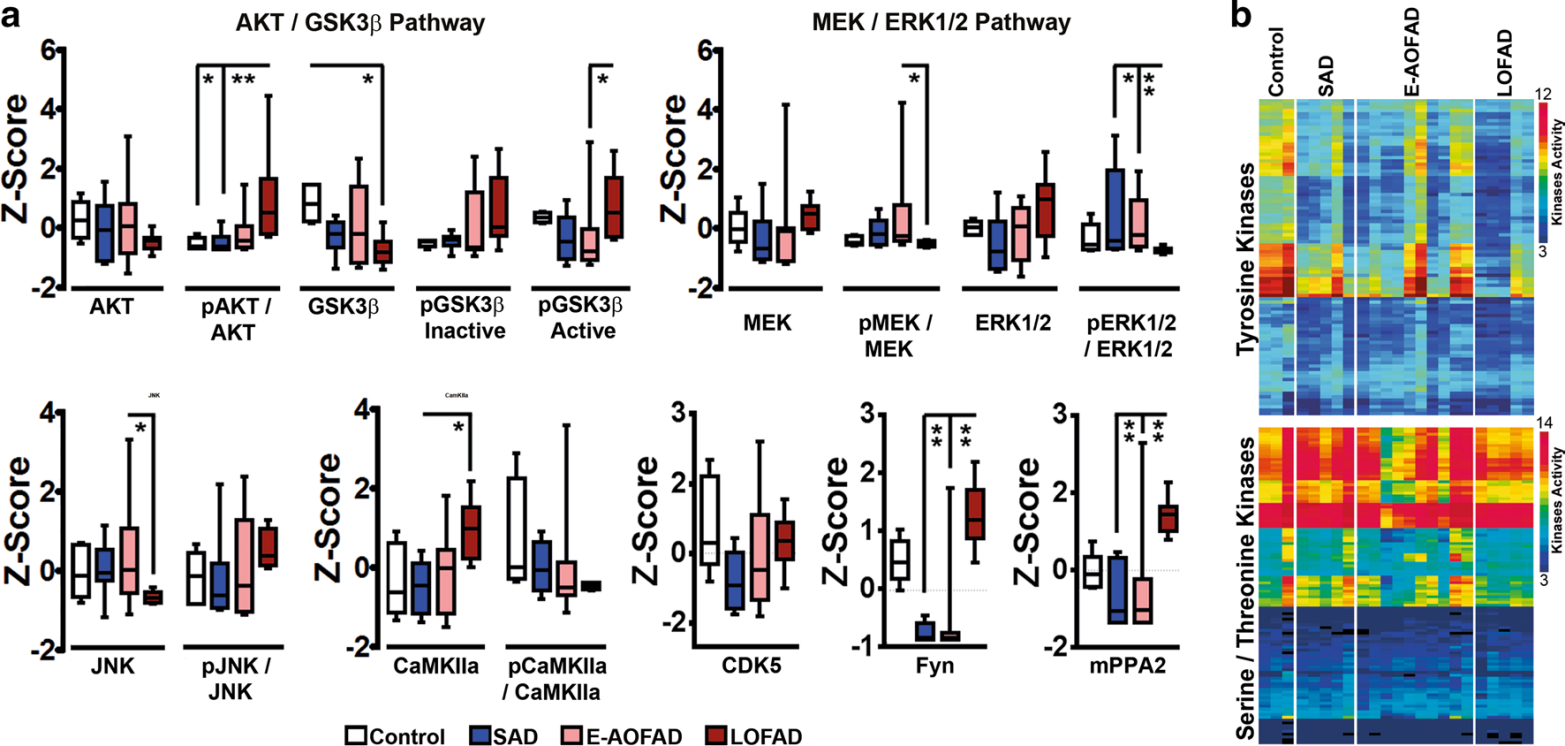
Tau kinase profile impact on AoO in PSEN1 E280A FAD. **a** Densitometric analysis in temporal cortex (TC) of basal and active forms of Tau kinases in controls (Control, *n* = 5), SAD (*n* = 10), E-AOFAD (*n* = 15), and LOFAD (*n* = 8). AKT/GSK3β pathway activation was significantly higher and MEK/ERK activation was lower in LOFAD when compared with E-AOFAD cases. Furthermore, JNK, Fyn, and mPPA2 steady-state levels were significantly different between LOFAD and E-AOFAD, while Fyn and mPPA2 levels were also significantly different between LOFAD and SAD cases (* = *p* ≤ 0.05, ** = * p* ≤ 0.01). **b** Logarithmic heatmap representation for significantly active or inactive kinases identified by microarray-based kinome profiling for Tyrosine (upper panel) and Serine/Threonine (lower panel) kinases in TC from Control (*n* = 3), SAD (*n* = 5), E-AOFAD (*n* = 10), and LOFAD (*n* = 5). LOFAD cases showed lower active Tyrosine kinases profile when compared with other groups. (FGFR4, RET, FGFR1, MERTK, EGFR, KIT, RYK, and other functionally related kinases; Supplementary Fig. 13A-B, online resource) and Serine/Threonine kinases (PDK1, MELK, MEK1, MEK2, MEK7, PKACb, PKCa, PKCb, PKCd, PKCe, PKCg, PKCt, HIPK2, HIPK3, HIPK4, AMPKa1, ANPa, and ZC2; Supplementary Fig. 14, online resource)

pTau aggregation and deposition is defined mostly by its phosphorylation pattern [60, 61]. We assessed steady-state levels and activation status of key kinases related with Tau phosphorylation AKT/GSK3β, MEK/ERK, CaMKIIa, JNK, CDK5, Fyn [60] and the phosphatase, mPPA2, in temporal cortex of patients and controls by western blotting (Supplementary Fig. 9, online resource). Significant differences were observed in LOFAD patients, including evidence of increased activation of the AKT/GSK3β pathway, lower activation of the MEK/ERK pathway, as well as increased total levels of JNK, CaMKIIa, Fyn, and mPPA2 when compared to E-AOFAD patients (Fig. 4a; Supplementary Fig. 9 and Table 9, online resource). GSK3β and ERK1/2 kinases phosphorylate Tau sequentially in different sites, requiring priming phosphorylation in serine 400 and 422, respectively [16, 34, 60]. Lower JNK levels and lower activation of the MEK/ERK pathway in LOFAD indicated decreased activation of stress associated pathways, while CaMKIIa, Fyn, and mPPA2 increased levels might reflect increased physiological kinase activation [16]. Taking these results together with pTau aggregation profiles, it becomes apparent that in E-AOFAD, more neuropil and soluble pTau associates with more active ERK1/2 and less-active GSK3β pathways leading to a specific Tau phosphorylation pattern. In effect, activated GSK3β correlates with activated ERK in SAD and E-AOFAD patients (Supplementary Fig. 10A-B, online resource) and activated ERK correlates with AoO in FAD (Supplementary Fig. 11, online resource). To assess if observed differential kinase activities represent general changes in the kinome, we performed microarray-based kinome profiling using 280 bait peptides in the temporal cortex from SAD and FAD groups (Supplementary Table 3, online resource) [43]. In general, LOFAD cases showed significantly lower activity for the entire subset of tyrosine kinases when compared to controls while no significant differences were observed in serine/threonine kinase activity (Fig. 4b; Supplementary Fig. 12, online resource). In addition, LOFAD cases showed differential activation for specific kinases belonging to both kinase subtypes (Supplementary Figs. 13–14, online resource). All these results indicate that specific Tau stress kinases and general kinome activity are lower in LOFAD cases. Therefore, kinome activity modifies Tau phosphorylation associated with AoO in PSEN1 E280A patients.

### Proteasome function and Tau degradation impact on AoO in PSEN1 E280A

**Fig. 5:**
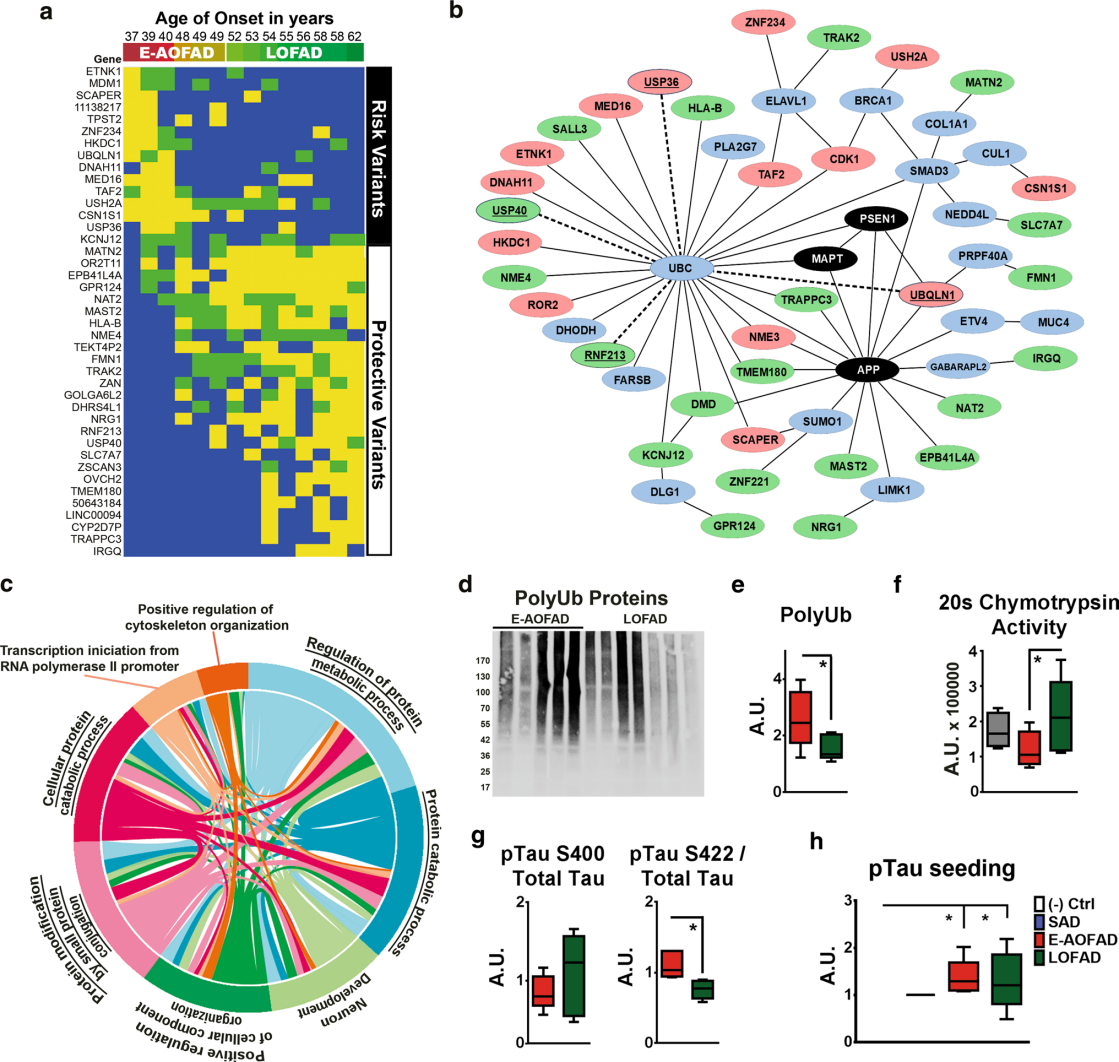
Association of ubiquitin and proteasome system function with pTau pathology and AoO in PSEN1 E280A FAD. **a** Schematic representation for AoO significantly associated variants in PSEN1 E280A FAD cases, early and average AoO FAD (E-AOFAD, *n* = 6), and LOFAD (*n* = 8), as characterized by whole-exome capture. Yellow = identified uncommon variants were present in both alleles, Gree* n* = identified uncommon variants were present in only one allele and, Blue = identified common variants were present in both alleles. **b** Protein network analysis of main AD-related genes and genes showing association with AoO in evaluated PSEN1 E280A FAD cases. Black ovals depict AD-related genes, pink circles early AoO-associated genes, and green circles late AoO-associated genes. Genes directly involved in the ubiquitin–proteasome system are underscored and connected with dotted lines. **c** Chord diagram of statistically significant biological pathways (GO:BP analysis) in which AoO-associated genes take part. Four pathways, underscored, are related with protein metabolism and catabolism. **d** Representative semi-denaturing immunoblot for polyubiquitinated proteins (PolyUb) in TBS-soluble fractions from temporal cortex of early and average AoO FAD. E. Densitometric analysis of PolyUb in TBS-soluble fractions from temporal cortex of E-AOFAD (*n* = 5) and LOFAD (*n* = 7). E-AOFAD cases showed significantly higher levels of PolyUb. F. Chymotrypsin S20 proteasome activity in temporal cortex tissue from controls (*n* = 4), E-AOFAD (*n* = 6), and LOFAD (*n* = 8) cases as detected by LLVY-AMC substrate. LOFAD cases showed significantly higher Chymotrypsine proteasome activity when compared to E-AOFAD cases. **g** Densitometric analysis of co-immunoprecipitation using monoclonal polyubiquitin antibody as bait and immunoblots for total Tau, pTau-S400, and pTau-S422 in TBS-soluble fractions from temporal cortex of E-AOFAD (*n* = 5) and LOFAD (*n* = 7). There were no significant differences between E-AOFAD and LOFAD cases for pTau-S400/total Tau ratio. However, E-AOFAD cases showed higher pTau-S422/total Tau ratio than LOFAD cases. **h** Bar graph representing pTau seeding capacity assays in temporal cortices homogenates from negative Controls (*n* = 2), SAD (*n* = 2), E-AOFAD (*n* = 5), and LOFAD (*n* = 5) cases. Both FAD groups showed increased pTau seeding activity when compared to negative controls. (* = *p* ≤ 0.05)

Previously, we have reported several gene variants that are associated with AoO in PSEN1 E280A, yet none of them were directly associated with Aβ or Tau pathology [28, 29, 57–59]. It is possible that other factors apart from PS1/γ-secretase dysfunction indirectly influence Tau pathology and act as AoO modifiers in this population. Thus, we selected the six E-AOFAD patients with earlier AoO and compared them with all LOFAD patients (Supplementary Table 3, online resource) using Whole Exome Capture (WEC) in DNA extracted from brain tissue. We were able to identify 60 genetic variants including common, uncommon, and novel variants significantly associated with AoO in these patients (Supplementary Tables 10–12, online resource). Accordingly, these variants confer risk or protection for early AoO in PSEN1 E280A FAD (Fig. 5a; Supplementary Fig. 15, online resource). None of the genes identified in this study have previously been reported to influence AoO in this population [28, 29, 57–59] and only one gene, UBQL1, associates with risk for SAD [24, 65]. These findings suggest that the accumulated effect of diverse genetic variants can have an impact in the neurodegenerative profile of PSEN1 E280A FAD modifying AoO. Interactome analysis of these genes together with main AD risk genes showed interaction with ubiquitin by first or second degree (Fig. 5b; Supplementary Table 13, online resource). Furthermore, GO biological pathway analysis identified nine significantly enriched pathways involving proteins located in our network analysis. Four out of those pathways are directly involved with protein catabolism or metabolism (Fig. 5c; Supplementary Table 14, online resource). In conjunction with the ubiquitin role as a hub in our network, these findings suggest protein degradation as a main modifier of AoO in PSEN1E280A patients. Using temporal cortices of FAD patients, we analyzed levels polyubiquitinated (polyUb) proteins and polyUb Tau profiles in TBS-soluble fractions, together with proteasomal S20 chymotrypsin activity. As expected, LOFAD patients showed significantly lower levels of polyUb signal (Fig. 5d, e; Supplementary Table 15, online resource), higher proteasomal activity (Fig. 5f, Supplementary Table 15, online resource), and lower pTau-S422/total Tau immunoprecipitated with polyUb antibody (Fig. 5g; Supplementary Figs. 16, 17A-B and Supplementary Table 15, online resource). PolyUb signal correlates with S20 chymotrypsin activity and GSK3β activation in PSEN1 E280A FAD patients (Supplementary Fig. 18A-B, online resource). Furthermore, pTau-S422/total Tau ratios correlate negatively with AoO and activated GSK3β, and positively with activated ERK1/2 in PSEN1 E280A FAD (Supplementary Fig. 19, online resource).

### Role of tau seeding in variance in AoO

Finally, recent findings indicate a role for pTau seeding in AD [12]. It is possible that the degree of pTau pathology variance in the FAD AoO groups is related with pTau seeding activity [15]. We tested this in frontal and temporal cortices of E-AOFAD and LOFAD cases. FAD groups showed increased pTau seeding activity compared to negative controls in both frontal and temporal cortices (Fig. 5h; Supplementary Fig. 20, online resource). E-AOFAD cases showed increased pTau seeding activity compared to positive (SAD) controls in frontal cortex. Regarding AoO groups, E-AOFAD cases pTau seeding was significantly higher than LOFAD cases in frontal cortex (Supplementary Fig. 20, online resource). All these findings taken together suggest a correlation between protein degradation and pTau pathology in these patients.

## Discussion

**Fig. 6:**
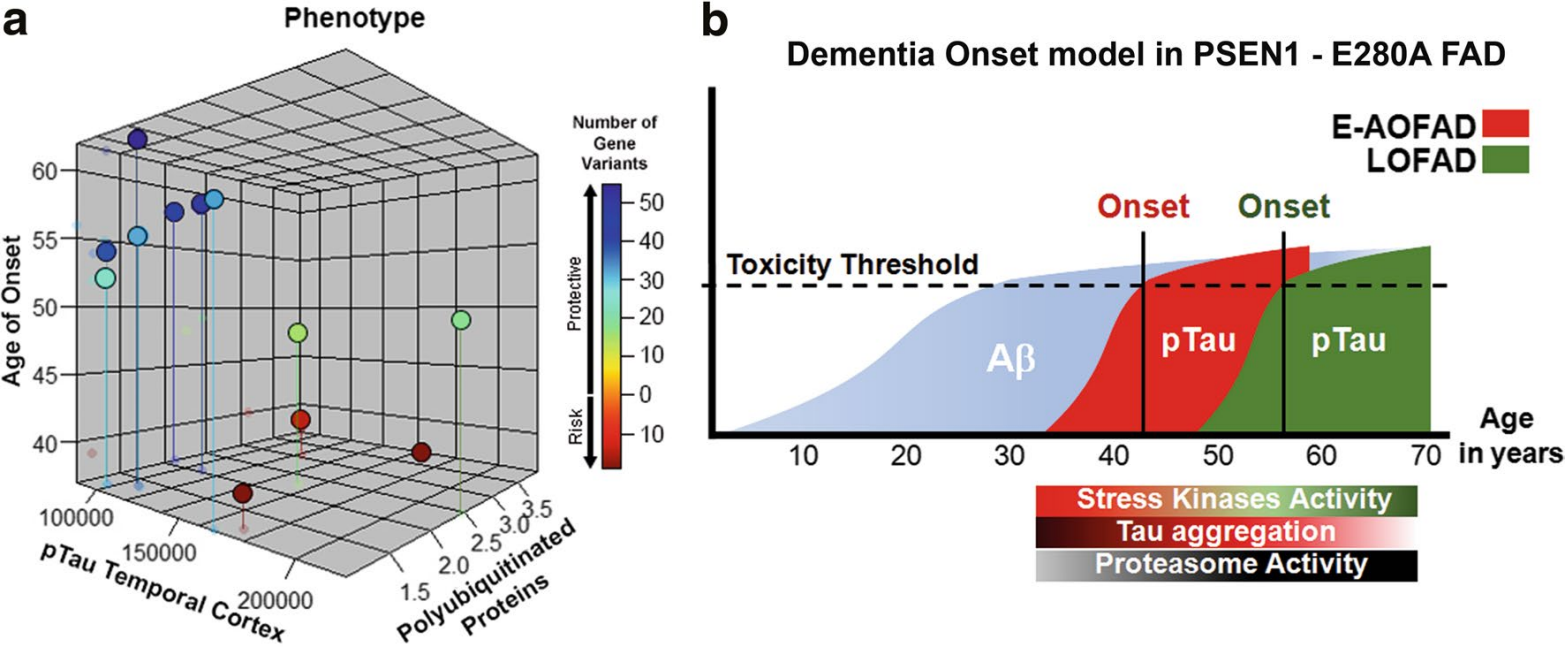
Phenotypic and dementia onset models in PSEN1-E280A FAD. **a** Three-dimensional plot of E-OFAD (*n* = 5) and LOFAD (*n* = 7) cases according to pTau levels in temporal cortex (Fig. 3), PolyUb and AoO. Colors from blue to red represent number of protective or risk genetic variants identified in these patients. LOFAD cases cluster together with lower pTau levels in temporal cortex, low polyubiquitinated protein levels, and higher number of protective gene variants. **b** Schematic model for the impact of Aβ aggregation, pTau aggregation, and ubiquitin/proteasome function in dementia onset in PSEN1 E280A FAD. All patients have in common Aβ progression rate. However, earlier disease onset is associated with earlier and more severe Tau pathology, stress kinases activation, and lower proteasome activity

In the present study, we show that Aβ production and deposition do not explain heterogeneity of AoO in PSEN1 E280A patients, whereas Tau phosphorylation and degradation do. Patients with late AoO show less pTau pathology associated with differences in the post-translational modifications of Tau, the “bioactivity” or seeding potential of it as well as with genetic variants affecting the proteasome system (Fig. 6a). Interestingly, differences in AoO do not associate with differences in duration, severity, or clinical presentation of the disease as previously suggested [21].

FAD has been reported to display more Aβ aggregates than SAD [30]. Furthermore, mutated PS1 affects the processivity of the γ-cleavage of APP, favoring production of longer Aβ peptides ending at amino acid 42 or 43 (1–42 and 1–43) over short Aβ1-40 and Aβ1-38 [52]. We have previously evaluated de novo Aβ peptide production from APP C99 in brain tissue from FAD patients showing altered Aβ1-42/1–40–Aβ1-38/1–42 ratios when compared with healthy controls and SAD patients, showing evidence of impaired γ-secretase carboxypeptidase-like efficiency in FAD [53]. We have also shown distinctive Aβ1-38 and Aβ1-42 aggregation patterns in FAD patients when compared with SAD patients [13]. In this study, biochemical differences in Aβ peptide profiles of PSEN1 E280A FAD patients did not correlate with AoO. Meanwhile, SAD showed lower levels of small Aβ oligomers and lower de novo generation of Aβ1-42/1–40 peptides, consistent with the previous findings [53]. The amyloid hypothesis is widely supported and has contributed with valuable insights regarding mechanisms of neurodegeneration in AD, yet it does not fully explain disease presentation [5]. For instance, severity of Aβ pathology does not correlate with cognitive impairment and dementia [20].

Recent studies in SAD suggest that tau is heterogeneously modified, with various phosphorylation, ubiquitinylation, and acetylation sites altered in individual patients [62]. Moreover, differential phosphorylation of high molecular weight soluble tau species correlated with seeding activity in a bioassay, AoO, and rate of progression in SAD [15]. These data are reminiscent of our current observations in FAD alterations in tau post-translational modifications, including patterns of phosphorylation, and potentially extent of ubiquitinylation, correlate with AoO even in this unique cohort with a strong autosomal-dominant driver of early onset AD. Sequential and combined phosphorylation of Tau requires the activation of a distinct set of kinases, some of them interconnected by regulatory pathways [34, 60]. For instance, in AD and other dementias, inactivation of GSK3β together with activation of stress kinases ERK1/2 or JNK, play a key role in pTau aggregation pattern [8, 16]. Moreover, kinome analysis has shown alternative regulatory pathways for Tau phosphorylation [6] with specific sets of kinases influencing Tau aggregation [56]. Since PSEN1 E280A LOFAD patients show less pTau, together with specific kinases and kinome profiles, a link between dementia onset and pTau pathology can be suggested. Accordingly, in contrast to Aβ, deposition of pTau correlates with severity and cognitive dysfunction in AD better than Aβ plaque loads [37, 42] and has recently become a key target in AD drug development [18]. All our findings are in line with a multifactorial neurodegenerative process in AD [51]. Hence, it is possible that other factors apart from PS1/ γ-secretase dysfunction influence Tau pathology and act as AoO modifiers. We found association of AoO with genetic variants in genes involved in protein degradation. These findings are in accordance with a crucial role of ubiquitin-mediated protein degradation in neurodegeneration [11]. pTau ubiquitination is decisive for its aggregation, degradation, and deposition [54]. In addition, it has been shown that Tau ubiquitination modulates activation of stress kinases potentially increasing pTau pathology [2]. We conclude that, while Tau kinases and general kinome activity affect the buildup of pTau aggregates, genetic variants may also contribute to modifying proteasomal activity and protein catabolism, thereby affecting pTau degradation with both mechanisms influencing AoO of dementia in PSEN1 E280A FAD. One consequence of these changes in post-translational modifications appears to be a difference in seeding bioactivity of tau, as has been reported in SAD, as well [15].

Our results suggest that while γ-secretase dysfunction is the first step toward neurodegeneration in PSEN1 E280A FAD patients, it is one among several pathological events in their neurodegenerative process. PS1 is widely expressed in the nervous system [32] and has a role in neurodevelopment [26]. Therefore, effects of PSEN1 E280A mutation on γ-secretase and other PS1 functions with abnormal Aβ ratios and aggregation of longer Aβ species are  occurring from birth onwards. In fact, asymptomatic PSEN1 E280A carriers show abnormal Aβ1-42 plasma levels in their teenage years [40] and accumulate Aβ in their brains as early as 28 years of age, with Aβ levels reaching a plateau in their thirties [17]. Recently, Tau deposition was detected in PSEN1 E280A carriers older than 38 years using flortaucipir F 18 PET imaging (FTP). In fact, only 3 out of 12 PSEN1 E280A carriers were older than 40 years of age and presented with cognitive impairment. FTP levels correlated with cognitive impairment, showed faster aggregation kinetics and its binding was observed only in PSEN1 E280A carriers that already show substantial Aβ deposition levels in cortical regions [41]. This observation provides a snapshot of the sequence of events regarding Aβ and Tau pathology in this population. More importantly, a recent case report of a PSEN1 E280A patient, also carrying the ApoE Christchurch (R136S) mutation, showed three decades of delayed onset of cognitive impairment together with high Aβ and low Tau pathology as determined with PET, supporting our hypothesis of pTau pathology as the main determinant factor in AoO in FAD [3]. Taking into consideration Aβ and Tau PET findings together with our data, we propose a neurodegeneration model for PSEN1 E280A carriers where disturbed Aβ production and accumulation are the prerequisite for developing dementia, while other factors, such as altered protein degradation affecting Tau phosphorylation, are important modifiers accelerating disease kinetics and cognitive decline. Conversely, those with protective factors for these processes will develop dementia later (Fig. 6b and reference 3). Currently, there is an ongoing clinical trial involving PSEN1 E280A carriers (NCT01998841), which are treated with Crenezumab, a humanized anti-Aβ antibody [45]. Our results suggest that delay of AoO alone may not be an optimal indicator of successful therapeutic response in this population without a genetic screening for known [28, 29, 57–59] and novel variants affecting AoO such as the ones reported here. Besides, it remains to be established how treated mutation carriers will respond after successful removal of Aβ.

The main limitation of our study is its descriptive nature. Our results indicate that even in this genetically uniform population carrying an identical PSEN1 mutation, additional individual genetic footprints or differences in activity of a suite of kinases and of the protein degradation machinery in neurons act as modifiers of AD pathology generating high inter-individual heterogeneity. Among FAD cohorts, PSEN1 E280A is the only one large enough to provide an adequate sample size of patients to analyze AoO with a common background. To collect an equivalent sample size with other PSEN1 cohorts, one should group together different PSEN1 mutations from different ethnic and geographic origins, with the corresponding genetic heterogeneity, introducing a systematic error for this kind of analysis. Therefore, only significantly larger, multicenter studies, using deep phenotyping among FAD and SAD cohorts offer the possibility to obtain further insights into disease severity modifiers and interdependent mechanisms of neurodegeneration in AD.

Our finding that generation and deposition of Aβ do not determine disease severity, whereas pTau deposition and functionality of the proteasome system do, has direct implications for SAD. A growing body of evidence shows that pTau pathology is the main determining factor for disease severity in SAD [15, 36]. Considering that SAD does not share the genetic uniformity and etiology found in the PSEN1 E280A population, an even wider pathological diversity can be assumed and should be considered when devising treatment strategies. Therefore, treatments oriented to stop Aβ aggregation or enhance its clearance might delay but not preclude cognitive impairment and neurodegeneration in susceptible individuals. Disease heterogeneity in AD reflects its multifactorial nature. Our findings, together with those reported for SAD, suggest that a personalized medicine approach might be the best strategy to follow in AD treatment.

## Supplementary Information

Below is the link to the electronic supplementary material.

Supplementary file1 (PDF 4871 KB)
