# Supplementary material for: A multifactorial model of pathology for age of onset heterogeneity in familial Alzheimer’s disease
Source: Acta Neuropathol. 2020 Dec 14;141(2):217–33. doi: 10.1007/s00401-020-02249-0 (PMC7847436; doi:10.1007/s00401-020-02249-0)
Supplement: Supplementary file 1 — Supplementary file1 (PDF 4871 KB) [file 401_2020_2249_MOESM1_ESM.pdf]

Supplementary Materials for:

A multifactorial model of pathology for age of onset variability in Familial Alzheimer's disease

Diego Sepulveda-Falla[1,2*]; Lucia Chavez-Gutierrez[3,4]; Erik Portelius[5,6]; Jorge I Vélez[7,8]; Simon Dujardin[9]; Alvaro Barrera-Ocampo[1,10]; Felix Dinkel[1]; Christian Hagel[1]; Berta Puig[1]; Claudio Mastronardi[7,11]; Francisco Lopera[2]; Bradley T. Hyman[9]; Kaj Blennow[5,6]; Mauricio Arcos-Burgos[11]; Bart de Strooper[3,4,12]; Markus Glatzel[1*]

Affiliations:

[1] Institute of Neuropathology, University Medical Center Hamburg-Eppendorf, Hamburg, Germany.

[2] Neuroscience Group of Antioquia, Faculty of Medicine, University of Antioquia, Medellín, Colombia.

[3] VIB center for Brain and disease Research, 3000 Leuven, Belgium.

[4] Department of Neurology, KU Leuven, Belgium.

[5] Institute of Neuroscience and Physiology, Dept. of Psychiatry and Neurochemistry. The Sahlgrenska Academy at the University of Gothenburg, Mölndal, Sweden.

[6] Clinical Neurochemistry Laboratory, Sahlgrenska University Hospital, Mölndal SE-431 80 Mölndal, Sweden.

[7] Department of Genome Sciences, John Curtin School of Medical Research, Australian National University, Canberra, ACT, Australia.

[8] Universidad del Norte, Barranquilla, Colombia.

[9] Department of Neurology, Massachusetts General Hospital, Harvard Medical School, MassGeneral Institute for Neurodegenerative Disease, Charlestown, USA.

[10] Universidad Icesi, Facultad de Ciencias Naturales, Departamento de Ciencias Farmaceuticas, Grupo Natura, Calle 18 No. 122 -135, Cali, Colombia

[11] GIPSI group, Department of Psychiatry, Medical Research Institute, University of Antioquia, Medellín, Colombia.

[12] UK Dementia Research Institute, University College London, Queen Square, WC1N 3BG London, UK.

*Correspondence to: Diego Sepulveda-Falla (dsepulve@uke.de) and Markus Glatzel (m.glatzel@uke.de).

Index

Item	Page
Extended Methods	4
Tables	11
Table 1. Demographic and cognitive performance according to age of onset in 122 PSEN1 E280A patients	11
Table 2. Logistic Regression of Age of Onset and Schooling time as predictors of cognitive domains scores in PSEN1 E280A Age of Onset subgroups	12
Table 3. Cases used for genetic, biochemical and pathological studies	13
Table 4. Demographic and clinical characterization of PSEN1 E280A patients used for biochemical and pathological studies	14
Table 5. Demographic and clinical characterization of PSEN1 E280A patients used for biochemical and pathological studies (cont.)	15
Table 6. Aβ pathology according Age of Onset in PSEN1 E280A FAD	16
Table 7. pTau pathology according Age of Onset in PSEN1 E280A FAD	19
Table 8. pTau pathology comparison E-AOFAD vs LOSAD in PSEN1 E280A FAD	21
Table 9. pTau-related Kinases levels according Age of Onset in PSEN1 E280A FAD	22
Table 10. Novel significant uncommon variants after FDR and Major Haplotype analysis	26
Table 11. Top 20 Known uncommon variants, functional mutations	27
Table 12. Top 20 common variants, functional mutations	28
Table 13. Protein-protein interactions of identified genes	29
Table 14. Significant biological pathways from protein network	31
Table 15. Ubiquitination, polyubiquitination and polyubiquitinated pTau according Age of Onset in PSEN1 E280A FAD	32
Table 16. Primary antibodies used in the study	33
Table 17. Secondary antibodies used in the study	33
Figures	34

Figure 1. AoO distribution in PSEN1 E280A 34

Figure 2. Aβ pathology in AD cases 35

Figure 3. Aβ peptides and enzymatic turnover of gamma secretase of SAD and FAD cases 36

Figure 4. pTau pathology according to disease duration and Age of Onset in PSEN1 E280A FAD patients 37

Figure 5. Ultrastructural analysis of temporal cortex from the SAD and AoO groups of PSEN1 E280A 38

Figure 6. Correlation analysis between levels of total Tau in TBS soluble fractions and AOO in PSEN1 FAD patients 39

Figure 7. Representative 3D rendering of clarified formalin fixed temporal cortices from PS1E280A FAD cases stained for synaptophysin and grouped by age of onset. 40

Figure 8. Synaptophysin particle distribution according to size in PS1 E280A FAD cases grouped by age of onset. 41

Figure 9. Representative blots of studied pTau-related kinases in Temporal cortex of controls, SAD and FAD cases 42

Figure 10. Correlation of normalized pERK1/2 / ERK1/2 ratio normalized GSK3β Y216 / GSK3β levels 43

Figure 11. Correlation of normalized pERK1/2 / ERK1/2 ratio and age of onset 44

Figure 12. Significantly different active Tyrosin and Serine/Threonine kinases between FAD cases and controls 45

Figure 13. Upstream kinase analysis of Tyrosine Kinases Ctrl vs LOFAD cases and dendritic tree of significantly specific kinases 46

Figure 14. Upstream kinase analysis of Serine / Threonine Kinases Ctrl vs LOFAD cases and dendritic tree of significantly specific kinases 47

Figure 15. Demographic characteristics and distribution of risk / protective genetic variants according to age of onset in studied PSEN1 E280A cases 48

Figure 16. Proof of principle for co-immunoprecipitation with anti-polyubiquitinated and anti-Tau antibodies in brain tissue 49

Figure 17. Co-immunoprecipitation using monoclonal polyubiquitin antibody as bait and immunoblots for total Tau, pTau-S400 and pTau-S422 50

Figure 18. Correlation of polyubiquitinated proteins with S20 Chimotrypsin activity and normalized GSK3b Y216 / GSK3b levels 51

Figure 19. Correlation of immunoprecipitatedpTau S422/Tau with AoO, normalized pERK1/2 / ERK1/2 ratio and normalized GSK3b Y216 / GSK3b levels 52

Figure 20. pTau seeding activity in frontal cortex. 53

Extended Methods

Patients and clinical data collection

Descendants of patients with confirmed PSEN1 E280A mutations were enrolled into the E280A Antioquia cohort study, an ongoing work at the University of Antioquia, Colombia. Participants were included if they were aged over 17 years. There were no other exclusion criteria for medical and neuropsychological monitoring. All participants (both carriers and non-carriers of PSEN1 E280A) or their guardians provided written informed consent for participation in the study; if the physician thought that a participant had dementia, their guardian provided written informed consent. All assessed participants with no evident dementia and examiners were masked to genetic status throughout monitoring. For genetic analyses, genomic DNA was extracted from blood by standard protocols, and PSEN1 E280A characterization was done as previously described. 19 Genomic DNA was amplified with the primers PSEN1-S 5′ AACAGCTCAGGAGAGGAATG 3′ and PSEN1-AS 5′ GATGAGACAAGTNCCNTGAA 3′. We used the restriction enzyme BsmI for restriction fragment length polymorphism analysis. The study was approved by the medical ethics board of the University of Antioquia. Follow up was conducted as previously described (1). Briefly, follow-up examinations included medical and neuropsychological assessments, which focused on registration of memory complaints and general cognitive function. Neuropsychological tests were performed by neuropsychologically trained personnel. Medical history and neuropsychological assessments were stored at the systematized information system for the neuroscience group of Antioquia (SISNE).

For cognitive assessment of differences between ages of onset we used a protocol including the CERAD (consortium to establish a registry for Alzheimer's disease) neuropsychological test battery with additional neuropsychological tests, as previously described (1). Basic demographic information was collected, including schooling time. Minimental (MMSE) testing served as baseline examination. Tests were applied according to studied cognitive domain as follows. For memory assessments, we used the memory of three phrases test, Rey-Osterrieth complex figure test (recall), list of words tests (total corrects, total intrusions, recall, intrusions recall, recognition “yes”, and recognition “no”), and recall of line drawings test. We assessed language ability with the verbal fluency and naming test. To assess constructional praxis, we used the constructional praxis test and the Rey-Osterrieth complex figure test (copy). This expanded CERAD neuropsychological protocol has been validated for the Colombian population in participants over 50 years of age and it has been also established for normal parameters for participants under 50 years of age (1).

Furthermore, for neuropathological studies, Alzheimer’s disease brain samples were collected from the Brain tissue bank from the University of Antioquia. PSEN1E280A FAD cases and sporadic cases or their families, signed informed consent for post-mortem brain donation and tissue use in scientific studies. All procedures were performed following ethical board approval from the University. Sporadic cases were selected based on clinical diagnosis of probable AD, lack of family history of dementia and tested as non-carriers for PSEN1 E280A mutation. Control cases were collected in the brain bank of the Bellvitge Hospital, Barcelona, Spain. They were selected based on lack of brain trauma, cognitive or neurological symptoms before death.

Morphological methods

Histopathological methods. All morphological analyses were performed on 3 μm thick de-paraffinized sections from cortices of SAD and PSEN1 E280A FAD cases (Table 3). Immunohistochemical stainings were performed following pre-treatment for antigen retrieval and probed with monoclonal anti-Aβ antibody, anti pTau antibody and anti-Aβ 1-42 antibody (Table 16). All immunohistochemical stainings were performed on an automated Ventana HX system (Ventana-Roche Medical systems, Tucson, AZ, USA) following the manufacturer’s instructions. Experimental groups were stained in one run for each antibody to provide uniform staining conditions. Primary antibodies were visualized using a standard diaminobenzidine streptavidin-biotin horseradish peroxidase method (Sigma Aldrich, Hamburg, Germany). For quantification of primary antibodies

immunosignal, three representative regions (0,1349 mm2 each) were analyzed by quantifying the area immunoreactive for each antigen using the AxioVision 4.6 software (Carl Zeiss, Oberkochen, Germany) according to published methods (2).

Ultrastructural analysis. Ultrastructural analysis was performed using glutaraldehyde-fixed brain tissue from SAD, EOFAD, AOFAD and LOFAD patients as previously described (2). Temporal cortex samples were excised from paraformaldehyde fixed tissue after localizing specific areas of extracellular pTau deposits or an equivalent area from LOFAD cases. Samples were fixed with glutaraldehyde and chrome-osmium, dehydrated in ethanol, and embedded in Epon 812 (Serva Electrophoresis GmbH). After polymerization, 1-µm-thick sections were cut, stained with toluidine blue, and checked for presence of amyloid plaques. To further process them for electron microscopy, relevant specimens were cut into 60- to 80-nm-thick sections, which were contrasted with uranyl acetate and lead solution. Sections were viewed under a LEO EM 912AB electron microscope (Zeiss).

Tissue clarification and imaging. Formalin fixed 1 cm length x 1 cm width x 500 µm thick, temporal cortex samples from 5 EOFAD, 5 AOFAD and 5 LOFAD cases were clarified using a CLARITY protocol as previously described (17). Briefly, samples were submerged in Hydrogel monomer solution (Paraformaldehyde 4%, Acrylamide/Bisacrylamide (40%) 19:1 2%/0.05% and VA-044 0.25%) during 7 days at 4ºC. Tissue was transferred the tissue to a 50 ml tube in hydrogel solution and covered with olive oil, to be further incubated at 37°C during 4 h. Posteriorly the hydrogel was removed from the tissue to be washed twice with SDS clearing solution (1M Boric Acid pH 8.5, SDS 4%) 24 h, at room temperature. Samples where then incubated at 50ºC in SDS clearing solution until transparency was achieved. After clearing, samples were thoroughly washed in a Triton-X 10%, NaN3 2% solution during 24 h, twice, at 37ºC. Samples were then incubated in Synaptophysin primary antibody (Supp. Table. 16) at 1:50 at 37°C for 5 days, to be washed again 24 h, twice, at 37ºC and incubated in secondary antibody at 1:50 at 37°C for 5 days. A final washing step of 24 h, twice, at 37ºC was performed. Samples were incubated in 87% glycerol 3 h at room temperature prior to imaging and fixed flat to the bottom of an imaging dish with 63% TDE. A Z-stack of a minimum thickness of 100 µm was acquired with a Leica TCS SP5 confocal microscope (Leica microsystems, Wetzlar, Germany). 3D synaptophysin positive particle counting was performed using the 3D objects counting plugin on ImageJ 1.52p (NIH, USA).

Biochemical methods

Preparation of Soluble and Insoluble protein fractions. Soluble and insoluble fractions from brain tissue were isolated as described by Tremblay C and colleagues (3). Briefly, temporal cortex from SAD and FAD patients (~100 mg) was homogenized in 4 volumes of Tris Buffered Saline (TBS) containing a cocktail of phosphatase and protease inhibitors (Roche, Mannheim, Germany). Samples were sonicated three times for 10 s and centrifuged at 100,000 g for 20 min at 4°C to obtain a TBS-soluble fraction containing cytosolic and extracellular proteins (Soluble fraction). The pellet was sonicated using 4 volumes of lysis buffer (150 mM NaCl, 10 mM NaH2PO4, 1% Triton X-100, 0.5% SDS, and 0.5% deoxycholate) with protease and phosphatase inhibitors. The homogenate was centrifuged at 100,000 g for 20 min at 4°C. The pellet was homogenized in 200 µl of 90% formic acid and sonicated three times for 10 s to isolate the Insoluble protein fraction. Protein fractions were stored at -80ºC for further experiments.

Extraction of total protein from tissue. Temporal cortex from Control, SAD, E-AOFAD and LOFAD cases (Table 3) were cleared of meninges and only grey matter was used for the procedure. Approximately 250 mg of tissue were cut in small pieces, poured into a glass Dounce tissue grinder type B and homogenized with ten even strokes in 1 mL of lysis buffer containing 150 mM NaCl, 20 mM Tris pH 7.4, 1 mM EDTA, 10% Glycerol, 1% NP40 and a cocktail of phosphatase and protease inhibitors (Roche, Mannheim, Germany). The homogenate was centrifuged at 13,000 g for 10 min at 4ºC and the proteins present in the supernatant were

quantified using the bicinchoninic acid method (BCA Protein Assay Kit, Thermo, Dreieich, Germany). The protein extracts were stored at -80ºC for further experiments.

Western blotting. Sodium dodecyl sulfate polyacrylamide gel electrophoresis (SDS-PAGE). Once proteins were quantified, SDS-PAGE was carried out using a 20 well electrophoresis system (VWR, Radnor, PA, USA) or a Miniprotean system (BioRad, München, Germany). Samples were mixed with loading buffer (0.375 M Tris pH 6.8, 50% glycerol, 10% SDS, 0.5 M DTT and 0.002% bromophenol blue) and heated to 95ºC for 5 minutes. About 25 – 30 μg of protein were loaded into each well. After electrophoresis, proteins were transferred to nitrocellulose membranes (BioRad, München, Germany) using a Trans-blot Turbo Transfer system (BioRad, München, Germany) at 300 mA for 2 h. The membranes were incubated for 1 h in 5% non-fat milk dissolved in TTBS (100 mMTris pH 7.5, 500 mMNaCl, 0.02% Tween-20) and incubated overnight at 4°C with primary antibody (Table 16). Subsequently, membranes were washed with TTBS and incubated with secondary antibody (Table 17) coupled to peroxidase for 1 h at room temperature. Immunoreactive signal was developed with the ECL Western Blotting chemiluminescence system (SuperSignal West Pico Chemiluminiscent Substrate, Thermo, Dreieich, Germany) and detected with a ChemiDoc system (BioRad, München, Germany). For some of total vs phosphorylated protein kinase blotting where the species of each antibody allowed it, the same membrane was first incubated with the phosphorylated antibody, it was developed and re-incubated with the total protein antibody of a different species. The images were analyzed using the quantification software QuantityOne (BioRad, München, Germany). The results of each sample were normalized to GAPDH and compared between groups. To minimize interassay variation, the samples from all experimental groups were processed in parallel. Regarding Aβ oligomers, there is an intrinsic difficulty in distinguishing between Aβ oligomers and APP fragments still containing the Aβ sequence (4) and although it has been suggested that Aβ oligomers rather than plaques are the main factor in Aβ-related pathogenicity, existing evidence regarding their possible role in AD is unclear (5). Therefore, only small oligomers (< 40 kDa) were considered for quantification.

Tricine gel electrophoresis. TBS soluble fractions from temporal cortex were loaded into each well with loading buffer (0.15 M Tris pH 6.8, 36% Glycerol, 12% SDS, 0.3 M DTT, 0.002% Coomasie Blue) and heated to 95ºC for 5 minutes before loading on precast 10%-20% Tricine gels (Thermo, Dreieich, Germany). The gels were run using cathode buffer (1 M Tris base, 1 M Tricine and 1% SDS) anode buffer (1 M Tris base and 0.225 M HCl) and transferred to nitrocellulose membranes (BioRad, München, Germany) using an electrophoretic transfer system (BioRad, München, Germany) at 300 mA for 80 min. Membranes were blocked, incubated with primary and secondary antibodies (Table 17 and 18) and detected as described above.

Aβ peptides analysis. Brain tissue (frontal cortex) from SAD and PSEN1 E280A subjects (Table 3) was homogenized on ice in Tris-buffered saline containing complete protease inhibitor (Roche Diagnostics GmBH, Mannheim, Germany) as described previously (6). Briefly, formic acid (FA) was added (final concentration 70%) followed by further homogenization, sonication and centrifugation (30,000g, 1 h, +4°C). The supernatant was collected and dried in a vacuum centrifuge. The dried fraction was dissolved in 70% FA followed by centrifugation (30,000g, 1 h, +4°C). Before IP, the supernatant was neutralized using 0.5 M Tris.Aβ peptides were immunoprecipitated using Aβ-specific antibodies coupled to magnetic beads as described previously (6). Briefly, 4 μg of the anti-Aβ antibodies 6E10 and 4G8 (Signet Laboratories, Dedham, MA, USA) was separately added to 50 μL each of magnetic Dynabeads M-280 Sheep Anti-Mouse IgG (Invitrogen, Carlsbad, CA, USA). The 6E10 and 4G8 antibody-coated beads were mixed and added to the CSF samples to which 0.025% Tween20 in phosphate-buffered saline (pH 7.4) had been added. After washing, the Aβ isoforms were eluted using 100 μL 0.5% FA. Mass spectrometry was performed using a matrix-assisted-laser-desorption/ionizationtime-of-flight/time-of-flight (MALDI TOF/TOF) instrument (UltraFleXtreme, Bruker Daltonics, Bremen, Germany). Samples were prepared as described previously (6).

In vitro gamma secretase activity assay. Detergent resistant membrane preparation from human SAD (n=5) and FAD brains (n=23) CHAPSO resistant membranes were prepared for human brains (frontal cortex) which were frozen within 12 h postmortem as previously described in (7) with minor modifications. After carefully removing leptomeninges and blood vessels, < 250 mg blocks of tissue were homogenized in ~ 10 volumes of 10% sucrose in MES buffer (25 mM MES, pH 6.5, 150 mMNaCl) containing 1% CHAPSO (Sigma) and protease inhibitors. The homogenate was mixed with equal volume of 70% sucrose in MES buffer. 4 ml was placed at the bottom of an ultracentrifuge tube (Beckman, 344059) and overlaid with 4 ml of 35% sucrose and 4 ml of 5% sucrose prepared accordingly. The obtained gradients were centrifuged at 39,000 rpm for 20 h at 4°C on a SW 41Ti rotor (Beckman). After centrifugation the raft fraction (interface of 5%/35% sucrose) was carefully collected and re-centrifuged (50,000 rpm, 60 min, 4°C) in 20 mM PIPES, pH 7, 250 mM sucrose and 1M EGTA. The resultant pellet was re-suspended with above buffer using a 26G syringe and stored at -80°C until use. We adjusted CHAPSO resistant membrane fractions to 1 μg/μl in protein concentration with 20 mM PIPES, pH 7.0, 250 mM sucrose and 1 mM EGTA. To determine the novo production of Aβ peptides, 6 μg CHAPSO resistant membranes were incubated for 0 h or 4 h at 37°C with 1.5 μM C99-3XFLAG substrate. The activity assays were carried out in presence of 2.5% DMSO (or 1μM GSM in DMSO), 1mM EGTA, 0.3% Chapso and protease inhibitors. Reactions were loaded on the 4 spot MSD ELISA plate and Aβ38, Aβ40 and Aβ42 quantified. Ninety-six-well MULTI-SPOT SECTOR plates from Meso Scale Discovery (Mesoscale Cat# N45ZA-1) were pre-coated by the company with Janssen capturing antibodies (Aβ37, Aβ38, Aβ40, Aβ42) 300 μg/ml and stored at 4°C until use. Plates were brought to room temperature 30 min prior use and blocked with 150 μl/well 0.1 % casein buffer for 1.5 h at room temperature (600 rpm). After incubation, plates were rinsed 5 x with 200 μl/well washing buffer (PBS + 0.05 % Tween-20). Samples and standards (synthetic human Aβ1-38, Aβ1-40, Aβ1-42 peptides) were diluted in 0.1% casein buffer and loaded 25 μl of the sample mixed with 25 μl detection antibody (JRF/AβN/25) to the plate. After overnight incubation at 4°C, plates were rinsed and 150 μl/well of the 2x MSD read buffer T was added. The plates were immediately developed on MSD Sector Imager 6000. We determined the rates at which Aβ38, Aβ40 and Aβ42 are produced in each sample by subtracting the 0 h value from the 4 h value obtained by MSD ELISA and normalizing Aβ amounts against time to express rates in pM/h. The experiment was replicated three times for all FAD cases and three out of five SAD cases.

Kinome profile characterization. 50 mg of temporal cortex from selected cases (Table 3) was lysed at 0 °C using M-PER (Mammalian Protein Extraction Reagent, Thermo Scientific, MA, USA) lysis buffer (0.1 g/ml) containing Protease Inhibitor Cocktail (Roche, Manheim, Germany) and Phosphatase Inhibitor Cocktail (Roche, Manheim, Germany), and centrifuged at 10.000× g 10 min, 4 °C. Supernatants we snap frozen in 100μl aliquots and stored at –80 °C. The protein concentration was determined using the Bradford Lowry Assay (Pierce Coomasie assay, Thermo, Dreieich, Germany). Frozen aliquots were never re-frozen but used directly for kinase activity determination. Kinase activity profiles were determined using the PamChip ® 96 serine/threonine (STK) and protein tyrosine (PTK) peptide microarray system from PamGene International B.V. (‘s-Hertogenbosch, The Netherlands) according to the instructions of the manufacturer, and as described previously (8). All PamChip ® 96 array plates used in this study came from the same production batch and all plates were run on the same PamStation instrument. For each assay, 0.5μg of protein was used. Arrays were incubated 30 cycles in blocking buffer and 60 cycles in reaction buffer containing ATP (final concentration 100 μM; Sigma-Aldrich, St. Louis, MO, USA). Arrays were washed and incubated for 60 min with a secondary antibody (polyclonal swine anti-rabbit Immunoglobulin/FITC). Images at 50 ms exposure time were captured every 10 min with an integrated CCD-based optical system in combination with Evolve software (version 1.5, PamGene International BV). After removal of the secondary antibody and a wash step, post-wash images were taken at different exposure times (20, 50, 100, and 200 ms). The PTK assay mixture contained the same kinase assay buffer, 100μM ATP and 0.01% BSA, supplemented with 4μl protein kinase (PK)-additive (PamGene International BV), 10 mMDithiothreitol (DTT, Fluka, Sigma-Aldrich, St. Louis, MO, USA) and fluorescein isothiocyanate (FITC) labeled anti-phosphotyrosine antibody (PamGene International BV, ‘s-Hertogenbosch,

The Netherlands). For each PTK assay, 7.5µg of protein was used. Since a labeled antibody is present in the PTK assay mixture, peptide phosphorylation was monitored during the incubation with assay mixture, by taking images every 5min at 50 ms exposure time, allowing real time recording of the reaction kinetics (one-step reaction). After washing of the array, fluorescence was detected at different exposure times (20, 50, 100, and 200 ms). The fluorescent signal intensity for each peptide was analyzed using BioNavigator 6.1 software (PamGene International BV,'s-Hertogenbosch, The Netherlands) a statistical analysis and visualization software tool with an App-based infrastructure (https://www.pamgene.com/en/bionavigator.htm). For signal quantification, the slope of the fluorescent signal versus exposure time was calculated in order to increase the dynamic range and to filter out time differences between plates. Saturated signals were excluded. Visual quality control was performed to exclude defective arrays from the analysis. A linear mixed-effects model that analyzed the signals of all peptides jointly while taking the correlation structure into account was used. Change of log (signals) over log (time) was calculated. The obtained STK and PTK median kinase signal intensities were analyzed for common effects (for all peptides) and peptide-specific plate, strip and array random effects. The measurement error was modeled using a peptide-specific variance component covariance matrix that allowed for heterogeneous variances among exposure time points. The upstream protein kinases able to phosphorylate residues in peptides on the PTK and STK arrays were identified in the Human Protein Reference Database (http://www.hprd.org) (9), in Phosphosite (http://www.phosphosite.org) and Reactome (http://www.reactome.org) (10). These kinases were projected on the kinase phylogenetic tree using the Kinome Render tool (http://bcb.med.usherbrooke.ca/kinomerender.php). When databases used different names to indicate a kinase, the kinase names were converted to those used in Kinome Render via their UniProtID. For kinases linked to multiple UniProtIDs, only the ID used in the Kinome Render tool was retained (8).

Chymotrypsin 20S proteasome activity assay. Chymotrypsin 20S proteasome activity was tested in temporal cortex from controls and PSEN1 E280A cases using the 20S proteasome activity assay kit APT280 (Millipore-Merck, Darmstadt, Germany), following manufacturer instructions. Briefly, 20 mg of tissue was dounce homogenized with 20 strokes in 1:8 homogenization buffer (50 mM HEPES, 250 mM sucrose, 5 mM MgCl2, 0.5 mM DTT, 40 mMKCl pH 7.4), and centrifuged at 10,000g x 15 min. Suc-LLVY-AMC substrate 20S 12,5-0,1 uM dilution and 20S proteasome positive control 1:4 – 1:512 dilution were served on a 96-well plated together with 20 ug of of protein from samples by triplicate with and without Lactacystin 5 ul 500 uM. Plate was incubated 2 h at 37ºC and read using a 380/460 nm filter. Standard curves were calculated from AMC substrate and proteasome positive control dilutions, tissue samples activity was calculated substracting values with inhibitor from inhibitor-free samples.

pTau Seeding assay. Finally, 300 mg of frozen temporal cortices were homogenized in 1500 µL of PBS + protease inhibitor (Roche) in a 2 mL glass dounce homogenizer (30 up/down strokes on ice by hand). The homogenate was transferred to a 1.5 mL Eppendorf tube and centrifuged at 10,000 x g for 10 min at 4°C. The supernatant was collected and aliquoted to avoid excessive freeze/thaw cycles. A bicinchoninic acid assay (BCA, Thermo Scientific Pierce) was performed to determine total protein concentration following the manufacturer's protocol. The in vitro seeding assay has been previously described and widely characterized (18, 19). Briefly, The Tau RD P301S FRET Biosensor (ATCC® CRL-3275™) cells stably expressing the repeat domain of Tau with the P301S mutation conjugated to either the cyan fluorescent protein (CFP) or the yellow fluorescent protein (YFP) (TauRD-P301S-CFP/YFP) were cultured at 37°C, 5% CO2 in DMEM, 10% v/v fetal bovine serum, 0.5% v/v penicillin/streptomycin. Cells were plated on Costar Black, clear bottom 96-well plates (previously coated with 1:20 poly-D-lysine) at a density of 40,000 cells per well. Brain extracts (1 µg of total protein quantified by BCA per well) were then incubated with Lipofectamine 2000 (Invitrogen, final concentration 1% v/v) in opti-MEM (final volume 50 µL per well) for 10 min at room temperature before being added to the cells. Each condition was applied in triplicate or quadruplicate. After 24 h, Tau seeding was subsequently analysed using flow cytometry : Medium was removed and 50 µL trypsin 1x was added for 7 min at 37°C. Chilled DMEM + 10% fetal bovine serum (150 µL) was added to the trypsin and cells were transferred

to 96-well U-bottom plates (Corning). Cells were pelleted at 500 x g, resuspended in freshly-made 2% v/v paraformaldehyde in PBS (Electron Microscopy Services) for 10 min at room temperature in the dark, and pelleted at 500 × g. Cells were resuspended in chilled PBS and run on the MACSQuant VYB (Miltenyi) flow cytometer. CFP and Forster resonance energy transfer (FRET) were both measured by exciting the cells using the 405 nm laser and reading fluorescence emission at the 405/50 nm and 525/50 nm filters, respectively. To quantify the FRET signal, a bivariate plot of FRET vs. the CFP donor was generated and cells that received control brain extract alone were used to identify the FRET-negative population. Using this gate, the integrated FRET density (IFD) value for each well was calculated by multiplying the percent of FRET-positive cells by the median fluorescence intensity of that FRET-positive population. 40,000 events per well were analysed. Data was analysed using the MACSQuantify software (Miltenyi).

Genetic and protein network analysis methods

Fourteen patients with PSEN1 E280A FAD placed at the extremes of the AoO distribution (Table 3) were included for whole-exome capture (WEC). DNA was extracted from brain tissue and genomic DNA was processed by the Australian Genome Facility (Melbourne, VIC, Australia), an Illumina Certified Service Provider for the Infinium Genotyping Service, using the Infinium assay. DNA libraries were constructed from 1 µg of genomic DNA using an Illumina TruSeq genomic DNA library kit (Illumina Inc., San Diego, CA, USA). Libraries were multiplexed with 6 samples pooled together (500 ng of each). Exons were enriched from the pooled 3 µg of library DNA using an Illumina TruSeq Exome enrichment kit (Illumina Inc.). Each exome-enriched pool was run on a 100-base-pair paired-end run on an IlluminaHiSeq 2000 sequencer (Illumina Inc.). We surveyed 201,071 genomic regions in total using the exome capture platform. All regions were sampled at ~50Xcoverage. Sequencing image data were processed in real time using Illumina Real Time Analysis (RTA) software (Illumina Inc., San Diego, CA, USA), and converted to suitable formats using the CASAVA pipeline from Illumina. The resulting FASTQ files were further processed for variant analysis using Golden Helix®'s SNP variation suite (SVS) 8.3.0 (Golden Helix, Inc. Bozeman, MT, USA). The entire workflow of data curation and analysis for variant calling was developed by the Genome Discovery Unit at The Australian National University and consists of the following key steps: (i) quality assessment; (ii) read alignment; (iii) local realignment around the known and novel indel regions to refine indel boundaries; (iv) recalibration of base qualities; (v) variant calling; and (vi) assigning quality scores to variants as described elsewhere (11,12). Genotype files were processed in SVS 8.3.0. Samples with calls below Illumina®'s expected 99% SNVs call rates were excluded. Single nucleotide variants (SNVs) were excluded when (i) deviated from Hardy-Weinberg equilibrium with P< 2x10-7, (ii) the minimum genotype call rate was <90%, (iii) the number of alleles was one or more than two, and (iv) the MAF<1%. Genotype and allelic frequencies were estimated by maximum likelihood. Subsequently, a filtering phase including the identification of de novo SNVs; filtering of potentially pathogenic variants using SIFT PolyPhen-2 MutationTaster, Gerp++ and PhyloP; and filtering of damaging variants based on genes known to be associated with AD, was performed using information from dbSNP and the 1K Exome Project. De novo SNVs were defined according to the DNA-seq Analysis module in SVS 8.3.0. Potential relationships between AoO and SNVs were individually examined using one-way analysis of variance (ANOVA). P-values were obtained based on the F-statistic and corrected for multiple testing using the false discovery rate (FDR) and a method based on extremes-value theory, as explained elsewhere (13). Network analysis and pathway analysis was performed using NetworkAnalyst (14) webpage tools and Cytoscape software (15). Protein – protein interaction was assessed InnateDB (16) webpage tools.

General statistical methods

Data was analyzed using IBM SPSS Statistics 22 software (IBM/SPSS Inc., Armonk NY, USA), GraphPad Prism 6 (GraphPad Software, Inc., La Jolla, CA, USA) and R statistical software (R Foundation for Statistical Computing, Vienna, Austria). Analyses included distribution analysis, Hartigan's dip test, nonparametric tests

and $\chi 2$ square test for categorical variables comparisons. ANOVA and logistic regression analysis were applied to AoO, cognitive variables and schooling time. One-way ANOVA and Kruskal-Wallis test were used for group comparison for demographic, neuropathological and biochemical variables. The U-Mann-Whitney (given as Z) nonparametric test was used for two group comparisons, when indicated. Correlation analysis was performed using Spearman's ρ test. Statistical significance of all analyses was determined with * $p \leq 0.05$, ** $p \leq 0,01$ and *** $p \leq 0,001$.

References

1. Acosta-Baena N, Sepulveda-Falla D, Lopera-Gómez C et al. Pre-dementia clinical stages in presenilin 1 E280A familial early-onset Alzheimer's disease: a retrospective cohort study. The Lancet Neurology 2011; 10: 213-220.
2. Sepulveda-Falla D, Matschke J, Bernreuther C et al. Deposition of hyperphosphorylated Tau in cerebellum of PSEN1 E280A Alzheimer's Disease. Brain Pathology 2011; 21: 452-463
3. Tremblay C, Pilote M, Phivilay A, Emond V, Bennett D, Calon F. Biochemical characterization of Aβ and Tau pathologies in mild cognitive impairment and Alzheimer's Disease. Journal of Alzheimer's Disease 2007; 12: 377-390.
4. Willem M, Tahirovic S, Busche MA et al. η-Secretase processing of APP inhibits neuronal activity in the hippocampus. Nature 2015; 526: 443-447.
5. Benilova I, Karran E, De Strooper B. The toxic Aβ oligomer and Alzheimer's disease: an emperor in need of clothes. Nature Neuroscience 2012; 15: 349-357.
6. Portelius E, Tran A, Andreasson U et al. Characterization of Amyloid β peptides in cerebrospinal fluid by an automated immunoprecipitation procedure followed by Mass Spectrometry. Journal of Proteome Research 2007; 6: 4433-4439.
7. Kakuda N, Shoji M, Arai H et al. Altered γ-secretase activity in mild cognitive impairment and Alzheimer's disease. EMBO Molecular Medicine 2012; 4: 344-352.
8. Rosenberger AF, Hilhorst R, Coart E et al. Protein kinase activity decreases with higher Braak stages of Alzheimer's Disease Pathology. Journal of Alzheimer's Disease 2015; 49: 927-943.
9. Keshava Prasad TS, Goel R, Kandasamy K et al. Human Protein Reference Database--2009 update. Nucleic Acids Research 2009; 37: D767-D772.
10. Matthews L, Gopinath G, Gillespie M et al. Reactome knowledgebase of human biological pathways and processes. Nucleic Acids Research 2009; 37: D619-D622.
11. Vélez JI, Rivera D, Mastronardi C et al. A Mutation in DAOA modifies the age of onset in PSEN1 E280A Alzheimer's Disease. Neural Plasticity 2016; 2016: 1-7.
12. Johar A, Mastronardi C, Rojas-Villarraga A et al. Novel and rare functional genomic variants in multiple autoimmune syndrome and Sjögren's syndrome. Journal of Translational Medicine 2015; 13:173.
13. Vélez JI, Lopera F, Sepulveda-Falla D et al. APOE*E2 allele delays age of onset in PSEN1 E280A Alzheimer's disease. Molecular Psychiatry 2015; 21: 916-924.
14. Xia J, Benner MJ, Hancock RE. NetworkAnalyst--integrative approaches forprotein-protein interaction network analysis and visual exploration. NucleicAcids Res. 2014 Jul; 42:W167-74.
15. Shannon P, Markiel A, Ozier O et al. Cytoscape: a software environment for integrated models of biomolecular interaction networks. Genome Res. 2003 Nov;13(11):2498-504.
16. Breuer K, Foroushani AK, Laird MR, Chen C, Sribnaia A, Lo R, Winsor GL, Hancock RE, Brinkman FS, Lynn DJ. InnateDB: systems biology of innate immunity and beyond--recent updates and continuing curation. Nucleic Acids Res. 2013 Jan; 41:D1228-33.
17. Liu AK, Hurry ME, Ng OT et al. Bringing CLARITY to the human brain: visualization of Lewy pathology in three dimensions. Neuropathol Appl Neurobiol. 2016 Oct;42(6):573-87.
18. Holmes BB, Furman JL, Mahan TE, et al. Proteopathic tau seeding predicts tauopathy in vivo. Proc Natl Acad Sci U S A. 2014;111(41):E4376-E4385.

19. Dujardin S, Commins C, Lathuiliere A, Beerepoot P, Fernandes AR, De Los Santos MB, Klickstein N, Corjuc DL, Corjuc BT, Dooley PM, Viode A, Oakley DH, Moore BD, Mullin K, Jean-Gilles D, Clark R, Atchison K, Moore R, Chibnik LB, Tanzi RE, Frosch MB, Serrano-Pozo A, Elwood F, Steen JA, Kennedy ME, Hyman BT. Tau molecular diversity contributes to clinical heterogeneity in Alzheimer's disease., Nat Med. 2020. 6(8):1256-1263.

Tables

Table 1. Demographic and cognitive performanceaccording to age of onset in 122 PSEN1 E280A patients

Group	Patients	Gender (F)	ApoE2	ApoE3	ApoE4
**Q1: Early**	33	72.7%	--	73.3 %	26.6 %
**Q2-Q3: Average**	61	57.4%	3.6%	70.9 %	25.5 %
**Q4: Late**	28	53.6%	8.0 %	64.0 %	28.0 %
		X Squared 0.237	X Squared 0.622, *(-12)		
		X Squared	*0.012*	0.710	0.934
**Total**	122	60.7 %	3.6 %	70.0 %	26.4 %

		Age of Onset			p value vs			Schooling			p value vs		
**Group**	**Patients**	**Mean**	**SD**	**Range**	**Q1**	**Q2/Q3**	**Q4**	**Mean**	**SD**	**Range**	**Q1**	**Q2/Q3**	**Q4**
**Q1: Early**	33	44.21	1.95	39 - 46	--	*0.000*	*0.000*	6.76	3.99	1 - 16	--	0.400	*0.018*
**Q2-Q3: Average**	61	49.18	1.64	47 - 52	*0.000*	--	*0.000*	5.49	3.88	0 - 18	0.400	--	0.261
**Q4: Late**	28	56.96	3.79	53 - 70	*0.000*	*0.000*	--	3.96	3.74	0 - 13	*0.018*	0.261	--
						p ANOVA						p ANOVA	
**Total**	122	49.62	5.11	39 - 70		*0.000*		5.48	3.97	0 -18		*0.022*	

		MMSE			p value vs			Memory			p value vs		
**Group**	**Patients**	**Mean**	**SD**	**Range**	**Q1**	**Q2/Q3**	**Q4**	**Mean**	**SD**	**Range**	**Q1**	**Q2/Q3**	**Q4**
**Q1: Early**	33	19.06	5.48	9 - 30	--	0.384	0.260	-0.47	0.45	-1.20 - 0.80	--	0.094	0.097
**Q2-Q3: Average**	61	17.23	5.57	6 - 27	0.384	--	1.000	-0.64	0.30	-1.10 - 0.07	0.094	--	1.000
**Q4: Late**	28	16.61	5.50	4 - 25	0.260	1.000	--	-0.67	0.36	-1.25 - 0.19	0.097	1.000	--
						p ANOVA						p ANOVA	
**Total**	122	17.58	5.56	4 - 30		0.180		-0.60	0.37	-1.25 - 0.8		0.051	

		Language			p value vs			Praxis			p value vs		
**Group**	**Patients**	**Mean**	**SD**	**Range**	**Q1**	**Q2/Q3**	**Q4**	**Mean**	**SD**	**Range**	**Q1**	**Q2/Q3**	**Q4**
**Q1: Early**	33	-0.36	0.48	-1.46 - 0.30	--	0.737	*0.037*	-0.60	0.87	-1.81 - 0.81	--	*0.036*	0.080
**Q2-Q3: Average**	61	-0.50	0.60	-1.87 - 0.63	0.737	--	0.245	-0.99	0.64	-1.81 - 0.71	*0.036*	--	1.000
**Q4: Late**	28	-0.74	0.64	-2.00 - 0.63	*0.037*	0.245	--	-1.01	0.62	-1.81 - 26	0.080	1.000	--
						p ANOVA						p ANOVA	
**Total**	122	-0.52	0.59	- 2.0 - 0.63		*0.042*		-0.89	0.73	-1.81 - 0.81		*0.026*	

Q = quartile, F = Female, SD = Standard Deviation. *= missing cases. Significant values are written in cursive.

Table 2. Logistic Regression of Age of Onset and Schooling time as predictors of cognitive domains scores in PSEN1 E280A Age of Onset subgroups

					Coefficient B		Significance	
**Variable**	**F**	**df**	**p**	**R^2**	**Age of Onset**	**Schooling**	**Age of Onset**	**Schooling**
MMSE	7.518	2 , 119	*0.001*	0.112	-0.070	0.438	0.476	*0.001*
Memory domain	5.626	2 , 119	*0.005*	0.086	-0.007	0.023	0.274	*0.006*
Language domain	11.864	2 , 119	*0.000*	0.166	-0.018	0.050	*0.008*	*0.004*
Praxis domain	10.670	2 , 119	*0.000*	0.152	-0.018	0.062	0.155	*0.000*

Table 3. Cases used for genetic, biochemical and pathological studies

Case	Genotype	Group/Onset	Sex	Age of Onset	Age of Death	Disease Duration	Postmortem Index	ApoE Haplotype	Histopathology	Aβ Oligomers / pTau fractions	Mass Spectometry	Aβ de novo generation	Synaptic Density Analysis	Tau Kinsaes analysis	Kinome Analysis	WES genetic analysis	Proteasome S20 activity	Polyubiquitinated WB and IP	pTau Seeding Assay
1	none	Ctrl	F	--	73	--	945	--	-	-	-	+	-	+	-	-	-	-	-
2	none	Ctrl	M	--	86	--	635	--	-	-	-	+	-	+	-	-	+	-	-
3	none	Ctrl	M	--	67	--	880	--	-	-	-	+	-	+	+	-	+	-	-
4	none	Ctrl	M	--	61	--	270	--	-	-	-	+	-	+	+	-	+	-	-
5	none	Ctrl	M	--	70	--	120	--	-	-	-	+	-	+	+	-	+	-	-
6	none	SAD	M	NA	67	--	558	3/3	+	+	-	-	-	+	-	-	-	-	-
7	none	SAD	M	80	86	6	1098	NA	+	+	-	-	-	+	-	-	-	-	-
8	none	SAD	F	55	70	15	708	3/4	+	+	-	-	-	+	-	-	-	-	-
9	none	SAD	F	79	87	8	168	3/4	+	-	-	-	-	+	+	-	-	-	-
10	none	SAD	F	82	91	9	270	3/3	+	+	+	-	-	+	+	-	-	-	-
11	none	SAD	F	65	74	9	150	3/3	+	+	+	-	-	+	-	-	-	-	-
12	none	SAD	F	65	76	11	240	4/4	+	+	+	-	-	+	+	-	-	-	-
13	none	SAD	F	69	76	7	400	3/4	+	+	+	-	-	+	+	-	-	-	-
14	none	SAD	M	NA	83	--	270	3/2	+	+	+	-	-	+	-	-	-	-	-
15	none	SAD	F	NA	61	--	462	3/3	+	+	+	-	-	+	+	-	-	-	-
16	E280A	Early-FAD	F	37	47	10	138	NA	+	+	+	+	-	+	-	-	+	+	-
17	E280A	Early-FAD	F	39	59	20	222	3/3	+	+	+	+	+	+	+	+	+	+	-
18	E280A	Early-FAD	F	40	59	19	360	3/3	+	+	+	+	+	+	+	+	-	-	+
19	E280A	Early-FAD	F	40	42	2	330	3/3	+	+	+	+	-	+	-	-	+	+	+
20	E280A	Early-FAD	F	42	50	8	450	3/4	+	+	+	+	+	+	-	+	-	-	+
21	E280A	Early-FAD	F	43	57	14	240	3/4	+	+	+	+	-	+	-	-	-	-	-
22	E280A	Early-FAD	M	44	52	8	288	3/3	+	+	+	+	+	+	+	-	-	-	+
23	E280A	Early-FAD	F	46	66	20	240	3/3	+	+	+	+	+	+	+	-	-	-	+
24	E280A	Average-FAD	F	47	54	7	330	3/3	+	+	+	+	+	+	+	-	-	-	-
25	E280A	Average-FAD	M	47	56	9	198	3/3	+	+	+	+	+	+	-	-	-	-	+
26	E280A	Average-FAD	M	47	58	11	210	3/3	+	+	+	+	+	+	+	-	-	-	-
27	E280A	Average-FAD	F	48	64	16	180	3/3	+	+	+	+	-	+	+	-	+	+	-
28	E280A	Average-FAD	F	49	62	13	240	4/4	+	+	+	+	+	+	+	+	+	+	+
29	E280A	Average-FAD	M	49	55	6	168	3/3	+	+	+	+	-	+	+	+	+	-	-
30	E280A	Average-FAD	F	50	60	10	168	3/3	+	+	+	+	+	+	-	+	-	-	+
31	E280A	Late-FAD	F	52	68	16	384	3/3	+	+	+	+	+	+	+	+	+	+	+
32	E280A	Late-FAD	M	53	60	7	240	3/4	+	+	+	+	+	+	+	+	+	-	+
33	E280A	Late-FAD	M	54	63	9	330	3/3	+	+	+	+	-	+	-	+	+	+	+
34	E280A	Late-FAD	F	55	64	9	180	NA	+	+	+	+	-	+	+	+	+	+	-
35	E280A	Late-FAD	M	56	63	7	228	NA	+	+	+	+	+	+	+	+	+	+	+
36	E280A	Late-FAD	F	58	71	13	300	3/3	+	+	+	+	+	+	+	+	+	+	+
37	E280A	Late-FAD	F	58	70	12	192	3/3	+	+	+	+	+	+	-	+	+	+	+
38	E280A	Late-FAD	F	62	74	12	330	3/3	+	+	+	+	-	+	+	+	+	+	+

Table 4. Demographic and clinical characterization of PSEN1 E280A patients used for biochemical and pathological studies

Group	Subgroup	N	Gender (F)	ApoE				Memory Impairment	Language Impairment	Parkinsonism	Seizures / Myoclonus	Abnormal gait	Behavioral changes	Depression	Cerebellar Signs	Headache	Sleep Disorder
				ApoE2	ApoE3	ApoE4	NA										
Control		5	20.0%	--	--	--	100.0%										
SAD		10	70.0%	10.0%	40.0%	40.0%	10.0%										
	Early	8	87.5%	0.0%	62.5%	25.0%	12.5%	100%	100%	87.5%	75%	75%	50%	50%	50%	37.5%	25%
FAD	Average	7	57.1%	0.0%	85.7%	14.3%	0.0%	100%	100%	100%	100%	71.4%	71.4%	71.4%	57.1%	28.5%	28.6%
	Late	8	62.5%	0.0%	62.5%	12.5%	25.0%	100%	100%	100%	87.5%	87.5%	62.5%	62.5%	37.5%	37.5%	50%
	X^2 FAD subgroups		0.747	----	0.828	0.813	----	1.000	1.000	0.958	0.874	0.932	0.866	0.866	0.855	0.946	0.660
	Total FAD	23	56.5%	0.0%	69.6%	17.4%	13.0%	100%	100%	95.7%	87%	78.3%	60.9%	60.9%	47.8%	34.8%	34.8%
	X^2Total		0.174	0.579													

EOFAD = Early onset FAD, AOFAD = Average onset FAD, LOFAD = Late onset FAD

Table 5. Demographic and clinical characterization of PSEN1 E280A patients used for biochemical and pathological studies (cont.)

Group		N	Age of Onset			p value vs			Age of Death			p value vs		
			Mean	SD	Range	EOFAD	AOFAD	LOFAD	Mean	SD	Range	EOFAD	AOFAD	LOFAD
**Control**		5	--	--	--	--	--	--	71.40	9.29	61.0 - 86.0	--	--	--
**SAD**		10	70.71	9.98	55 - 82	*0.000*	*0.000*	*0.000*	77.10	9.60	61.0 - 91.0	*0.000*	*0.000*	*0.014*
	**EOFAD**	8	41.38	2.93	37 - 46	--	*0.021*	*0.000*	54.00	7.71	42.0 - 66.0	--	0.243	*0.006*
	**AOFAD**	7	48.14	1.22	47 - 50	*0.021*	--	*0.017*	58.43	3.74	54.0 - 64.0	0.243	--	*0.070*
	**LOFAD**	8	56.00	3.25	52 - 62	*0.000*	*0.017*	--	66.63	4.84	60.0 - 74.00	*0.006*	*0.070*	--
						**SAD**						**SAD**		
**FAD**		23	48.52	6.74	37 - 62	*0.000*			59.39	8.15	42.0 - 74.0	*0.000*		

Group		N	Disease Duration			p value vs			Post Mortem Index			p value vs		
			Mean	SD	Range	EOFAD	AOFAD	LOFAD	Mean	SD	Range	EOFAD	AOFAD	LOFAD
**Control**		5	--	--	--	--	--	--	570.00	365.19	120 - 945	1.000	0.291	1.000
**SAD**		10	9.29	2.98	6.0 - 15.0	0.640	0.917	0.917	432.40	293.66	150 - 1098	1.000	0.313	1.000
	**EOFAD**	8	12.63	6.67	2.0 - 20.0	--	0.851	0.851	283.50	95.99	138 - 450	--	1.000	1.000
	**AOFAD**	7	10.29	3.45	6.0 - 16.0	0.851	--	0.917	213.43	57.43	168 - 330	1.000	--	1.000
	**LOFAD**	8	10.63	3.16	7.0 - 16.0	0.851	0.917	--	273.00	73.55	180 - 384	1.000	1.000	--
						**SAD**						**SAD**		
**FAD**		23	10.83	4.85	2.0 - 21.0	0.471			258.52	80.64	138 - 450	0.105		

EOFAD = Early onset FAD, AOFAD = Average onset FAD, LOFAD = Late onset FAD

Table 6. Aβ pathology according Age of Onset in PSEN1 E280A FAD

Group	N	Aβ FC			p value vs				Aβ TC			p value vs			
		Mean	SD	Range	SAD	EOFAD	AOFAD	LOFAD	Mean	SD	Range	SAD	EOFAD	AOFAD	LOFAD
**SAD**	10	49640.43	23159.40	23601.48 - 94648.09	--	1.000	0.592	1.000	32218.77	15596.38	14050.03 - 61310.11	--	1.000	1.000	1.000
**EOFAD**	8	46898.74	24886.81	5566.11 - 86473.79	1.000	--	0.745	1.000	24702.39	12389.16	8872.41 - 43017.55	1.000	--	0.862	1.000
**AOFAD**	7	72520.82	31338.13	41641.47 - 132282.33	0.592	0.745	--	1.000	45871.42	32282.46	9700.86 - 102292.73	1.000	0.862	--	1.000
**LOFAD**	8	52080.20	18881.42	31870.78 - 86457.41	1.000	1.000	1.000	--	31691.31	19028.96	10556.61 - 72701.61	1.000	1.000	1.000	--
					**SAD**							**SAD**			
**FAD**	23	56499.01	26469.46	5566.11 - 132282.33	0.368				33576.06	22941.31	8872.41 - 102292.73	0.845			

Group	N	Aβ PC			p value vs				Aβ OC			p value vs			
		Mean	SD	Range	SAD	EOFAD	AOFAD	LOFAD	Mean	SD	Range	SAD	EOFAD	AOFAD	LOFAD
**SAD**	10	25622.41	10909.18	9431.48 - 44289.94	--	1.000	1.000	0.901	23918.61	11529.16	11192.38 - 42328.44	--	1.000	1.000	1.000
**EOFAD**	8	29211.04	18660.98	5907.32 - 56031.67	1.000	--	1.000	1.000	22973.07	8579.23	9019.06 - 33484.37	1.000	--	1.000	1.000
**AOFAD**	7	34732.49	16038.82	22495.80 - 63931.73	1.000	1.000	--	1.000	25687.44	11871.92	18184.47 - 51957.73	1.000	1.000	--	1.000
**LOFAD**	8	38668.75	20145.01	12850.67 - 77723.37	0.901	1.000	1.000	--	27376.87	12667.28	11603.16 - 47608.73	1.000	1.000	1.000	--
					**SAD**							**SAD**			
**FAD**	23	34181.12	18068.92	5907.32 - 77723.37	0.225				25330.94	10793.41	9019.06 - 51957.73	0.557			

Group	N	Aβ 1-42FC			p value vs					N	sAPP TBS fraction		p value vs			
		Mean	SD	Range	SAD	EOFAD	AOFAD	LOFAD	Mean		SD	Range	SAD	EOFAD	AOFAD	LOFAD
**SAD**	10	103680.02	35775.56	59765.80 - 151362.60	--	1.000	1.000	0.098	0.99	9	0.33	0.34 - 1.39	--	0.600	1.000	0.854
**EOFAD**	8	125630.29	37799.31	67822.33 - 173095.90	1.000	--	1.000	1.000	0.74	8	0.35	0.44 - 1.47	0.600	--	0.673	1.000
**AOFAD**	7	121690.00	49958.36	80387.14 - 230689.00	1.000	1.000	--	0.928	1.12	7	0.49	0.46 - 1.81	1.000	0.673	--	0.931
**LOFAD**	8	148122.87	35426.46	90700.15 - 188479.70	0.098	1.000	0.928	--	0.76	8	0.28	0.38 - 1.10	0.854	1.000	0.931	--
					**SAD**								**SAD**			
**FAD**	23	132254.58	40959.25	67822.33 - 230689.00	0.055				0.86	23	0.40	0.38 - 1.81	0.187			

Group	N	Aβ Monomers TBS fraction			p value vs				Aβ small Oligomers TBS fraction			p value vs			
		Mean	SD	Range	SAD	EOFAD	AOFAD	LOFAD	Mean	SD	Range	SAD	EOFAD	AOFAD	LOFAD
**SAD**	9	59.23	11.42	44.47 – 81.49	--	1.000	0.084	0.017	40.77	11.42	18.51 – 55.53	--	1.000	0.084	0.017
**EOFAD**	8	53.14	7.96	40.45 – 62.03	1.000	--	0.588	0.198	46.86	7.96	37.98 – 59.56	1.000	--	0.588	0.198
**AOFAD**	7	41.46	14.44	25.59 – 65.80	0.084	0.588	--	1.000	58.54	14.44	34.19 – 74.41	0.084	0.588	--	1.000
**LOFAD**	8	38.05	12.07	18.71 – 56.76	0.017	0.198	1.000	--	61.95	12.08	43.24 – 81.29	0.017	0.198	1.000	--
					**SAD**							**SAD**			
**FAD**	23	44.33	12.98	18.71 – 65.80	*0.010*				55.67	12.98	34.20 – 81.29	*0.010*			

Group	N	Aβ 1-42			p value vs				Aβ 2 - 42			p value vs			
		Mean	SD	Range	SAD	EOFAD	AOFAD	LOFAD	Mean	SD	Range	SAD	EOFAD	AOFAD	LOFAD
**SAD**	6	44.05	31.01	22,98 - 104,70	--	0.388	*0.048*	0.233	5.80	2.11	2,88 - 9,02	--	1.000	0.111	0.641
**EOFAD**	8	19.62	12.44	2,21 - 40,33	0.388	--	1.000	1.000	4.23	2.83	1,08 - 9,35	1.000	--	1.000	1.000
**AOFAD**	7	12.19	9.55	2,38 - 26,04	*0.048*	1.000	--	1.000	2.35	1.45	,80 - 4,47	0.111	1.000	--	1.000
**LOFAD**	8	29.05	41.11	0,00 - 124,87	0.233	1.000	1.000	--	4.19	4.41	0,00 - 13,60	0.641	1.000	1.000	--
					**SAD**							**SAD**			
**FAD**	23	20.64	25.70	0,00 - 124,87	*0.010*				3.64	3.17	0,00 - 13,60	*0.046*			

Group	N	Aβ 4-42			p value vs				Aβ 5 - 42			p value vs			
		Mean	SD	Range	SAD	EOFAD	AOFAD	LOFAD	Mean	SD	Range	SAD	EOFAD	AOFAD	LOFAD
**SAD**	6	51.60	43.42	6,79 - 117,45	--	1.000	0.615	1.000	4.38	1.87	1,63 - 6,77	--	1.000	0.172	0.807
**EOFAD**	8	35.74	38.08	2,51 - 115,96	1.000	--	1.000	1.000	3.12	2.18	0,00 - 6,59	1.000	--	1.000	1.000
**AOFAD**	7	0.87	0.84	,82 - 50,31	0.615	1.000	--	1.000	4.58	2.83	0,00 - 3,95	0.172	1.000	--	1.000
**LOFAD**	8	35.77	41.85	0,00 - 124,67	1.000	1.000	1.000	--	2.75	2.62	0,00 - 6,58	0.807	1.000	1.000	--
					**SAD**							**SAD**			
**FAD**	23	30.37	34.72	0,00 - 124,67	0.178				2.61	2.15	0,00 - 6,59	0.052			

Group	N	Aβ 1-43			p value vs				Aβ Pyr 3-42			p value vs			
		Mean	SD	Range	SAD	EOFAD	AOFAD	LOFAD	Mean	SD	Range	SAD	EOFAD	AOFAD	LOFAD
**SAD**	6	1.76	0.78	1,21 - 3,18	--	1.000	1.000	1.000	10.99	3.38	5,01 - 14,85	--	1.000	0.154	0.994
**EOFAD**	8	1.55	0.86	0,00 - 2,90	1.000	--	1.000	1.000	7.82	5.41	1,75 - 16,54	1.000	--	1.000	1.000
**AOFAD**	7	18.07	20.74	0,00 - 1,74	1.000	1.000	--	1.000	1.86	1.50	1,49 - 8,15	0.154	1.000	--	1.000
**LOFAD**	8	1.33	1.64	0,00 - 4,58	1.000	1.000	1.000	--	8.05	7.34	0,00 - 22,11	0.994	1.000	1.000	--
					**SAD**							**SAD**			
**FAD**	23	1.26	1.17	0,00 - 4,58	0.357				6.91	5.58	0,00 - 22,11	0.053			

Group	N	Aβ Pyr 11-42			p value vs			
		Mean	SD	Range	SAD	EOFAD	AOFAD	LOFAD
**SAD**	6	2.32	1.00	,91 - 3,77	--	1.000	0.834	1.000
**EOFAD**	8	2.59	2.27	0,00 - 7,08	1.000	--	1.000	1.000
**AOFAD**	7	1.31	0.84	0,00 - 2,51	0.834	1.000	--	1.000
**LOFAD**	8	1.83	1.71	0,00 - 5,09	1.000	1.000	1.000	--
					**SAD**			
**FAD**	23	1.93	1.75	0,00 - 7,08	0.258			

Group	N	Aβ de novo 42/40			p value vs					Aβ de novo 38/42			p value vs				
		Mean	SD	Range	Ctrl	SAD	EOFAD	AOFAD	LOFAD	Mean	SD	Range	Ctrl	SAD	EOFAD	AOFAD	LOFAD
**Ctrl**	5	0.13	0.02	0.11–0.16	--	1.000	0.119	0.051	0.263	4.65	0.63	3.88 – 5.37	--	1.000	*0.031*	*0.022*	0.063
**SAD**	5	0.10	0.02	0.07–0.12	1.000	--	*0.008*	*0.003*	*0.023*	4.60	2.55	1.98 – 8.71	1.000	--	0.611	0.452	0.998
**EOFAD**	8	0.19	0.03	0.16 –0.25	*0.119*	*0.008*	--	1.000	1.000	2.56	0.29	2.07 – 2.96	*0.031*	1.000	--	1.000	1.000
**AOFAD**	7	0.21	0.04	0.14 –0.27	0.051	*0.003*	1.000	--	1.000	2.50	0.60	1.69 – 3.40	*0.022*	1.000	1.000	--	1.000
**LOFAD**	8	0.19	0.03	0.16 –0.26	0.263	*0.023*	1.000	1.000	--	2.70	0.58	1.94 – 3.76	0.063	1.000	1.000	1.000	--
					**Ctrl**	**SAD**							**Ctrl**	**SAD**			
**FAD**	23	0.20	0.03	0.14 –0.27	*0.011*	*0.000*				2.59	0.49	1.69 – 3.76	*0.002*	0.098			

Table 7. pTau associated pathology according Age of Onset in PSEN1 E280A FAD

Group	N	pTau FC			p value vs				pTau TC			p value vs			
		Mean	SD	Range	SAD	EOFAD	AOFAD	LOFAD	Mean	SD	Range	SAD	EOFAD	AOFAD	LOFAD
**SAD**	10	129774.95	55378.25	50758.78 - 220860.24	--	1.000	0.294	1.000	157137.27	48793.64	75932.61 - 235730.10	--	1.000	1.000	0.153
**EOFAD**	8	146879.89	64768.40	31032.00 - 233794.30	1.000	--	0.959	0.500	144414.17	64410.79	10844.79 - 205814.82	1.000	--	1.000	0.315
**AOFAD**	7	185869.87	32529.29	142686.92 - 247643.68	0.294	0.959	--	*0.013*	193653.67	44215.76	146645.03 - 264019.15	1.000	1.000	--	*0.009*
**LOFAD**	8	91023.18	53368.54	13630.87 - 181075.44	1.000	0.500	*0.013*	--	97869.97	42377.17	12240.99 - 166515.37	0.153	0.315	*0.009*	--
					**SAD**							**SAD**			
**FAD**	23	139317.99	63937.85	13630.87 - 247643.68	0.667				143210.82	63105.80	10844.79 - 264019.15	0.557			

Group	N	pTau PC			p value vs				pTau OC			p value vs			
		Mean	SD	Range	SAD	EOFAD	AOFAD	LOFAD	Mean	SD	Range	SAD	EOFAD	AOFAD	
**SAD**	10	134904.72	42389.12	56254.06 - 188475.73	--	1.000	0.148	0.433	159866.20	62585.12	104677.40 - 306055.14	--	1.000	0.689	1.000
**EOFAD**	8	127870.86	79256.52	9174.50 - 44583.56	1.000	--	0.108	0.844	157183.07	86851.81	19585.98 - 263194.05	1.000	--	1.000	0.844
**AOFAD**	7	195971.69	31990.72	172630.77 - 262057.36	0.148	0.108	--	*0.001*	204415.26	39780.00	144712.13 - 241160.45	0.689	1.000	--	0.067
**LOFAD**	8	81328.62	45523.03	27188.48 - 145267.99	0.433	0.844	*0.001*	--	106925.05	79290.51	15880.13 - 246252.19	1.000	0.844	0.067	--
					**SAD**							**SAD**			
**FAD**	23	132408.59	71965.25	9174.50 - 262057.36	1.000				154077.04	80314.00	15880.13 - 263194.05	0.754			

Group	N	Total Tau TBS			p value vs				Total Tau FA			p value vs			
		Mean	SD	Range	SAD	EOFAD	AOFAD	LOFAD	Mean	SD	Range	SAD	EOFAD	AOFAD	
**SAD**	9	5.73	1.25	4.69 - 8.65	--	*0.002*	0.355	1.000	7.79	2.94	3.92 - 12.24	--	1.000	0.268	1.000
**EOFAD**	8	11.21	2.52	7.17 - 14.83	*0.002*	--	0.779	*0.000*	9.73	3.65	5.67 - 14.27	1.000	--	1.000	0.373
**AOFAD**	7	7.97	1.21	5.78 - 9.45	0.355	0.779	--	0.086	10.69	2.69	8.33 - 13.93	0.268	1.000	--	*0.033*
**LOFAD**	8	4.64	2.17	1.53 - 7.24	1.000	*0.000*	0.086	--	6.00	2.31	2.20 - 8.73	1.000	0.373	*0.033*	--
					**SAD**							**SAD**			
**FAD**	23	7.94	3.43	1.53 - 14.83	*0.046*				8.72	3.50	2.20 - 14.27	0.390			

Group	N	S400 pTau / Total Tau TBS			p value vs				S400 pTau / Total Tau FA			p value vs			
		Mean	SD	Range	SAD	EOFAD	AOFAD	LOFAD	Mean	SD	Range	SAD	EOFAD	AOFAD	LOFAD
**SAD**	9	1.44	0.61	0.60–2.30	--	0.156	0.038	1.000	0.72	0.33	0.39–1.26	--	0.994	1.000	1.000
**EOFAD**	8	0.87	0.22	0.39 –1.15	0.156	--	1.000	0.151	0.53	0.16	0.37–0.87	0.994	--	1.000	0.311
**AOFAD**	7	0.71	0.37	0.36–1.25	0.038	1.000	--	*0.038*	0.65	0.15	0.45–0.89	1.000	1.000	--	1.000
**LOFAD**	8	1.29	0.23	0.85–1.66	1.000	0.151	*0.038*	--	0.75	0.25	0.35–1.06	1.000	0.311	1.000	--
					**SAD**							**SAD**			
**FAD**	23	0.93	0.33	0.36 - 1.66	*0.042*	--	--	--	0.64	0.21	0.35 - 1.06	0.722	--	--	--

Group	N	Synaptophysin Particles Density			p value vs			Synaptophysin Particles Size			p value vs		
		Mean	SD	Range	EOFAD	AOFAD	LOFAD	Mean	SD	Range	EOFAD	AOFAD	LOFAD
**EOFAD**	5	13795	12102	2085 – 32027	--	*0.036*	0.283	0.27	0.05	0.22 – 0.35	--	0.480	0.957
**AOFAD**	5	48003	15015	27159 – 68421	*0.036*	--	0.175	0.30	0.02	0.27 – 0.32	0.480	--	0.646
**LOFAD**	5	26787	25135	2524 – 68647	0.283	0.175	--	0.28	0.04	0.22 – 0.32	0.957	0.646	--
**FAD**	15	29528	17417	2085 – 68647				0.28	0.04	0.22 – 0.35			

Table 8. pTau pathology comparison E-AOFAD vs LOSAD in PSEN1 E280A FAD

Subgroup	N	pTau FC			p value vs		pTau TC			p value vs	
		Mean	SD	Range	SAD	LOFAD	Mean	SD	Range	SAD	LOFAD
**E-AOFAD**	15	165075.21	54372.41	31032.00 - 247643.68	0.461	*0.018*	167392.60	59655.57	10844.79 - 264019.15	1.000	*0.010*

Subgroup	N	pTau PC			p value vs		pTau OC			p value vs	
		Mean	SD	Range	SAD	LOFAD	Mean	SD	Range	SAD	LOFAD
**E-AOFAD**	15	159651.25	69398.32	9174.50 - 262057.36	0.795	*0.009*	179224.76	71026.14	19585.98 - 263194.05	0.753	0.065

Subgroup	N	Total Tau TBS			p value vs		Total Tau FA			p value vs	
		Mean	SD	Range	SAD	LOFAD	Mean	SD	Range	SAD	LOFAD
**E-AOFAD**	15	9.70	2.57	5.78 - 14.83	*0.001*	*0.001*	10.18	3.16	5.67 - 14.27	0.079	*0.016*

Subgroup	N	S400 pTau / Total TBS			p value vs		S400 pTau / Total FA			p value vs	
		Mean	SD	Range	SAD	LOFAD	Mean	SD	Range	SAD	LOFAD
**E-AOFAD**	15	0.78	0.28	0.36 - 1.25	*0.008*	*0.003*	0.58	0.16	0.34 - 0.89	0.421	0.213

E-AOFAD = Early and average age of onset Familial AD

Table 9. pTau-related Kinases levels according Age of Onset in PSEN1 E280A FAD

Group	N	AKT			p value vs				pAKT			p value vs			
		Mean	SD	Range	Ctrl	SAD	E-AOFAD	LOFAD	Mean	SD	Range	Ctrl	SAD	E-AOFAD	LOFAD
Ctrl	5	0.32	0.66	-0.48–1.22	--	0.622	0.117	0.304	-0.35	0.51	-0.68 – 0.51	--	1.000	1.000	0.270
SAD	10	1.28	1.63	-1.16–3.77	0.622	--	1.000	1.000	-0.35	0.76	-0.78 – 1.77	1.000	--	1.000	*0.028*
E-AOFAD	15	1.99	1.82	0.00–7.08	0.117	1.000	--	1.000	0.02	1.22	-0.77 – 3.69	1.000	1.000	--	0.131
LOFAD	8	1.83	1.71	0.00–5.09	0.304	1.000	1.000	--	0.62	0.85	-0.30 – 2.19	0.270	*0.028*	0.131	--
					Ctrl	SAD						Ctrl	SAD		
FAD	23	1.93	1.75	0.00–7.08	0.438	0.875			0.23	1.12	-0.77 – 3.69	0.131	0.352		

Group	N	pAKT Ratio			p value vs				GSK3β basal			p value vs			
		Mean	SD	Range	Ctrl	SAD	E-AOFAD	LOFAD	Mean	SD	Range	Ctrl	SAD	E-AOFAD	LOFAD
Ctrl	5	-0.48	0.20	-0.62 - -0.14	--	1.000	1.000	*0.038*	0.88	0.67	0.21 – 1.52	--	0.213	0.841	*0.017*
SAD	10	-0.42	0.33	-0.66 – 0.29	1.000	--	1.000	*0.006*	-0.24	0.63	-1.32 – 0.47	0.213	--	1.000	1.000
E-AOFAD	15	-0.13	0.68	-0.67 – 1.52	1.000	1.000	--	0.079	0.25	1.22	-1.30 – 2.38	0.841	1.000	--	0.187
LOFAD	8	1.06	1.57	-0.25 – 4.50	*0.038*	*0.006*	0.079	--	-0.71	0.51	-1.35 – 0.24	*0.017*	1.000	0.187	--
					Ctrl	SAD						Ctrl	SAD		
FAD	23	0.29	1.19	-0.67 – 4.50	0.06	0.06			-0.087	1.11	-1.30 – 2.38	0.08	0.88		

Group	N	pGSK3βS9			p value vs				pGSK3βS9 Ratio			p value vs			
		Mean	SD	Range	Ctrl	SAD	E-AOFAD	LOFAD	Mean	SD	Range	Ctrl	SAD	E-AOFAD	LOFAD
Ctrl	5	0.12	0.50	-0.43 – 0.68	--	1.000	1.000	1.000	-0.47	0.12	-0.67 - -038	--	1.000	1.000	0.319
SAD	10	-0.32	0.58	-1.05 – 0.90	1.000	--	1.000	0.881	-0.40	0.26	-0.91 – -0.00	1.000	--	1.000	0.496
E-AOFAD	15	0.17	1.45	-1.04 – 3.97	1.000	1.000	--	1.000	0.13	1.21	-0.88 – 2.44	1.000	1.000	--	0.313
LOFAD	8	0.01	0.56	-0.95 – 0.92	1.000	0.881	1.000	--	0.54	1.24	-0.70 – 2.73	0.319	0.496	0.313	--
					Ctrl	SAD						Ctrl	SAD		
FAD	23	0.11	1.20	-1.04 -3.97	0.610	0.392			0.28	1.20	-0.88 – 2.73	0.484	0.484		

Group	N	pGSK3βY216			p value vs				pGSK3βY216 Ratio			p value vs			
		Mean	SD	Range	Ctrl	SAD	E-AOFAD	LOFAD	Mean	SD	Range	Ctrl	SAD	E-AOFAD	LOFAD
**Ctrl**	5	1.48	0.65	0.90 -2.46	--	*0.026*	*0.014*	0.112	0.41	0.17	0.21 – 0.60	--	0.367	0.133	1.000
**SAD**	10	-0.29	0.80	-1.22 – 1.11	*0.026*	--	1.000	1.000	-0.31	0.74	-1.23 – 0.99	0.367	--	1.000	0.151
**E-AOFAD**	15	-0.16	1.06	-1.17 – 2.46	*0.014*	1.000	--	1.000	-0.33	1.06	-1.19 – 2.96	0.133	1.000	--	*0.033*
**LOFAD**	8	-0.26	0.39	-0.66 – 0.36	0.112	1.000	1.000	--	0.76	1.09	-0.34 – 2.67	1.000	0.151	*0.033*	--
					**Ctrl**	**SAD**						**Ctrl**	**SAD**		
**FAD**	23	-0.20	0.88	-1.17 – 2.46	*0.005*	0.754			0.05	1.17	-1.19 – 2.96	0.224	0.411		

Group	N	MEK			p value vs				pMEK			p value vs			
		Mean	SD	Range	Ctrl	SAD	E-AOFAD	LOFAD	Mean	SD	Range	Ctrl	SAD	E-AOFAD	LOFAD
**Ctrl**	5	0.07	0.66	-0.720 – 1.10	--	1.000	1.000	1.000	0.32	1.66	-1.13 – 3.08	--	1.000	1.000	1.000
**SAD**	10	-0.31	0.84	-1.09 – 1.55	1.000	--	1.000	0.157	-0.14	0.94	-1.11 – 1.87	1.000	--	1.000	1.000
**E-AOFAD**	15	-0.07	1.33	-1.17 – 4.21	1.000	1.000	--	0.079	0.11	0.97	-1.08 – 2.12	1.000	1.000	--	1.000
**LOFAD**	8	0.47	0.48	-0.13 – 1.28	1.000	0.157	0.079	--	-0.23	0.72	-1.13 – 0.95	1.000	1.000	1.000	--
					**Ctrl**	**SAD**						**Ctrl**	**SAD**		
**FAD**	23	0.12	1.12	-1.17 – 4.21	0.787	0.778			-0.01	0.89	-1.13 – 2.12	1.114	1.000		

Group	N	pMEK Ratio			p value vs				ERK1/2			p value vs			
		Mean	SD	Range	Ctrl	SAD	E-AOFAD	LOFAD	Mean	SD	Range	Ctrl	SAD	E-AOFAD	LOFAD
**Ctrl**	5	-0.38	0.15	-0.55 - -0.18	--	0.725	0.425	0.725	0.06	0.23	-0.18 – 0.38	--	1.000	1.000	1.000
**SAD**	10	-0.07	0.47	-0.56 – 0.71	0.725	--	0.725	0.378	-0.42	0.90	-1.41 – 1.25	1.000	--	1.000	0.129
**E-AOFAD**	15	0.42	1.46	-0.51 – 4.29	0.425	0.725	--	*0.022*	-0.17	0.95	-1.58 – 1.11	1.000	1.000	--	0.329
**LOFAD**	8	-0.47	0.09	-0.59 - -0.36	0.725	0.378	*0.022*	--	0.81	1.15	-0.91 – 2.61	1.000	0.129	0.329	--
					**Ctrl**	**SAD**						**Ctrl**	**SAD**		
**FAD**	23	0.11	1.24	-0.59 -4.29	1.058	1.000			0.17	1.11	-1.58 – 2.61	0.697	0.353		

Group	N	pERK1/2			p value vs				pERK1/2 Ratio			p value vs			
		Mean	SD	Range	Ctrl	SAD	E-AOFAD	LOFAD	Mean	SD	Range	Ctrl	SAD	E-AOFAD	LOFAD
**Ctrl**	5	-0.12	0.66	-0.64 – 0.96	--	1.000	1.000	0.785	-0.30	0.51	-0.70 – 0.53	--	1.000	1.000	0.440
**SAD**	10	0.15	1.43	-0.75 – 3.98	1.000	--	1.000	0.621	0.47	1.44	-0.65 - 3.17	1.000	--	1.000	*0.019*
**E-AOFAD**	15	0.27	0.94	-0.90 – 2.23	1.000	1.000	--	0.199	0.16	0.86	-0.71 – 1.96	1.000	1.000	--	*0.003*
**LOFAD**	8	-0.62	0.17	-0.84 - -0.28	0.785	0.621	0.199	--	-0.70	0.10	-0.84 - -0.50	0.440	*0.019*	*0.003*	--
					**Ctrl**	**SAD**						**Ctrl**	**SAD**		
**FAD**	23	-0.04	0.87	-0.90 – 2.23	1.393	1.000			-0.14	0.80	-0.84 – 1.96	0.928	0.580		

Group	N	CDK5			p value vs				Fyn			p value vs			
		Mean	SD	Range	Ctrl	SAD	E-AOFAD	LOFAD	Mean	SD	Range	Ctrl	SAD	E-AOFAD	LOFAD
**Ctrl**	5	0.62	1.04	-0.61 – 2.01	--	0.167	1.000	1.000	0.51	0.38	-0.00 – 1.04	--	0.169	0.293	1.000
**SAD**	10	-0.62	0.62	-1.31 – 0.34	0.167	--	0.716	0.207	-0.72	0.16	-0.85 - -0.43	0.169	--	1.000	*0.001*
**E-AOFAD**	15	0.07	1.21	-1.37 – 2.40	1.000	0.716	--	1.000	-0.36	0.95	-0.85 – 1.76	0.293	1.000	--	*0.002*
**LOFAD**	8	0.25	0.58	-0.75 – 1.17	1.000	0.207	1.000	--	1.27	0.55	0.49 – 2.21	1.000	*0.001*	*0.002*	--
					**Ctrl**	**SAD**						**Ctrl**	**SAD**		
**FAD**	23	0.13	1.03	-1.37 – 2.40	0.384	0.091			0.20	1.14	-0.85 – 2.21	0.607	0.208		

Group	N	mPPA2			p value vs				CamKIIa			p value vs			
		Mean	SD	Range	Ctrl	SAD	E-AOFAD	LOFAD	Mean	SD	Range	Ctrl	SAD	E-AOFAD	LOFAD
**Ctrl**	5	-0.04	0.36	-0.33 – 0.55	--	1.000	1.000	0.607	-0.32	0.93	-1.32 – 0.94	--	1.000	1.000	0.193
**SAD**	10	-0.47	0.63	-1.02 – 0.36	1.000	--	1.000	*0.006*	-0.49	0.68	-1.37 – 0.44	1.000	--	1.000	*0.016*
**E-AOFAD**	15	-0.24	1.19	-1.02 – 2.47	1.000	1.000	--	*0.006*	-0.09	1.03	-1.48 – 1.84	1.000	1.000	--	0.064
**LOFAD**	8	1.07	0.34	0.59 – 1.69	0.607	*0.006*	*0.006*	--	0.99	0.74	0.02 – 2.19	0.193	*0.016*	0.064	--
					**Ctrl**	**SAD**						**Ctrl**	**SAD**		
**FAD**	23	0.21	1.16		0.976	0.191			0.28	1.06		0.294	0.143		

Group	N	pCamKIIa			p value vs				pCamKIIa Ratio			p value vs			
		Mean	SD	Range	Ctrl	SAD	E-AOFAD	LOFAD	Mean	SD	Range	Ctrl	SAD	E-AOFAD	LOFAD
**Ctrl**	5	0.36	0.75	-0.45 – 1.13	--	0.976	0.420	1.000	0.80	1.42	-0.33 – 2.92		1.000	0.223	0.166
**SAD**	10	-0.34	0.72	-1.11 – 1.50	0.976	--	1.000	0.171	0.03	0.65	-0.79 – 0.93	1.000		1.000	1.000
**E-AOFAD**	15	-0.28	1.16	-1.37 – 1.98	0.420	1.000	--	*0.034*	-0.05	1.20	-1.13 – 3.62	0.223	1.000		1.000
**LOFAD**	8	0.72	0.78	-0.31 – 2.10	1.000	0.171	*0.034*	--	-0.45	0.08	-0.57 - -0.35	0.166	1.000	1.000	
					**Ctrl**	**SAD**						**Ctrl**	**SAD**		
**Total FAD**	23	0.07	1.13	-1.37 – 2.10	0.769	0.769			-0.19	0.98	-1.13 – 3.62	*0.036*	0.264		

Group	N	JNK			p value vs				pJNK			p value vs			
		Mean	SD	Range	Ctrl	SAD	E-AOFAD	LOFAD	Mean	SD	Range	Ctrl	SAD	E-AOFAD	LOFAD
**Ctrl**	5	-0.08	0.67	-0.85 – 0.65	--	1.000	1.000	0.425	-0.16	0.55	-0.51 –0.81		1.000	1.000	1.000
**SAD**	10	-0.01	0.62	-1.21 – 1.09	1.000	--	1.000	0.055	-0.33	0.76	-1.19 – 1.32	1.000		1.000	1.000
**E-AOFAD**	15	0.41	1.34	-1.15 – 3.27	1.000	1.000	--	*0.021*	0.26	1.36	-1.02 – 3.26	1.000	1.000		1.000
**LOFAD**	8	-0.71	0.15	-0.88 - -0.46	0.425	0.055	*0.021*	--	0.02	0.63	-0.58 – 1.46	1.000	1.000	1.000	
					**Ctrl**	**SAD**						**Ctrl**	**SAD**		
**Total FAD**	23	0.02	1.20	-1.15 – 3.27	0.545	0.741			0.18	1.14	-1.02 – 3.26	0.928	0.480		

Group	N	pJNK Ratio			p value vs			
		Mean	SD	Range	Ctrl	SAD	E-AOFAD	LOFAD
**Ctrl**	5	-0.25	0.67	-0.92 – 0.61	--	1.000	1.000	1.000
**SAD**	10	-0.32	1.02	-1.05 – 2.13	1.000	--	1.000	0.272
**E-AOFAD**	15	0.05	1.23	-1.17 – 2.31	1.000	1.000	--	0.735
**LOFAD**	8	0.45	0.49	-0.00 – 1.23	1.000	0.272	0.735	--
					**Ctrl**	**SAD**		
**Total FAD**	23	0.19	1.04	-1.17 – 2.31	0.529	0.420		

Table 10. Novel significant uncommon variants after FDR and Major Haplotype analysis

Chr	Position	RsID	Gene	Reference	F-Test P	F-Test FDR	Major Allele	Major Allele Frequency	Dependent Average for DD	Dependent Average for DD	Dependent Average for DD
11	7717205		OVCH2	A	3.28E-05	0.045081044	A	0.642857143	57.6	?	46.88888889
21	11138217	rs4041711	?	G	0.000209775	0.144220563	G	0.785714286	41.66666667	?	53.18181818
12	22839785		ETNK1	A	0.00045173	0.207042897	A	0.821428571	37	43.66666667	54.2
17	78234611		RNF213	CGGCGG	0.000642249	0.220773021	CGGCGG	0.571428571	55.83333333	?	46.875
10	104235832		TMEM180	G	0.001049905	0.288723944	G	0.714285714	57.5	?	48
11	7717209		OVCH2	A	0.0012204	0.239721494	G	0.714285714	44	?	53.4
11	7717212		OVCH2	A	0.0012204	0.239721494	T	0.714285714	44	?	53.4
11	7717213		OVCH2	A	0.001407946	0.241990695	T	0.785714286	42.66666667	?	52.90909091
16	3452453		ZSCAN3	C	0.001650996	0.252235545	C	0.785714286	60	56	47.8
14	23300314		SLC7A7	G	0.001755451	0.219431362	G	0.714285714	57.25	?	48.1
19	50643184		?	G	0.001755451	0.219431362	G	0.714285714	57.25	?	48.1
8	120818705		TAF2	C	0.002036554	0.233355174	C	0.678571429	44	46.33333333	54.875
15	77092481		SCAPER	A	0.002423034	0.256282411	A	0.785714286	43	?	52.81818182
19	871891		MED16	C	0.002960535	0.290766859	C	0.642857143	45.4	?	53.66666667
14	23300315		SLC7A7	C	0.003257921	0.298642785	C	0.571428571	55.4	54.5	46.28571429
7	21892043		DNAH11	T	0.003773076	0.324248749	T	0.821428571	39.5	54	52.45454545
19	44645602		ZNF234	T	0.003773726	0.305227845	T	0.75	44.66666667	40	53.6
21	9911602	rs79513382	TEKT4P2	A	0.003984601	0.304379276	G	0.5	46.85714286	?	54.57142857
6	116837849		TRAPPC3	T	0.004605535	0.301552888	T	0.785714286	58	?	48.72727273
9	136890390		LINC00094	-	0.004605535	0.301552888	-	0.785714286	58	?	48.72727273

Chr	Position	Identifier	Gene	Reference	Block #	# Haplotypes	# Haps Regressed	Full-Model P-Value
17	21320855	rs76059352	KCNJ12	A	1050	4	3	0.001448249
19	44096026		IRGQ	C	1202	4	3	0.00155688
12	22839785		ETNK1	A	761	4	3	0.00568534
11	7716924		OVCH2	G	649	4	3	0.005779731
16	456238		NME4	A	965	3	2	0.005891017
17	76808806	rs377310938	USP36	G	1104	4	3	0.007424818
8	31497267		NRG1	T	503	4	3	0.009874149

Table 11. Top 20 Known uncommon variants, functional mutations

Marker	Chr	Position	Identifier	Gene	Ref	F-Test P	F-Test FDR	Major Allele	Major Allele Frequency	Dependent Average for DD	Dependent Average for DD	Dependent Average for DD
4:70807771-SNV	4	70807771	rs10030475	CSN1S1	C	2.94E-05	0.16653829	C	0.642857143	43.25	46	55.625
9:86278817-SNV	9	86278817	rs7866234	UBQLN1	C	0.000145619	0.412174602	C	0.785714286	38.5	47.5	53.8
22:26932150-SNV	22	26932150	rs3752523	TPST2	G	0.000213365	0.402618889	G	0.714285714	43.25	?	53.7
8:99030280-SNV	8	99030280	rs2248014	MATN2	T	0.000226322	0.320301609	C	0.642857143	42	48	56
10:70992664-SNV	10	70992664	rs874556	HKDC1	C	0.000231071	0.261618347	C	0.75	38	48.66666667	54.22222222
5:111545670-SNV	5	111545670	rs890757	EPB41L4A	C	0.000297684	0.280864477	G	0.785714286	43	39.5	54.5
22:42537196-SNV	22	42537196	rs56404506	CYP2D7P	T	0.000334784	0.270744991	T	0.714285714	58	?	47.8
1:248789504-SNV	1	248789504	rs1892442	OR2T11	T	0.000416507	0.294730536	C	0.785714286	42	?	53.09090909
1:46493460-SNV	1	46493460	rs1707336	MAST2	T	0.000475257	0.298936825	G	0.571428571	38.66666667	52.33333333	56
2:202252539-SNV	2	202252539	rs2244438	TRAK2	G	0.000828321	0.468912785	G	0.571428571	58.66666667	52.83333333	43.4
15:23688944-SNV	15	23688944	rs4778531	GOLGA6L2	A	0.000867063	0.446222191	A	0.535714286	55.5	54	46.14285714
14:24505722-SNV	14	24505722	rs8005834	DHRS4L1	C	0.000937894	0.442451337	C	0.607142857	57.25	53	46
6:31324931-SNV	6	31324931	rs151341074	HLA-B	A	0.001284419	0.559314917	C	0.607142857	43.5	50.66666667	54.85714286
1:216348764-SNV	1	216348764	rs1805049	USH2A	C	0.001345451	0.476037471	T	0.571428571	58.66666667	52.5	43.8
2:234469664-SNV	2	234469664	rs6753062	USP40	T	0.001345451	0.476037471	T	0.571428571	55	58.5	45.42857143
7:100371114-SNV	7	100371114	rs314298	ZAN	C	0.001345451	0.476037471	C	0.571428571	57	52.75	45.16666667
15:33359370-SNV	15	33359370	rs11072170	FMN1	C	0.001356147	0.451596936	T	0.5	41	53.83333333	55.75
12:68707287-SNV	12	68707287	rs2306392	MDM1	G	0.001407946	0.442798973	G	0.785714286	37	45.5	54.55555556
8:18258316-SNV	8	18258316	rs1208	NAT2	G	0.001492495	0.444684879	A	0.642857143	38	49.83333333	55.83333333
8:37699195-SNV	8	37699195	rs4976898	GPR124	C	0.00157698	0.4463643	G	0.714285714	42	50.5	53.66666667

Table 12. Top 20 common variants, functional mutations

Marker	Chr	Position	Identifier	Gene	Reference	Alternates	#Alleles (AN)	Ref/Alt	P-Values	FDR	Minor Allele D Frequency	Major Allele d Frequency
14:73957772-SNV	14	73957772	rs10144469	HEATR4	A	G	2	A/G	1.78E-76	1.64E-73	0.42307692	0.57692308
6:46672943-SNV	6	46672943	rs1051931	PLA2G7	A	G	2	A/G	1.79E-76	8.26E-74	0.07692308	0.92307692
18:70417396-SNV	18	70417396	rs922999	NETO1	C	T	2	C/T	1.79E-76	5.51E-74	0.07142857	0.92857143
7:151680072-SNV	7	151680072	rs6960270	GALNTL5	T	C	2	T/C	6.56E-11	1.51E-08	0.10714286	0.89285714
10:62551889-SNV	10	62551889	rs2456777	CDK1	A	G	2	A/G	1.01E-09	1.86E-07	0.15384615	0.84615385
11:5410934-SNV	11	5410934	rs1498467	OR51M1	T	G	2	T/G	7.56E-08	1.16E-05	0.26923077	0.73076923
21:46057393-SNV	21	46057393	rs2838602	TSPEAR	T	A	2	T/A	3.00E-07	3.95E-05	0.19230769	0.80769231
16:1820992-SNV	16	1820992	rs11890	NME3	T	A	2	T/A	4.17E-05	0.00480845	0.10714286	0.89285714
9:94486321-SNV	9	94486321	rs10761129	ROR2	C	T	2	C/T	0.00067952	0.06961299	0.32142857	0.67857143
X:31496350-SNV	X	31496350	rs1800280	DMD	C	T	2	C/T	0.00648595	0.59800426	0.07692308	0.92307692
17:66449122-SNV	17	66449122	rs883541	WIPI1	G	A	2	G/A	0.0123	1	0.15384615	0.84615385
18:76753588-SNV	18	76753588	rs7240860	SALL3	A	G	2	A/G	0.0123	0.94505023	0.21428571	0.78571429
2:223436607-SNV	2	223436607	rs7185	FARSB	C	T	2	C/T	0.01284356	0.91090502	0.03571429	0.96428571
3:195515617-SNV	3	195515617	rs2641776	MUC4	G	C	2	G/C	0.01284356	0.84584038	0.03571429	0.96428571
4:40810747-SNV	4	40810747	rs2261167	NSUN7	A	G	2	A/G	0.01284356	0.78945102	0.03846154	0.96153846
11:112065434-SNV	11	112065434	rs10891338	BCO2	T	C	2	T/C	0.01284356	0.74011033	0.03571429	0.96428571
15:67457335-SNV	15	67457335	rs1065080	SMAD3	A	G	2	A/G	0.01284356	0.69657443	0.03571429	0.96428571
16:72042682-SNV	16	72042682	rs3213422	DHODH	A	C	2	A/C	0.01321707	0.67700747	0.32142857	0.67857143
19:44471209-SNV	19	44471209	rs365745	ZNF221	T	A	2	T/A	0.02315345	1	0.10714286	0.89285714

Table 13. Protein-protein interactions of identified genes

Chr	Gene	Name	Network	with UBC	Others
1	MAST2	Microtubule-associated serine/threonine-protein kinase 2	1	no	with APP, reconstituted complex, with GCN1L1, affinity capture
1	OR2T11	Olfactory receptor 2T11	0	no	
1	USH2A	Usherin	1	no	
2	FARSB	Phenylalanyl-tRNAsynthetase beta subunit	1		affinity capture
2	TRAK2	Trafficking kinesin-binding protein 2	1	no	with ELAVL1, affinity capture, to UBC. With KCNJ2 and GSK3B
2	USP40	Ubiquitin carboxyl-terminal hydrolase 40	1		affinity capture
3	MUC4	Mucin 4, cell surface associated	0	no	
4	CSN1S1	Alpha-S1-casein	1	no	with PLK1, biochemical activity, to UBC and APP
4	NSUN7	NOP2/Sun RNA methyltransferase family member 7	0	no	
5	EPB41L4A	Erythrocyte membrane protein band 4.1 like 4A	1	no	with APP, reconstituted com
6	HLA-B	Major histocompatibility complex, class I, B	1		affinity capture
6	PLA2G7	Phospolipase A2 group VII	1		affinity capture
6	TRAPPC3	Trafficking protein particle complex subunit 3	1	affinity capture	with APP, reconstituted complex
7	DNAH11	Dynein heavy chain 11, axonemal	1		affinity capture
7	GALNTL5	Polypeptide N-acetylgalactosaminyltransferase like 5	0	no	
7	ZAN	Zonadhesin	0	no	
8	GPR124	G-protein coupled receptor 124	0	no	with DLG1, several methods, to UBC
8	MATN2	Matrilin-2	0	no	
8	NAT2	Arylamine N-acetyltransferase 2	1	no	with APP, reconstituted complex
8	NRG1	Pro-neuregulin-1, membrane-bound isoform	1	no	With LIMK1, several methods, to UBC
8	TAF2	Transcription initiation factor TFIID subunit 2	1		affinity capture
9	ROR2	Receptor tyrosine kinase like orphan receptor 2	1		affinity capture
9	UBQLN1	Ubiquilin 1	1	several methods, hit and bait	FRET as bait, PSEN1, hit complex, two hybrid (see others)
10	CDK1	Cyclin dependent kinase 1	1		affinity capture, several methods
10	HKDC1	Hexokinase domain-containing protein 1	1		affinity capture
10	TMEM180	Transmembrane protein 180	1	affinity capture	with APP, reconstituted complex
11	BCO2	Beta-carotene oxygenase 2	0	no	
11	OR51M1	Olfactory receptor family 51 subfamily M member 1	0	no	
11	OVCH2	Ovochymase-2	0	no	
12	ETNK1	Ethanolamine kinase 1	1	affinity capture	with UBQL1, two hybrid
12	MDM1	Nuclear protein MDM1	0	no	with HDAC8, affinity capture, to UBC
12	UBC	Polyubiquitin-C	1	-----	With Tau, several methods

14	DHRS4L1	Putative dehydrogenase/reductase SDR family member 4-like 2	1		affinity capture
14	HEATR4	HEAT repeat containing 4	0	no	
14	PSEN1	Presenilin-1	1	several methods, hit and bait	With Tau, co-fractionation
14	SLC7A7	Solute carrier family 7 member 7	0	no	
15	FMN1	Formin-1	1	no	with PRPF40A, bait, protein-peptide, to UBC
15	GOLGA6L2	Golgin subfamily A member 6-like protein 2	0	no	
15	SCAPER	S phase cyclin A-associated protein in ER	1		affinity capture
15	SMAD3	SMAD family member 3	1		anti tagcoimmunoprecipitation
16	DHODH	Dihydroorotate dehydrogenase (quinone)	1		affinity capture
16	NME3	NME/NM23 nucleoside diphosphate kinase 3	1	affinity capture	With APP, reconstituted complex
16	NME4	Nucleoside diphosphate kinase, mitochondrial	1		affinity capture
16	ZSCAN3	Zinc finger protein 24	0	no	
17	KCNJ12	ATP-sensitive inward rectifier potassium channel 12	1		affinity capture
17	MAPT	Microtubule-associated protein Tau	1	several methods, hit and bait	with APP, reconstituted complex, with PSEN1, co-fractionation
17	RNF213	E3 ubiquitin-protein ligase RNF213	1		affinity capture
17	USP36	Ubiquitin carboxyl-terminal hydrolase 36	1		affinity capture
17	WIPI1	WD repeat domain, phosphoinositide interacting 1	0	no	
18	NETO1	Neuropilin and tolloid like 1	0	no	
18	SALL3	Spalt like transcription factor 3	1		affinity capture
19	IRGQ	Immunity-related GTPase family Q protein	1	no	With GABARAPL2, affinity capture, to UBC
19	MED16	Mediator complex subunit MED16	1		affinity capture
19	ZNF221	Zinc finger protein 221	0	no	
19	ZNF234	Zinc Finger Transcription Factor 234	0	no	With ELAVL1, affinity capture, to UBC.
21	APP	Amyloid beta A4 protein	1	affinity capture	With Tau, reconstituted complex
21	TEKT4P2	Tektin 4 pseudogene 2	0	no	
21	TSPEAR	Thrombospondin type laminin G domain and EAR repeats	0	no	
22	CYP2D7P	Cytochrome P450, family 2, subfamily D, polypeptide 7 pseudogene 1	0	no	
22	TPST2	Protein-tyrosine sulfotransferase 2	0	no	
X	DMD	Dystrophin, muscular dystrophy	1	affinity capture	With APP, reconstituted complex

Table 14. Significant biological pathways from protein network

Pathway GO:BP	Total	Expected	Hits	p Value	FDR	Proteins
Regulation of protein metabolic process	1820	5.62	15	0.000225	0.044	UBC, DMD, UBQLN1, PSEN1, SUMO1, APP, MAST2, SMAD3, BRCA1, CDK1, ELAVL1, GABARAPL2, CUL1, NEDD4L, DLG1
Protein catabolic process	644	1.98	10	1.99E-05	0.0163	UBC, PSEN1, SUMO1, SMAD3, CDK1, GABARAPL2, CUL1, NEDD4L, USP36, USP40
Neuron development	945	2.91	10	0.00048	0.0492	UBC, DMD, MAPT, APP, PSEN1, CDK1, DLG1, SALL3, COL1A1, MATN2
Positive regulation of cellular component organization	560	1.72	9	4.25E-05	0.0174	DMD, SUMO1, NEDD4L, DLG1, SMAD3, BRCA1, MAPT, FMN1, COL1A1
Protein modification by small protein conjugation	713	2.2	9	0.000268	0.044	UBC, UBQL1, PSEN1, SUMO1, CDK1, BRCA1, CUL1, NEDD4L, RNF213
Cellular protein catabolic process	518	1.6	8	0.000158	0.0433	UBC, PSEN1, SUMO1, CDK1, CUL1, NEDD4L, USP36, USP40
Transcription initiation from RNA polymerase II promoter	219	0.674	5	0.00054	0.0492	UBC, SMAD3, MED16, NEDD4L, TAF2
Positive regulation of cytoskeleton organization	110	0.339	4	0.000356	0.0487	MAPT, SMAD3, DLG1, FMN1
Pyrimidine nucleotide metabolic process	50	0.154	3	0.000483	0.0492	DHODH, NME3, NME4

Table 15. Ubiquitination, polyubiquitination and polyubiquitinated pTau according Age of Onset in PSEN1 E280A FAD

Group	N	20S Chymotrypsin activity			p value vs		N	Poly-Ubiquitinated			p value vs	
		Average	SD	Range	E-AOFAD	LOFAD		Average	SD	Range	E-AOFAD	LOFAD
**Ctrl**	4	173056.25	49135.24	123415.00 - 238034.00	--	--						
**E-AOFAD**	6	120305.17	48861.41	69496.00 - 196495.00	--	*0.028*	5	2.60	1.03	1.22 - 3.98	--	*0.042*
**LOFAD**	8	215598.88	99366.75	110872.00 - 374125.00	*0.028*	--	7	1.48	0.42	1.07 - 2.12	*0.042*	--
**FAD**	14	174758.71	92896.94	69496.00 - 374125.00			12	1.95	0.90	1.07 - 3.98		

Group	N	IP pTau S400/Tau			p value vs		IP pTau S422/Tau			p value vs	
		Average	SD	Range	E-AOFAD	LOFAD	Average	SD	Range	E-AOFAD	LOFAD
**E-AOFAD**	5	0.93	0.11	0.78 - 1.08	--	0.570	1.11	0.18	0.93 - 1.31	--	*0.004*
**LOFAD**	7	1.04	0.25	0.74 - 1.33	0.570	--	0.76	0.12	0.58 - 0.90	*0.004*	--
**FAD**	12	0.99	0.20	0.74 - 1.33			0.91	0.23	0.58 - 1.31		

Group	N	pTau Seeding Frontal Cortex			p value vs				pTau Seeding Temporal Cortex			p value vs			
		Average	SD	Range	Ctrl	SAD	E-AOFAD	LOFAD	Average	SD	Range	Ctrl	SAD	E-AOFAD	LOFAD
**(-) Ctrl**	2	0.00	0.003	0.00 – 0.00	--	*0.004*	*0.000*	*0.000*	0.00	0.000	0.00 – 0.00	--	0.201	*0.031*	*0.034*
**SAD**	2	1.00	0.000	1.00 – 1.00	*0.004*	--	*0.012*	0.473	1.00	0.000	1.00 – 1.00	0.201	--	0.737	0.737
**E-AOFAD**	5	1.65	0.323	1.29 – 2.14	*0.000*	*0.012*	--	*0.012*	1.37	0.380	1.08 – 2.02	*0.031*	0.737	--	0.831
**LOFAD**	5	1.14	0.121	0.95 – 1.27	*0.000*	0.473	*0.012*	--			0.49 – 2.19	*0.034*	0.737	0.831	--
					**Ctrl**	**SAD**						**Ctrl**	**SAD**		
**FAD**	10	1.40	0.222	0.95 – 2.14	*0.004*	0.295			1.34	0.485	0.49 – 2.19	*0.007*	0.342		

Table 16. Primary antibodies used in the study

Antigen (name)	kDa	Type	Host	Isotype	Immunogen / Epitope	Brand/Provider	Reference	Application (Dilution)	Specificity	Method
Anti-APP (6E10)	4-110	Monoclonal	Mouse	IgG1	Amino acid residue 1-16 of beta amyloid	Covance	SIG-39320	WB(1:1000), IHC(1:100)	H	IHC, WB
Anti-Abeta 1-42	4-110	Monoclonal	Mouse			Jansen	JRF/cAb42/26	IHC(1:100)	H	IHC
Anti-pTau (AT8)	37-46	Monoclonal	Mouse	IgG1	Amino-acids. around phosphorylated Serine 202 and Threonine 205	Thermo	MN1020	IHC (1:1500)	H, M, R, O	IHC
Anti-Tau (Tau	37-46	Monoclonal	Mouse	IgG1	Purified microtubule associated proteins	Thermo	MA1-26600	WB(1:1000)	H, M, R, O	WB
Anti-pTau (Ser400)	37-46	Polyclonal	Rabbit	IgG	Amino acid residue Ser400	Thermo	PA1-26693	WB(1:1000)	H	WB
Anti-pTau (Ser422)	37-46	Polyclonal	Rabbit	IgG	Amino acid residue Ser422	Thermo	OPA1-03151	WB(1:1000	H, M,R, O	WB
Anti-AKT (4OD4)	60	Monoclonal	Mouse	IgG1	C-Terminal	Cell Signaling	2920	WB(1:1000)	H, M, R, O	WB
Anti-pAkt (Ser473) (D9E)	60	Monoclonal	Rabbit	IgG1	Amino acid residue Ser473	Cell Signaling	4060	WB(1:1000)	H, M, R, O	WB
Anti-pGSK-3β (Ser9) (5B3)	46	Monoclonal	Rabbit	IgG	Amino acid residue Ser9	Cell Signaling	9323	WB(1:1000)	H, M, R, O	WB
Anti-pGSK-3β (Y216) Clone 13A	46	Monoclonal	Mouse	IgG1	Rat GSK-3β (pY216) Peptide	BD Biosciences	612313	WB(1:1000)	H, M, R, O	WB
Anti-GSK-3β (27C10)	46	Monoclonal	Rabbit	IgG	Full length	Cell Signaling	9315	WB(1:1000)	H, M, R, O	WB
Anti-pMEK1/2(Ser217/221)	45	Polyclonal	Rabbit	IgG	Residues surrounding Ser217/221 of H-MEK1/2.	Cell Signaling	9121	WB(1:1000)	H, M, R, O	WB
Anti-MEK1/2 (L38C12)	45	Monoclonal	Mouse	IgG1	Full length	Cell Signaling	4694	WB(1:1000)	H, M, R, O	WB
Anti-pERK1/2 (Thr202/Tyr204) (E10)	42/44	Monoclonal	Mouse	IgG1	Residues surrounding Thr202/Tyr204	Cell Signaling	9106	WB(1:1000)	H, M, R, O	WB
Anti- ERKk1/2	42/44	Polyclonal	Rabbit	IgG	C-Terminal	Cell Signaling	9102	WB(1:1000)	H, M, R, O	WB
Anti-pCaMKIIa	52,5	Polyclonal	Rabbit	IgG	Residues surrounding Thr286 of H- CaMKII	Cell Signaling	3361	WB(1:1000	H, M, R, O	WB
Anti-CaMKIIa	52,5	Monoclonal	Mouse	IgG2a	Purified rat brain CaM Kinase.	Invitrogen	137300	WB(1:1000	H, M, R, O	WB
Anti-pJNK	46-54	Polyclonal	Rabbit	IgG	Residues surrounding Thr183/Tyr185 of H-SAPK/JNK	Cell Signalling	9255	WB(1:1000	H, M, R, O	WB
Anti-JNK	46-54	Polyclonal	Rabbit	IgG	Recombinant human JNK2 fusion protein	Cell Signalling	9252S	WB(1:1000	H, M, R, O	WB
Anti-CDK5	30	Polyclonal	Rabbit	IgG	Full length	Cell Signaling	2506	WB(1:1000)	H, M, R, O	WB
Anti-Fyn	59	Polyclonal	Rabbit	IgG	Residues surrounding Ser25	Cell Signaling	4023	WB(1:1000)	H, M	WB
Anti-methyl-PP2A (2A10)	36	Monoclonal	Mouse	IgG1	Methylated form of PP2A, catalytic subunit	Millipore	04-1479	WB(1:1000)	H, M, R, O	WB
Anti-GAPDH	38	Monoclonal	Mouse	IgG1	GAPDH from rabbit muscle.	Millipore	MAB 374	WB(1:100 - 300)	H, M, R, O	WB
Anti-mono and Polyubiquitin conjugates	> 8,5	Monoclonal	Mouse	IgG1	(FK2) Poly –ubiquitinylatedlysozyme	Enzo life Sci.	BML-PW8810	WB(1:1000)	H, M, R, O	WB, IP
Anti-Synaptophysin (YE269)	NA	Monoclonal	Rabbit	IgG	Human Synaptophysin aa 250 to C-terminus	Abcam	32127	IF (1:50)	H, M, R, O	IF

Table 17. Secondary antibodies used in the study

Antigen (Name)	Host	Isotype	Brand/Provider	Reference	Application (Dilution)	Specificity	
Anti-Mouse-HRP	Goat	IgG (H+L)	Invitrogen	G21040	WB(1:2500)	Mouse	WB
Anti-Rabbit-HRP	Goat	IgG (H+L)	Invitrogen	G21234	WB(1:2500)	Rabbit	WB

WB: Western blot

IHC: Immunohistochemistry

Human, Mouse, Rat, Others: H, M, R, O

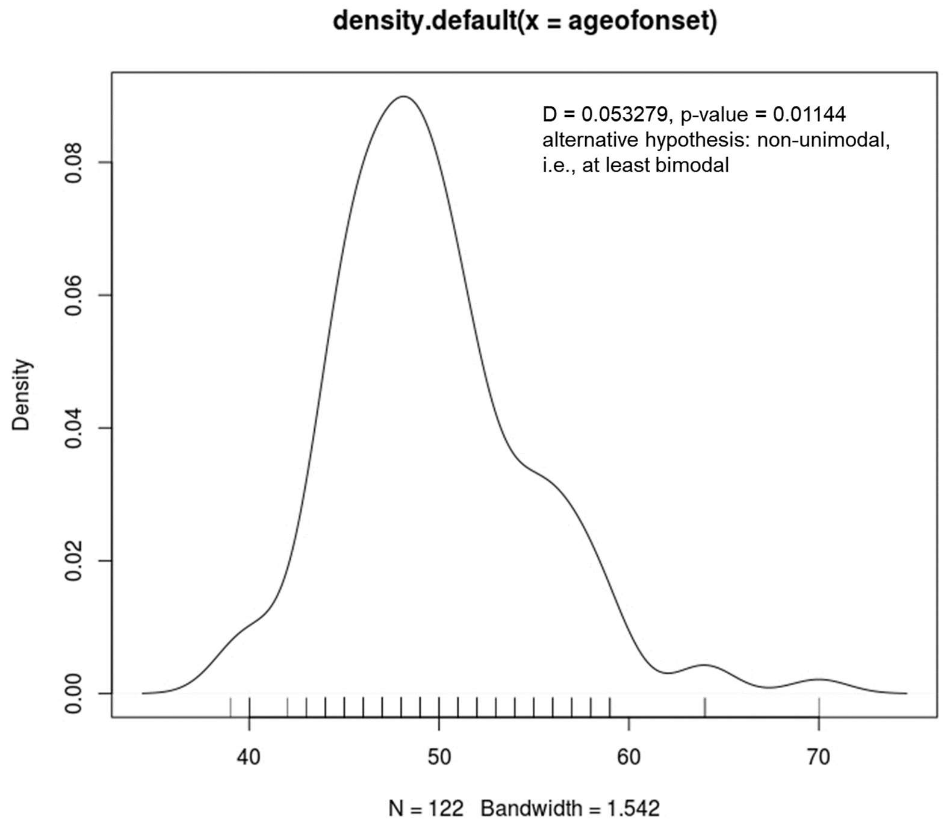

Figure 1. AoO distribution in PSEN1 E280A. Unimodality Hartigan's dip test for AoO in 122 PSEN1 E280A dementia patients. p value< 0.05 indicates multimodal distribution.

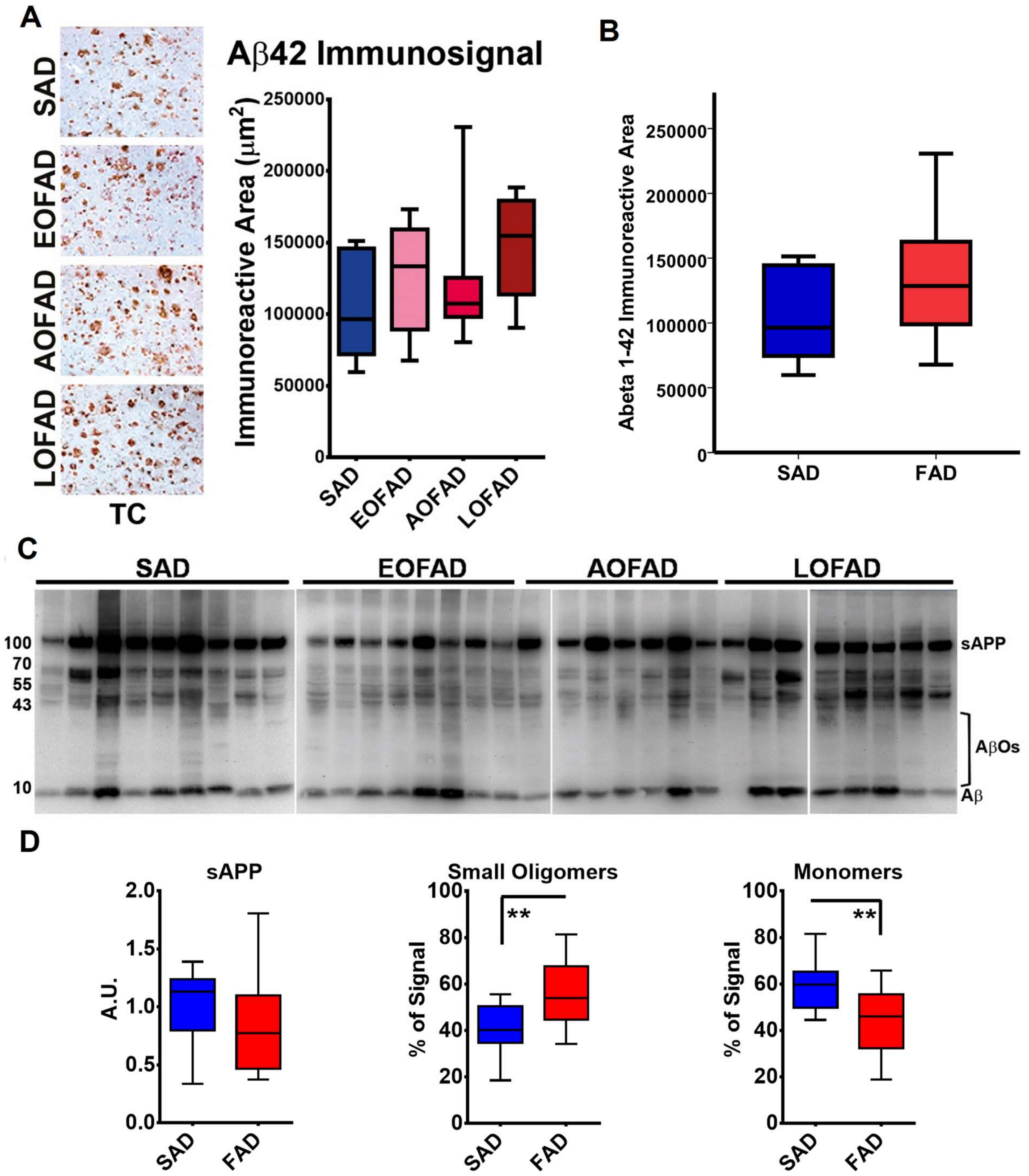

Figure 2. Aβ pathology in AD cases. A. Quantification of Ab 1-42 plaque loads present in frontal cortex. There were not significant differences between groups. B.Quantification of Ab 1-42 plaque load in temporal cortex in SAD patients (n=10) vs PSEN1 E280A FAD patients (n=23). There is not significant differences between groups. C. **Densitometric analysis of sAPP, small Ab oligomers and Ab monomers.** Semi denaturing electrophoresis of TBS soluble fractions from temporal cortex in SAD patients (n=10) vs PSEN1 E280A FAD patients (n=23) were blotted with 6E10 antibody and analyzed according to their band distribution in kDa. D. Densitometric analysis scatterplots for sAPP, small AβOs and Aβ monomers. There are not significant differences between groups for sAPP, both oligomers and monomers show differences between SAD and FAD patients (**=p≤0.01).

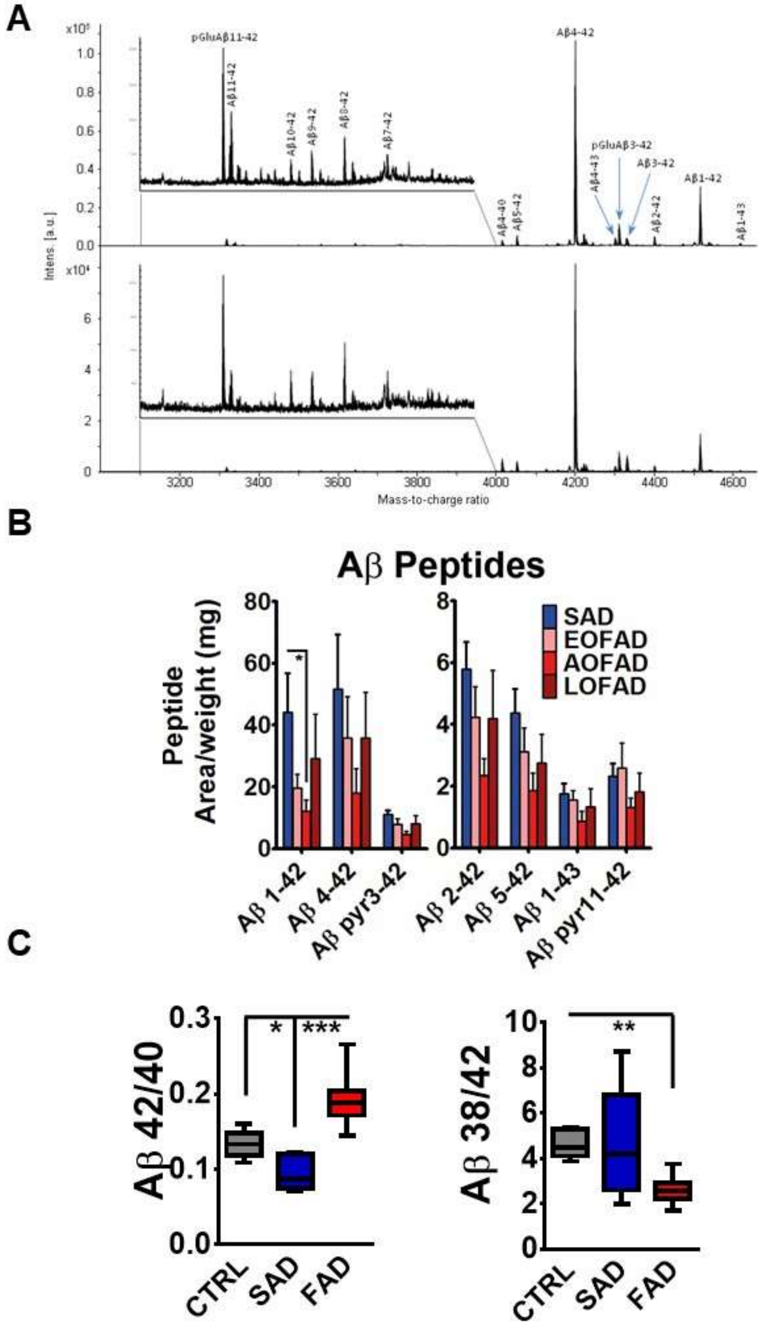

Figure 3. Aβ peptides and enzymatic turnover of gamma secretase of SAD and FAD cases. A. Mass Spect profile of Aβ peptides found in temporal cortex of PSEN1 E280A FAD patients. B. Graph bars depicting Aβ peptides levels as evaluated by mass spectrometry analysis on temporal cortex from SAD (n=10), EOFAD (n=8), AOFAD (n=7) and LOFAD (n=8) cases. Bars depict means +/- SEM. There were not significant differences between AoO FAD groups. SAD cases presented significantly higher levels of Aβ 1-42 compared with AOFAD cases (* = $p \leq 0.05$). C. Significantly increased ratio of de novo generated 42/40 Aβ peptides in PSEN1E280A FAD patients (n=23) when compared to Control (n=5) and SAD cases (n=5). Significantly decreased ratio of de novo 38/42 Aβ peptides when compared to Control cases. (** = $p \leq 0.01$, *** = $p \leq 0.001$).

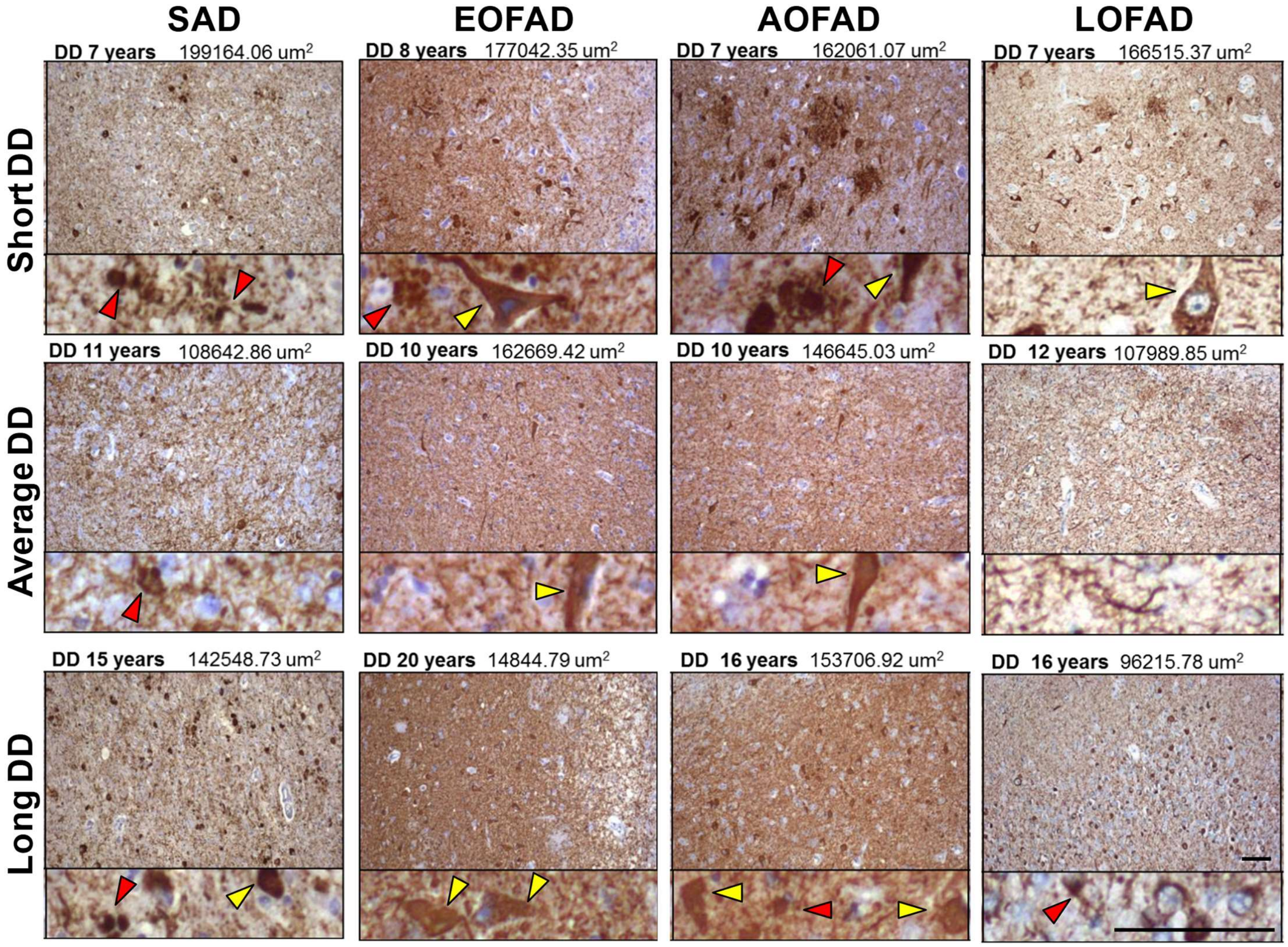

Figure 4. pTau pathology according to disease duration and Age of Onset in PSEN1 E280A FAD patients. Immunohistochemical staining for pTau using AT8 antibody in temporal cortex of selected cases from all groups according to their comparable disease duration (DD) long, average, or short. pTau loads differ with EOFAD and AOFAD showing in general higher loads and more extracellular distribution independently of DD, together with dystrophic neurites (red arrowheads) and NFTs (yellow arrowheads). (scale bar = 40 mm).

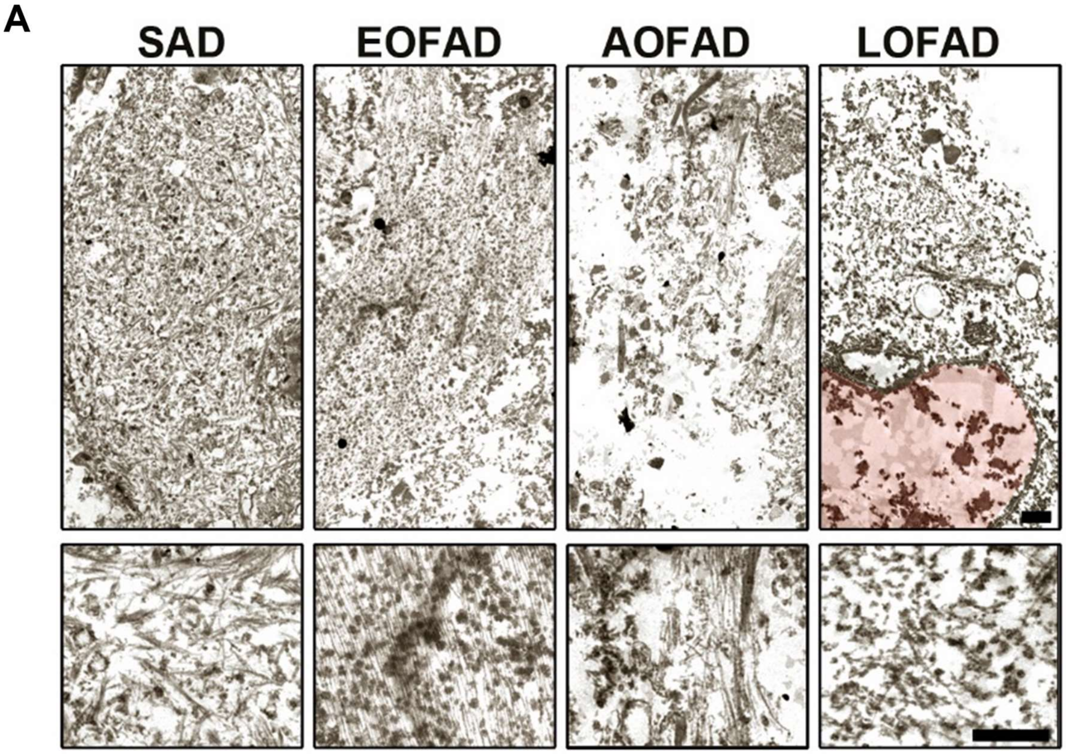

Figure 5. Ultrastructural analysis of temporal cortex from the SAD and AoO groups of *PSEN1* E280A FAD.SAD, EOFAD and AOFAD cases showed extracellular paired helical fragments as depicted at higher magnification in lower panels. LOFAD cases do not show such Tau aggregates. (Scalebar = 2 mm, Nucleus in red).

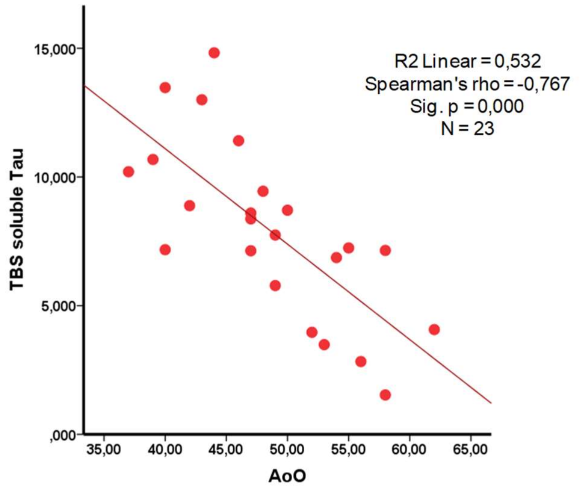

Figure 6. Correlation analysis between levels of total Tau in TBS soluble fractions and AOO in PSEN1 FAD patients.

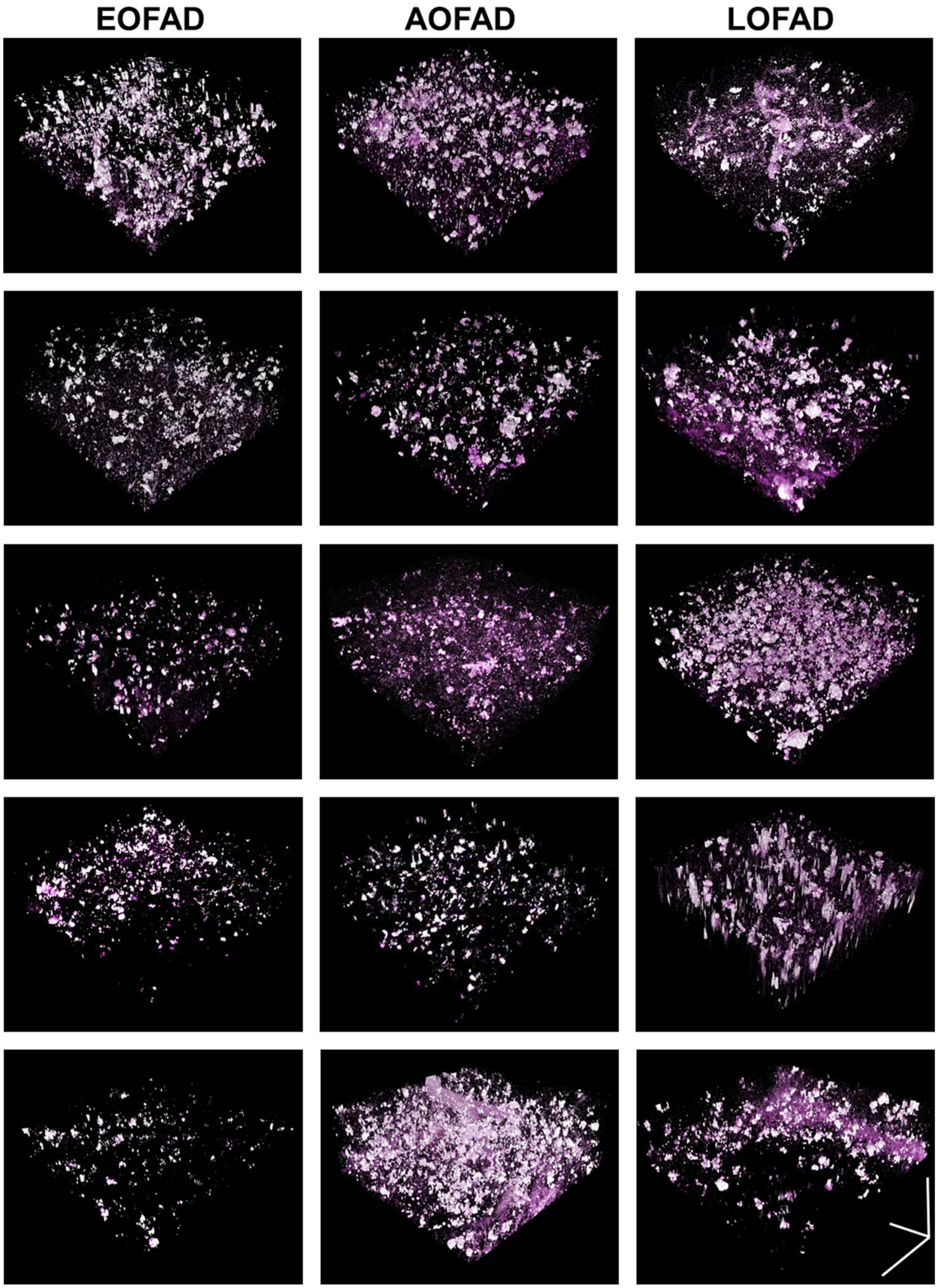

Figure 7. Representative 3D rendering of clarified formalin fixed temporal cortices from PS1E280A FAD cases stained for synaptophysin and grouped by age of onset. Scale bars=80 μm.

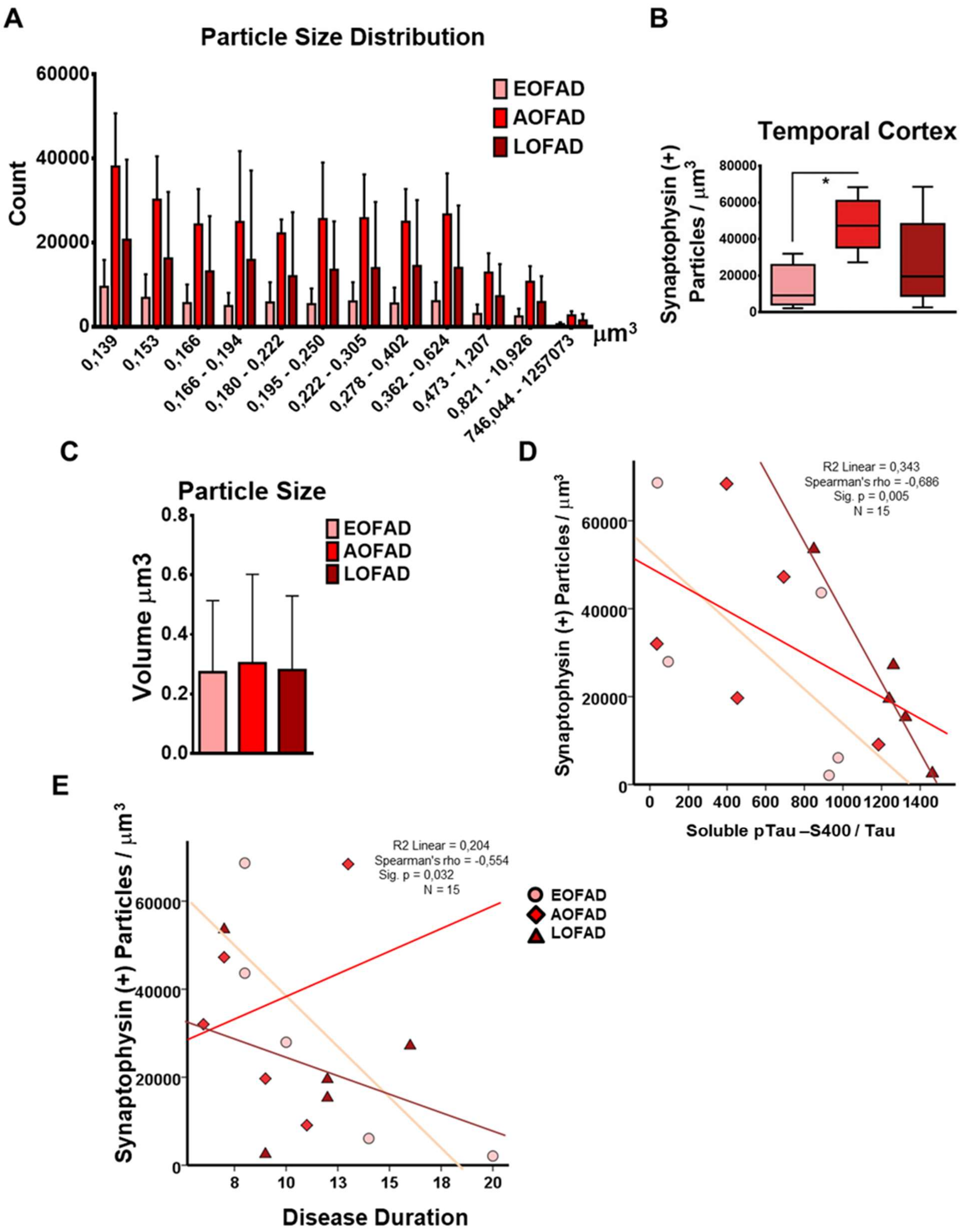

Figure 8. Synaptophysin particle distribution according to size in PS1 E280A FAD cases grouped by age of onset. A. Particle count according to size in PS1 E280A FAD cases grouped by age of onset. B. Average Synaptophysin-positive particle size in PS1 E280A FAD cases grouped by age of onset. C. Bar graphs for the density of small Synaptophysin-positive particles in temporal cortices of EOFAD (n=5), AOFAD (n=5) and LOFAD (n=5) cases. AOFAD cases showed significantly higher particle density when compared with EOFAD cases. (* = $p \leq 0.05$). D. Correlation analysis between Synaptophysin particles density and soluble pTau-S400 / Tau. E. Correlation analysis between Synaptophysin particles density and disease duration.

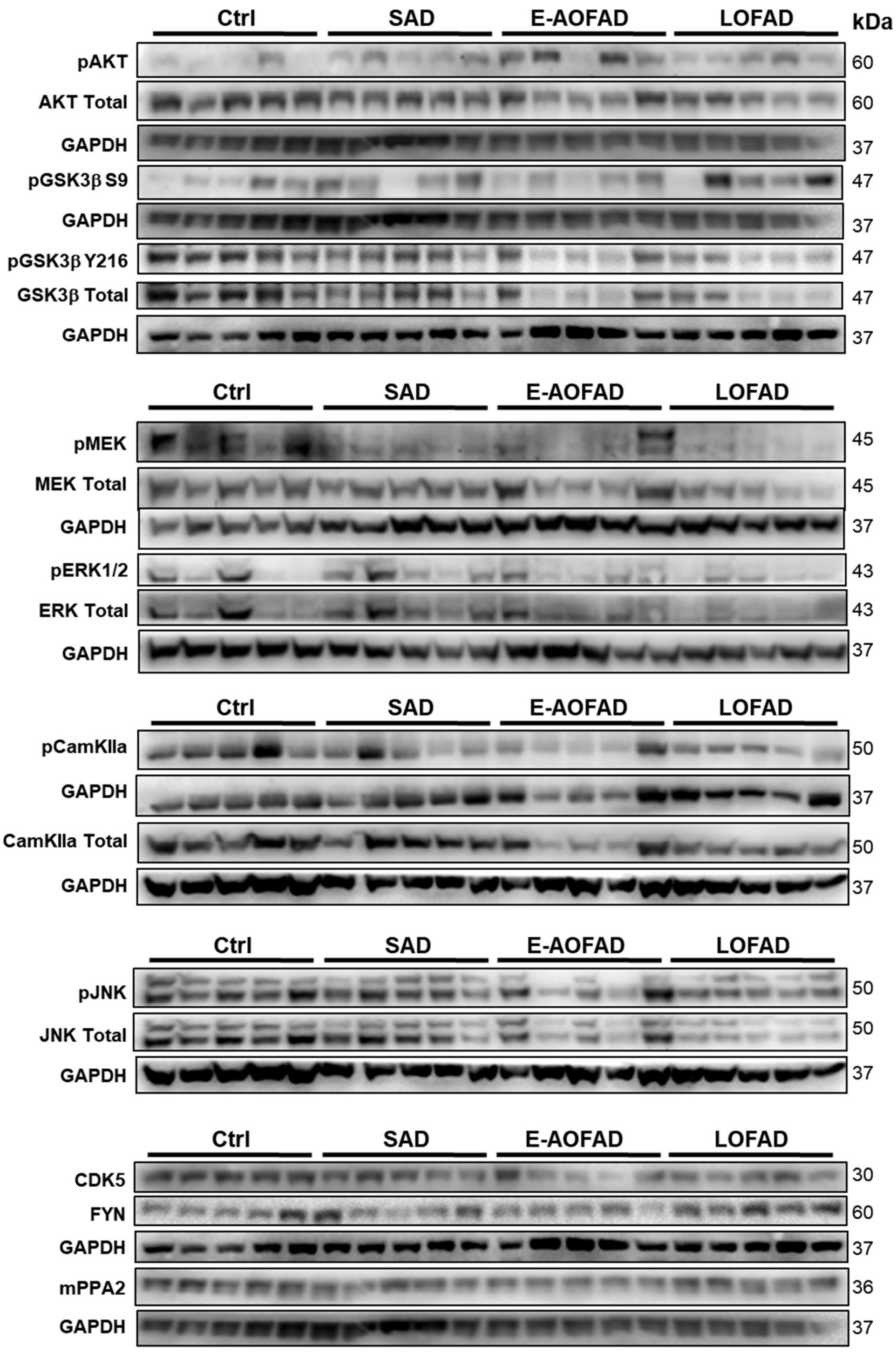

Figure 9. Representative blots of studied pTau-related kinases in Temporal cortex of controls, SAD and FAD cases

A

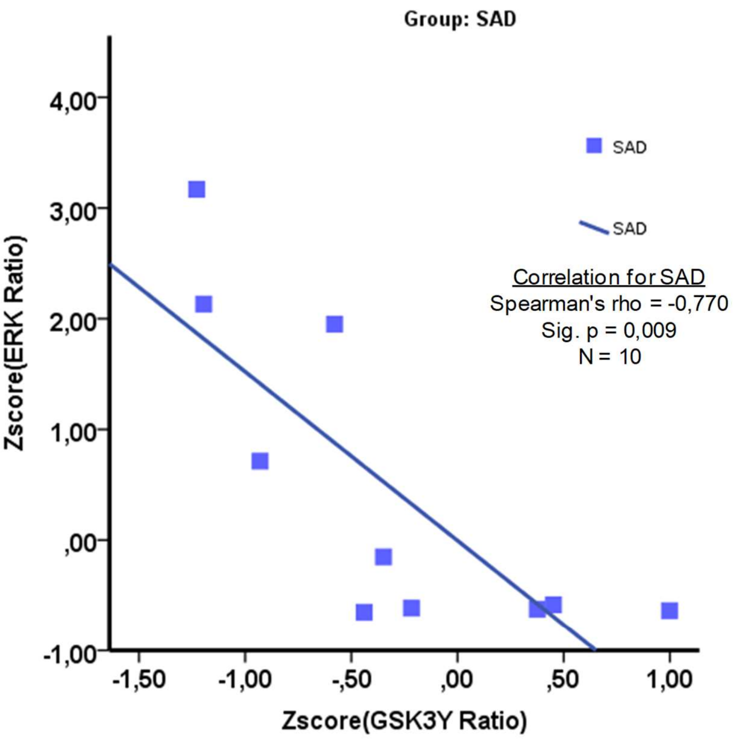

B

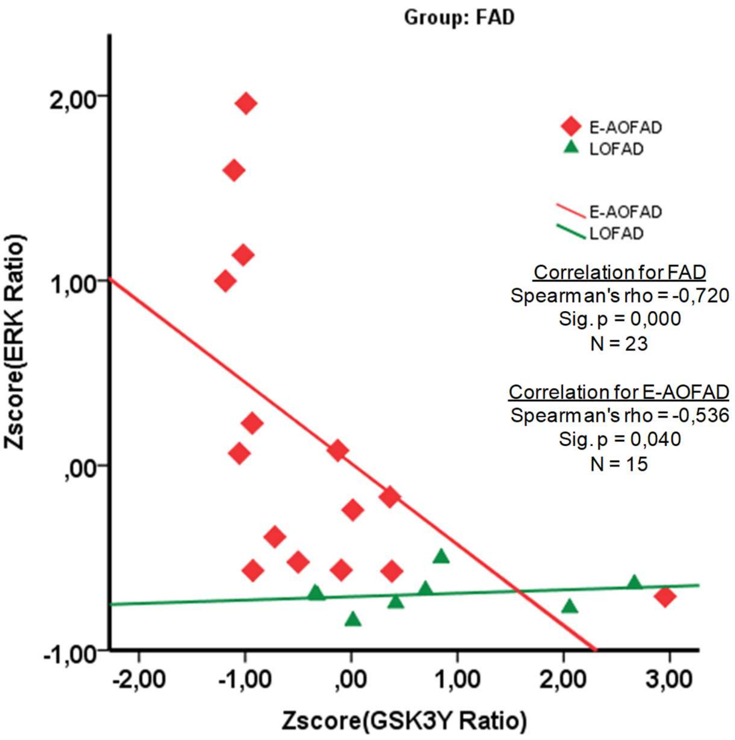

Figure 10. Correlation of normalized pERK1/2 / ERK1/2 ratio normalized GSK3b Y216 / GSK3b levels. A. SAD cases B. PSEN1E280A FAD cases. Colored lines depict group or subgroups tendencies.

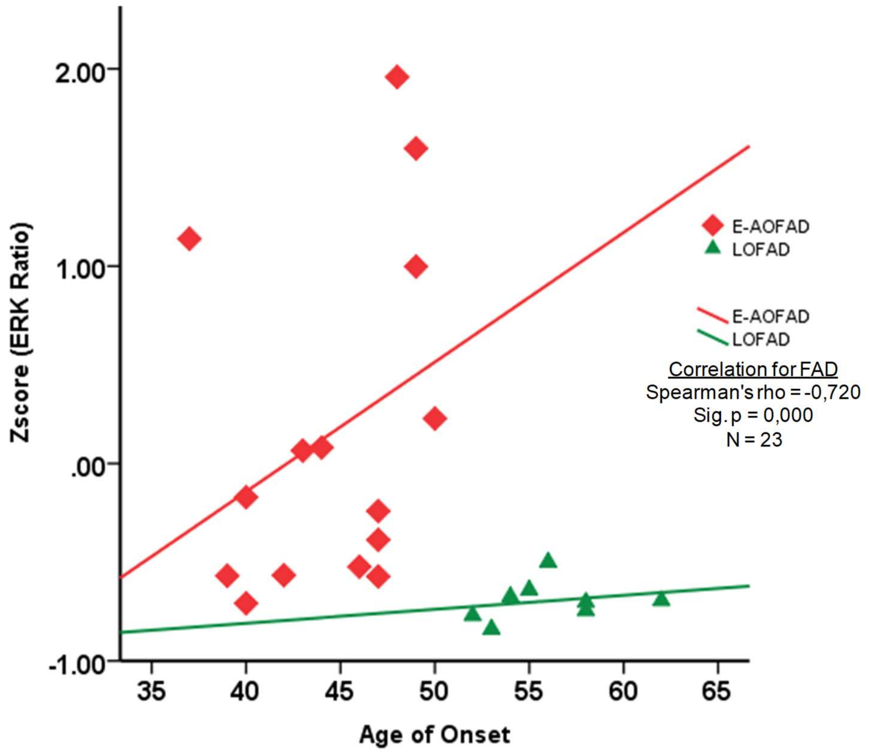

Figure 11. Correlation of normalized pERK1/2 / ERK1/2 ratio and age of onset. Colored lines depict group or subgroups tendencies.

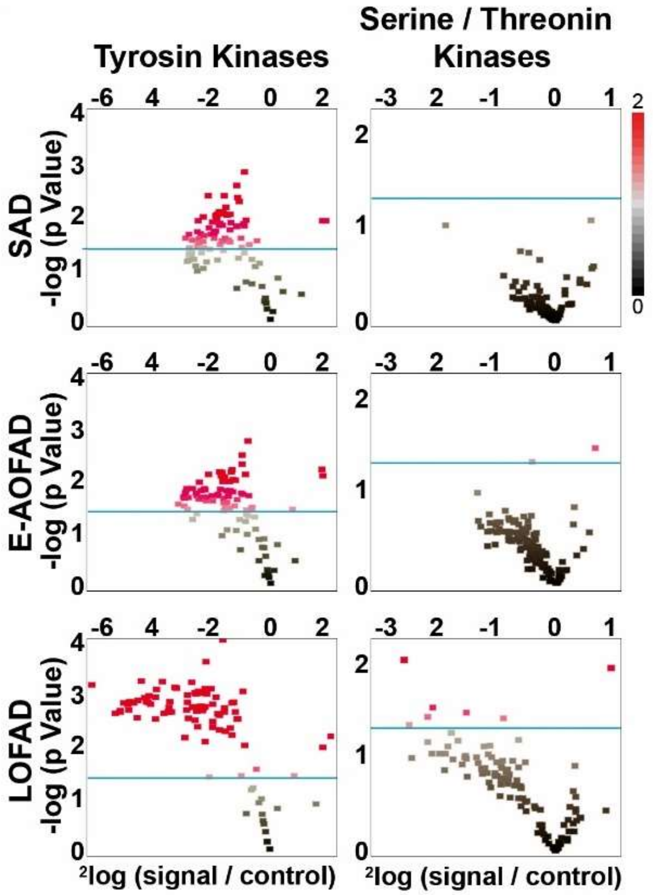

Figure 12. Significantly different active Tyrosin and Serine/Threonine kinases between FAD cases and controls. Volcano plot graph for significantly phosphorylated peptides in AD groups when compared against Control. Blue line represents significance threshold. Only LOFAD shows noticeable differences when compared with Control.

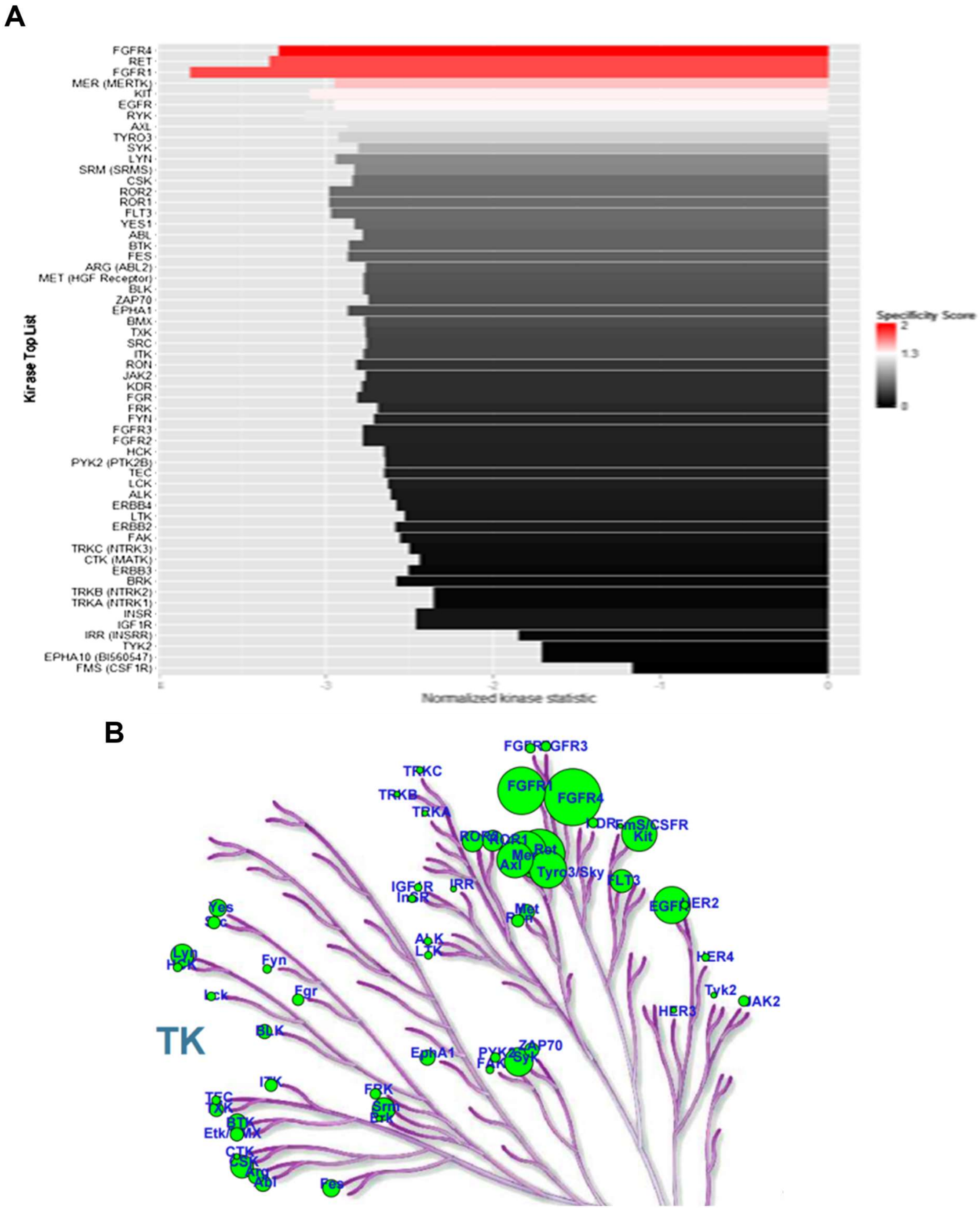

Figure 13. Upstream kinase analysis of Tyrosine Kinases Ctrl vs LOFAD cases and dendritic tree of significantly specific kinases. A. Bar plot of tyrosine kinases according to their score in the upstream kinase analysis for differences between controls and LOFAD cases. Length of each bar shows normalized kinase change between groups. Positive values indicate that this associated kinase activity was higher in LOFAD than in controls a negative value indicates the opposite. Color of the bars indicates specificity of the kinase set. B. Dendrogram of related siginificantly different tyrosine kinases in PSEN1E280A LOFAD cases. Size of green bubble indicates specificity.

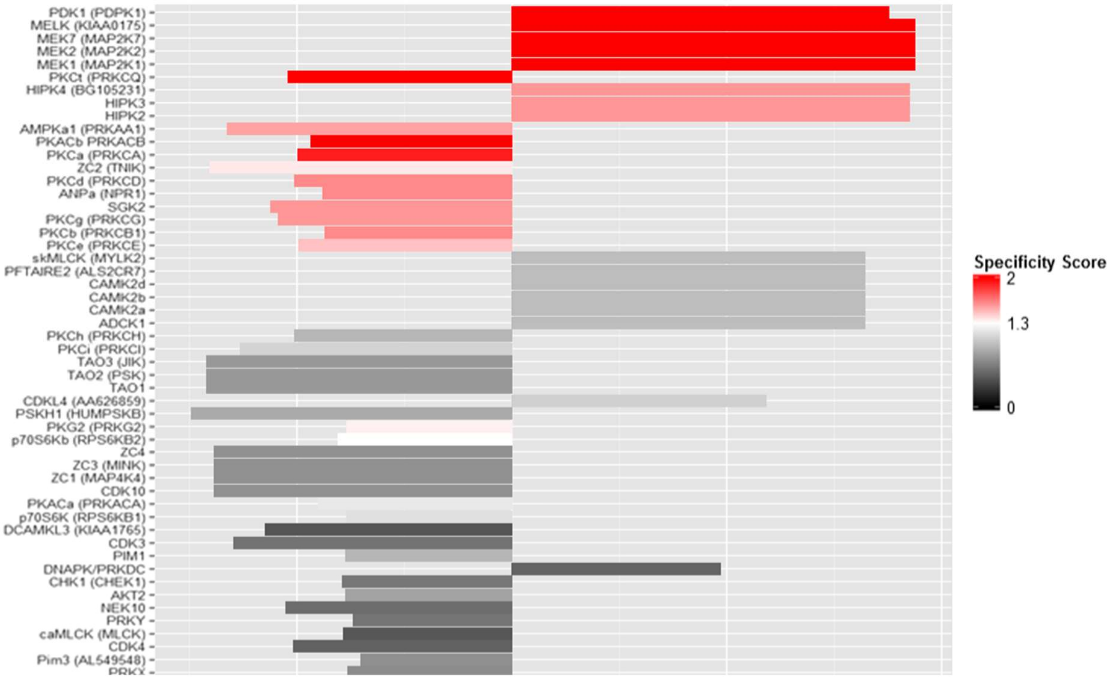

Figure 14. Upstream kinase analysis of Serine / Threonine Kinases Ctrl vs LOFAD cases and dendritic tree of significantly specific kinases. Bar plot of serine / threonine kinases according to their score in the upstream kinase analysis for differences between controls and LOFAD cases. Length of each bar shows normalized kinase change between groups. Positive values indicate that this associated kinase activity was higher in LOFAD than in controls a negative value indicates the opposite. Color of the bars indicates specificity of the kinase set.

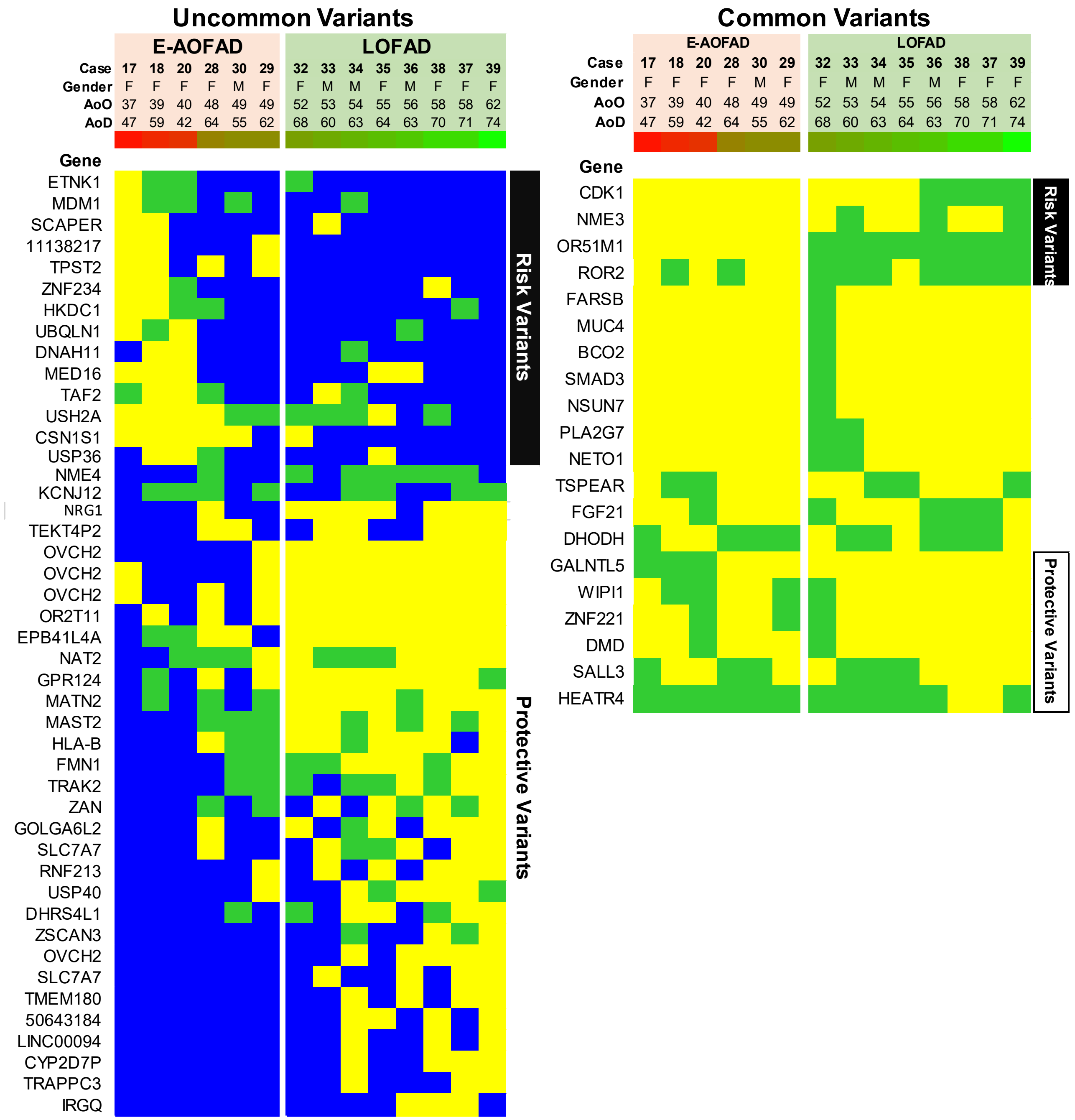

Figure 15. Demographic characteristics and distribution of risk / protective genetic variants according to age of onset in studied PSEN1 E280A cases. In pale red early and average onset cases and in pale green late onset cases. Variant homozygous = Yellow, Variant-Reference heterozygous = green, Reference homozygous = blue, AoO = Age of onset, AoD = Age of death.

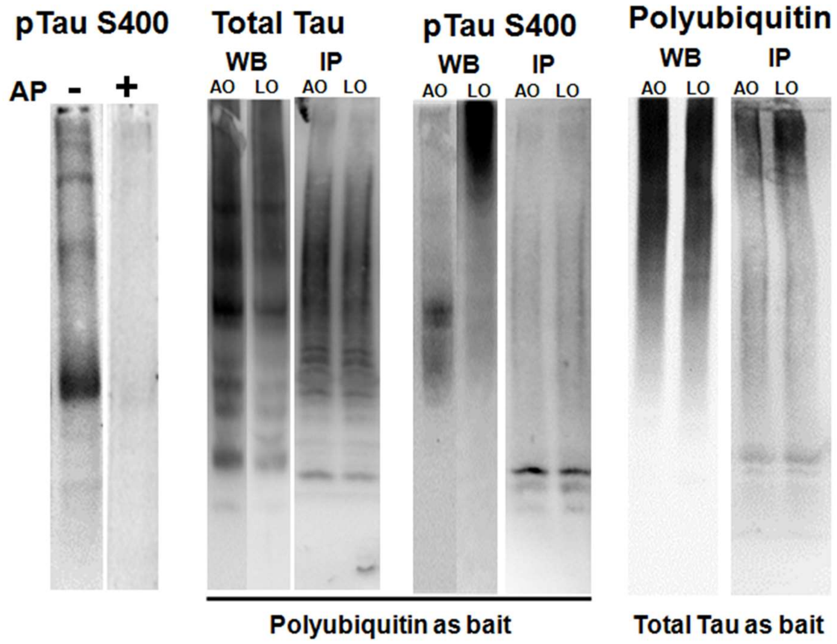

Figure 16. Proof of principle for co-immunoprecipitation with anti-polyubiquitinated and anti-Tau antibodies in brain tissue. First, we tested the specificity of the pTau S400 antibody using temporal cortex total homogenate and dephosphorylating the membrane with Akaline phosphatase (AP) 1u/ug of protein during 1 hour at 37 ^{0}C before incubation with primary and secondary antibodies. Afterwards, using temporal cortex TBS fractions from one average onset and one late onset FAD cases we tested immunoprecipitation and western blots using antibodies against total Tau and polyubiquitin as baits and blotting with total tau, pTau s400 and polyubiquitin. At the left side for each test we placed how standard western blot looks for each case.

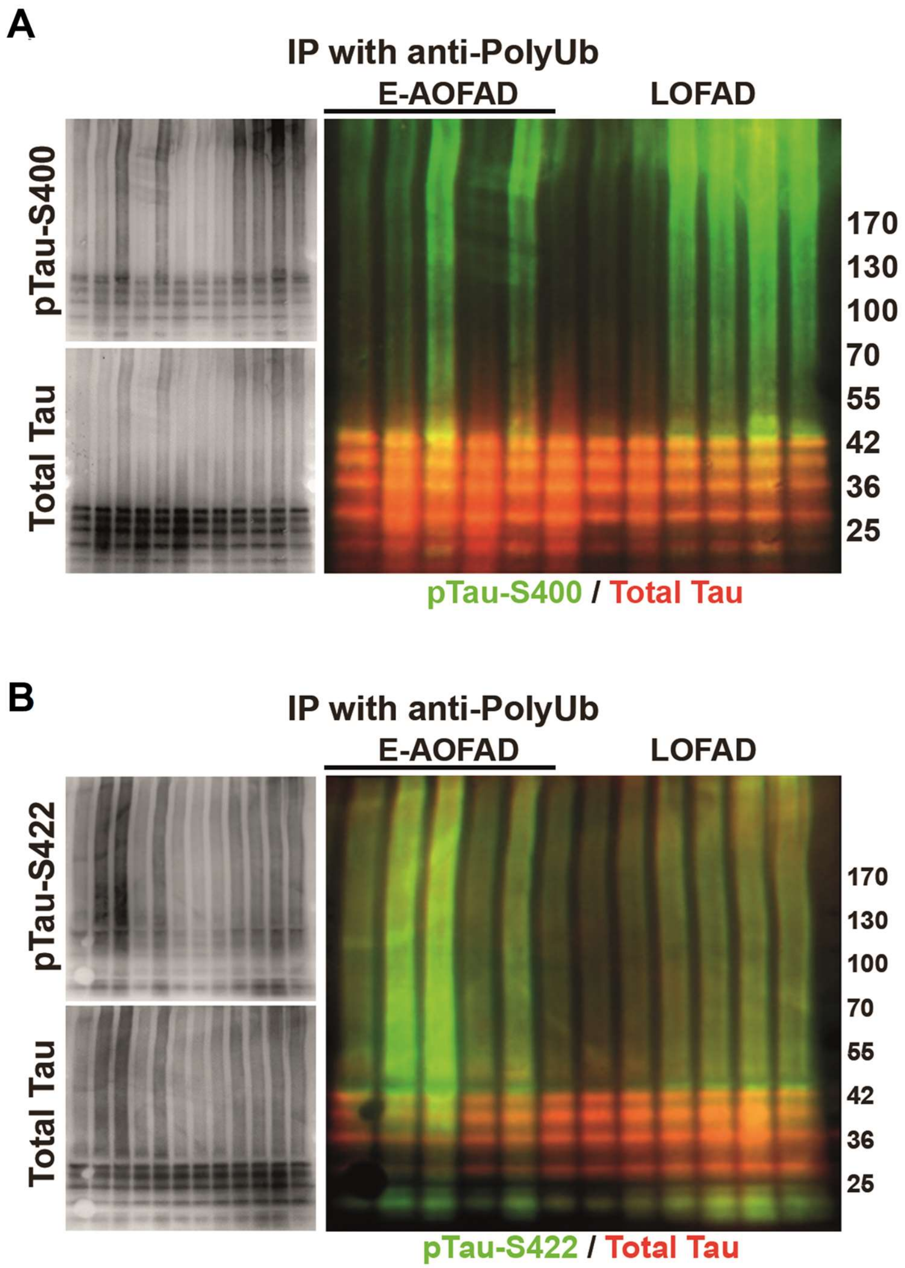

Figure 17. Co-immunoprecipitation using monoclonal polyubiquitin antibody as bait and immunoblots for total Tau, pTau-S400 and pTau-S422. A. Co-immunoprecipitation using monoclonal polyubiquitin antibody as bait and immunoblots for total Tau and pTau-S400 in TBS soluble fractions from temporal cortex of early and average AoO FAD (E-AOFAD, n = 5) and LOFAD (n = 7). B. Co-immunoprecipitation using monoclonal polyubiquitin antibody as bait and immunoblots for total Tau and pTau-S422 in TBS soluble fractions from temporal cortex of early and average AoO FAD (E-AOFAD, n = 5) and LOFAD (n = 7).

A

B

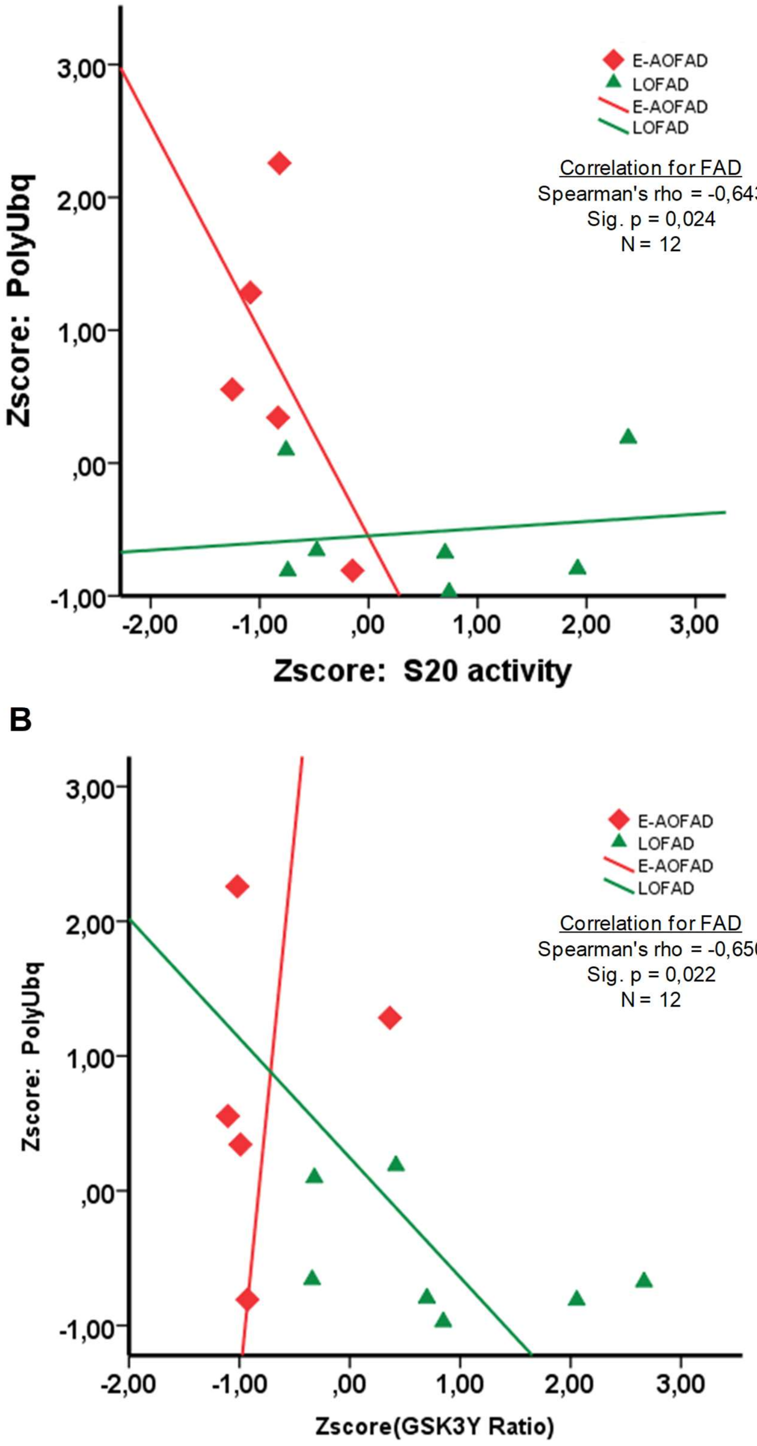

Figure 18. Correlation of polyubiquitinated proteins with S20 Chimotrypsin activity and normalized GSK3b Y216 / GSK3b levels. Colored lines depict group or subgroups tendencies.

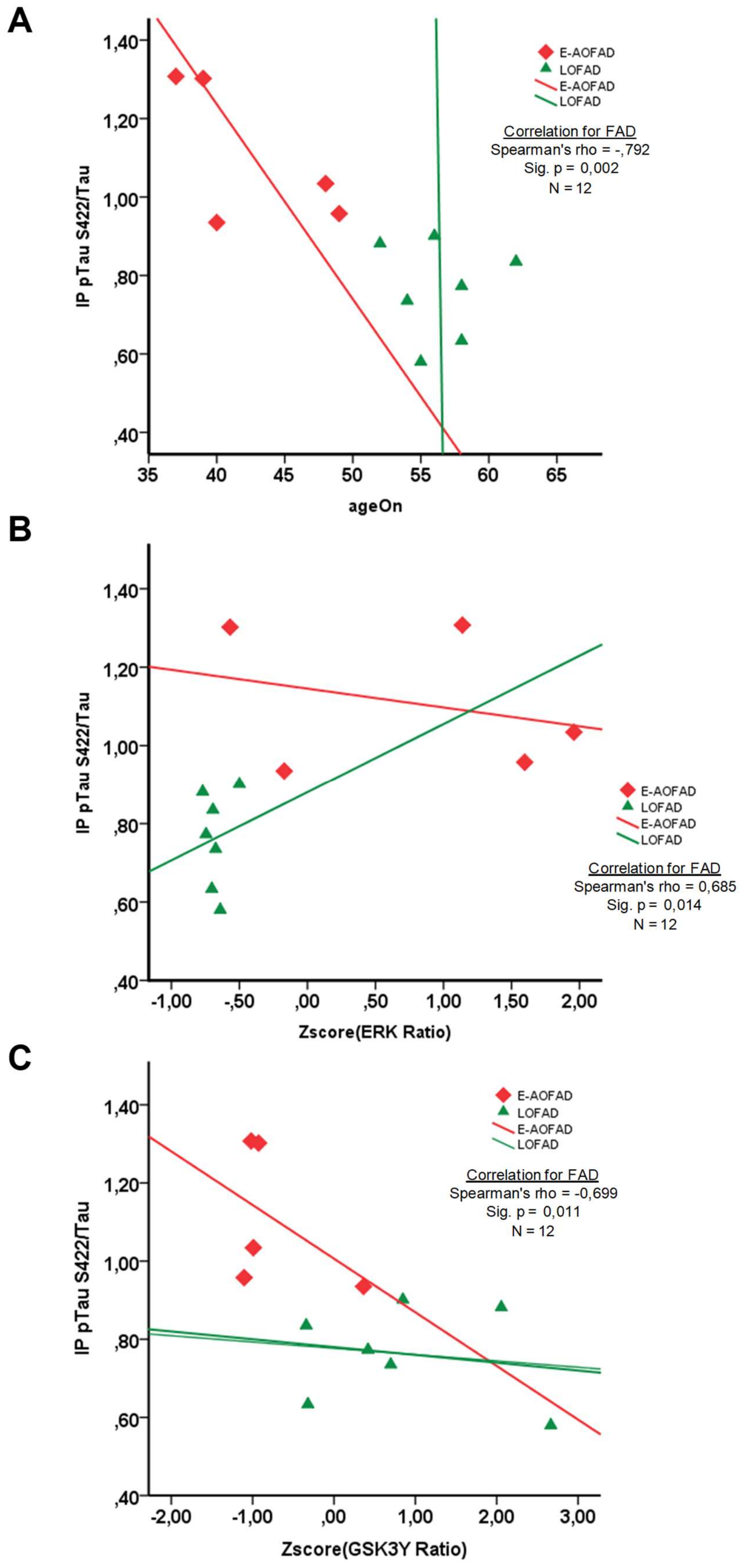

Figure 19. Correlation of immunoprecipitatedpTau S422/Tau with AoO, normalized pERK1/2 / ERK1/2 ratio and normalized GSK3b Y216 / GSK3b levels. Colored lines depict group or subgroups tendencies.

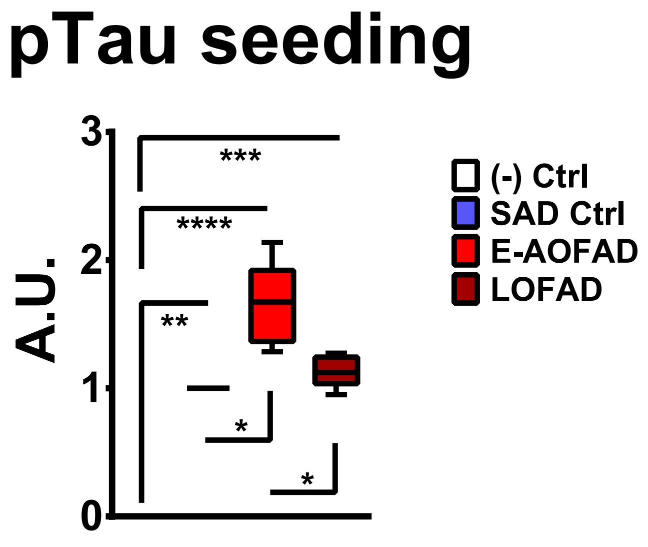

Figure 20. pTau seeding activity in frontal cortex. Bar graph representing pTau seeding capacity assays in temporal cortices homogenates from negative Controls (n=2), SAD (n=2), E-AOFAD (n=5) and LOFAD (n=5) cases. Both FAD groups showed increased pTau seeding activity when compared to negative controls and E-AOFAD showed increased activity compared to SAD samples. (* = $p \leq 0.05$, ** = $p \leq 0.01$, *** = $p \leq 0.001$, **** = $p \leq 0.0001$).
